# Insights on the Lewis Superacid Al(OTeF_5_)_3_: Solvent Adducts, Characterization and Properties**

**DOI:** 10.1002/chem.202201958

**Published:** 2022-08-18

**Authors:** Kurt F. Hoffmann, Anja Wiesner, Simon Steinhauer, Sebastian Riedel

**Affiliations:** ^1^ Fachbereich für Biologie Chemie Pharmazie Institut für Chemie und Biochemie – Anorganische Chemie Fabeckstraße 34/36 14195 Berlin Germany

**Keywords:** aluminum, coordination chemistry, fluorine chemistry, Lewis superacids, pentafluoroorthotellurate

## Abstract

Preparation and characterization of the dimeric Lewis superacid [Al(OTeF_5_)_3_]_2_ and various solvent adducts is presented. The latter range from thermally stable adducts to highly reactive, weakly bound species. DFT calculations on the ligand affinity of these Lewis acids were performed in order to rank their remaining Lewis acidity. An experimental proof of the Lewis acidity is provided by the reaction of solvent‐adducts of Al(OTeF_5_)_3_ with [PPh_4_][SbF_6_] and OPEt_3_, respectively. Furthermore, their reactivity towards chloride and pentafluoroorthotellurate salts as well as (CH_3_)_3_SiCl and (CH_3_)_3_SiF is shown. This includes the formation of the dianion [Al(OTeF_5_)_5_]^2−^.

## Graphical abstract

**The dimeric Lewis superacid** [Al(OTeF_5_)_3_]_2_ and its solvent adducts are ready to test their strength in terms of fluoride ion affinity. While some of them are still highly reactive, others had too much coordinating solvent and remain stable but comparably unreactive. In here, their preparation, characterization and reactivity are reported in detail.

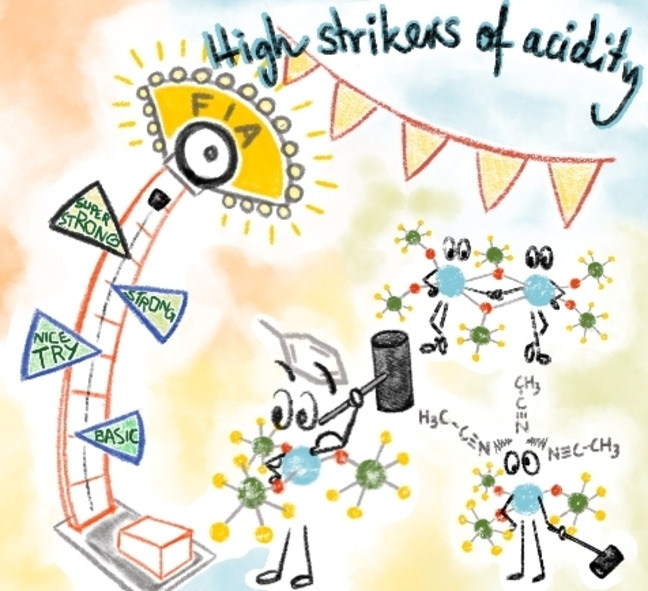

## Introduction

**Figure 1:**
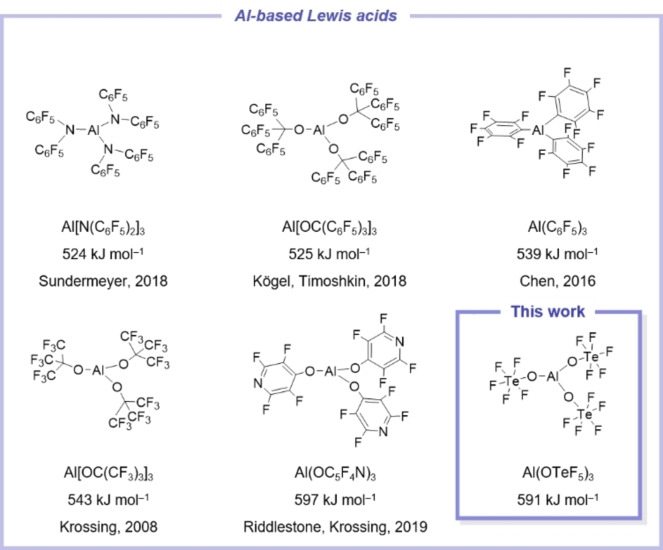
Modern aluminum‐based Lewis superacids and their corresponding fluoride ion affinity. FIA values were calculated on the BP86/def‐SV(P) level of theory. Me_3_SiF was used as anchor point.^[2]^

For a long time antimony pentafluoride was considered the strongest Lewis acid in the condensed phase.^[1]^ In the last two decades, a new class of Lewis superacids emerged, which are defined as Lewis acids with a higher fluoride ion affinity (FIA) than molecular SbF_5_ in the gas‐phase (calc. FIA:^[2]^ 493 kJ mol^−1^ on BP86/def‐SV(P); exptl. FIA:^[3]^ 506±63 kJ mol^−1^) and thereby surpass the latter in terms of acidity and manageability (cf. Figure 1).[^4^, ^5^] More recent examples of group 13 Lewis superacids are B(C_6_F_4_CF_3_)_3_,^[6]^ Al(C_6_F_5_)_3_ ^[7]^ (including partially fluorinated derivatives^[8]^), Al[OC(CF_3_)_3_]_3_,^[9]^ Al[N(C_6_F_5_)_2_]_3_,^[10]^ Al(OC_5_F_4_N)_3_,^[11]^ Al[OC(C_6_F_5_)_3_]_3_ ^[12]^ and a comprehensive review was recently published.^[13]^ However, one of the drawbacks of such compounds is their strong interaction with solvent molecules or their own ligand system, resulting in a drastically lowered Lewis acidity. Regarding the manageability, important properties of Lewis superacids are the isolability as a neat substance as well as the thermal stability.

In 2017 we reported first attempts of the synthesis of the Lewis acid Al(OTeF_5_)_3_.^[14]^ With a FIA of 591 kJ mol^−1^ for its molecular unit it can be counted as one of the strongest known isolable Lewis acids. The compound was analyzed by IR and Raman spectroscopy, revealing the dimeric form [Al(OTeF_5_)_3_]_2_ in the solid state. Still, its temperature sensitivity made the handling of the compound tedious as it rapidly decomposed at temperatures above 0 °C, which might be accounted to impurities (see below).

Herein we report on an improved synthesis of [Al(OTeF_5_)_3_]_2_, which can be prepared on a multigram scale. The Lewis superacid is room‐temperature stable for several hours and isolable as an adduct‐free, amorphous powder. With this neat Lewis acid in hand, we investigated its complexation with a broad range of different solvents, ranging from thermally stable, strongly bound adducts to weakly bound, reactive species. We then further elaborate on the reactivity of these so formed solvent‐adducts.

## Results and Discussion

In our previous study we firstly reported on the formation of the Lewis acid [Al(OTeF_5_)_3_]_2_ by the reaction of triethylaluminium, AlEt_3_, with teflic acid, HOTeF_5_, in *n*‐pentane in a stoichiometric ratio of 1 : 3.^[14]^ This reaction yields a colorless powder which is unstable at room temperature. Analyzing a solution of this product in SO_2_ClF by low‐temperature NMR spectroscopy revealed the presence of residual alkyl moieties that presumably led to a decreased thermal stability (decomposition above 0 °C) of this compound. Therefore, an improved synthesis of the dimeric [Al(OTeF_5_)_3_]_2_ by employing different reactants and conditions was needed.

**Scheme 1:**
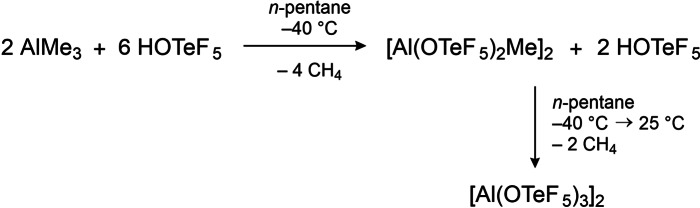
Improved synthesis of the Lewis acid [Al(OTeF_5_)_3_]_2_.

**Figure 2:**
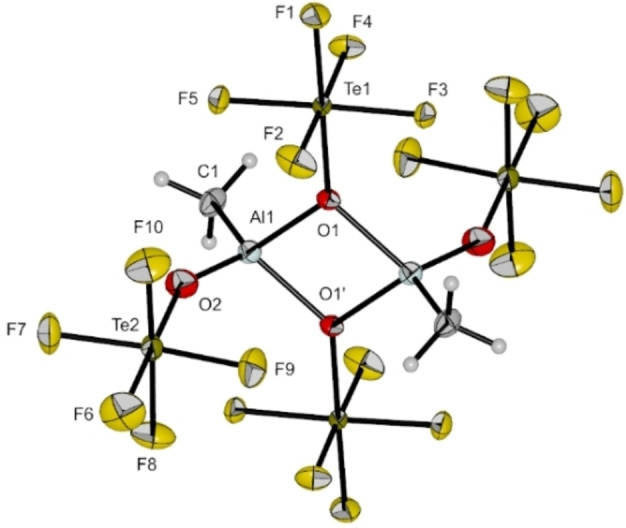
Molecular structure of [Al(OTeF_5_)_2_Me]_2_ in the solid state. Thermal ellipsoids set up to 50 % probability. Selected bond lengths [pm] and angles [°]: Al1‐O1 188.2(5), Al1‐O2 172.3(6), Al1‐O1’ 189.0(6), Al1‐C1 192.3(8), O1‐Al1‐C1 117.1(3), O1‐Al1‐O2 107.1(3), O1‐Al1‐O1’ 79.2(2), C1‐Al1‐O2 118.3(4), C1‐Al1‐O1’ 118.5(3), O2‐Al1‐O1’ 110.1(3).

In our new approach AlMe_3_ was used as a starting material (cf. Scheme 1). Analogue to the reported synthesis with AlEt_3_, treatment in *n*‐pentane with 3 equivalents of HOTeF_5_ and warming of the mixture from −196 °C to −40 °C results in the formation of a colorless precipitate. After removing the solvent, a yet again temperature‐sensitive powder remains. Low‐temperature NMR measurements in SO_2_ClF show the presence of a methyl group at −0.01 ppm in the ^1^H NMR spectrum, indicating an incomplete substitution of the methyl groups by −OTeF_5_ (teflate) groups at the aluminum center. In the ^19^F NMR spectrum, two sets of signals for magnetically inequivalent −OTeF_5_ groups are observed, while the ^27^Al NMR shows a broad signal at 48 ppm, typical for a tetrahedrally coordinated Al center. Single crystals suitable for low‐temperature single crystal X‐ray diffraction grew in a cooled *ortho*‐difluorobenzene (*o*‐DFB) solution and the molecular structure of the neutral dimer [Al(OTeF_5_)_2_Me]_2_ was obtained (cf. Figure 2). The dimer bridged by two −OTeF_5_ groups crystallizes in the monoclinic space group *P*2_1_/*n*.

Each of the aluminum centers is coordinated by a methyl group, a terminal teflate group and two bridging teflate moieties, leading to a heavily distorted tetrahedral coordination sphere with bond angles between 79.2(2)° and 118.5(3)°. The bridging Al−O bond distances are elongated (*d*(Al1‐O1)=188.2(5), *d*(Al1‐O1’)=189.0(6) pm) compared to the terminal Al−O bond distance (*d*(Al1‐O2)=172.3(6) pm). The latter are comparable to Al−O bond distances in salts of the anion [Al(OTeF_5_)_4_]^−^ (e. g., *d*(Al−O) in [PPh_4_][Al(OTeF_5_)_4_]=173.4(2) pm).^[14]^ This difference in bond distances is analogue to the difference in bond distances of bridging and terminal perfluoroalcoholates in the compound Et_2_Al(*μ*‐OR_f_)_2_Al(Et)(OR_f_) with OR_f_=OC(CF_3_)_3_) reported by Krossing et al.^[15]^ The Al−C bond distances (*d*(Al1‐C1)=192.3(8) pm) are shortened when compared to the molecular structure of dimeric [AlMe_3_]_2_, underlining the increased Lewis acidity of the Al centers in [Al(OTeF_5_)_2_Me]_2_.^[16]^ Recently, we reported on a comparable molecular structure of the higher homologue gallium Et_2_Ga(*μ*‐OTeF_5_)_2_Ga(Et)(OTeF_5_).^[17]^ Further investigations by IR and Raman vibrational spectroscopy confirm the presence of the dimeric [Al(OTeF_5_)_2_Me]_2_. More details can be found in the Supporting Information.

To obtain the fully teflate‐substituted Lewis acid [Al(OTeF_5_)_3_]_2,_ either starting from AlMe_3_ and HOTeF_5_ or [Al(OTeF_5_)_2_Me]_2_, a slight excess of teflic acid and further heating to room‐temperature is needed. Removing the solvent at reduced pressure again leads to the isolation of a colorless powder. The comparison of recorded IR and Raman spectra of [Al(OTeF_5_)_3_]_2_ to [Al(OTeF_5_)_2_Me]_2_ shows the absence of residual methyl groups (cf. spectra in Supporting Information). Furthermore, this product is stable for several hours at room temperature and can be stored at −20 °C under an argon atmosphere for months without any decomposition.

### Solvent adducts

**Figure 3:**
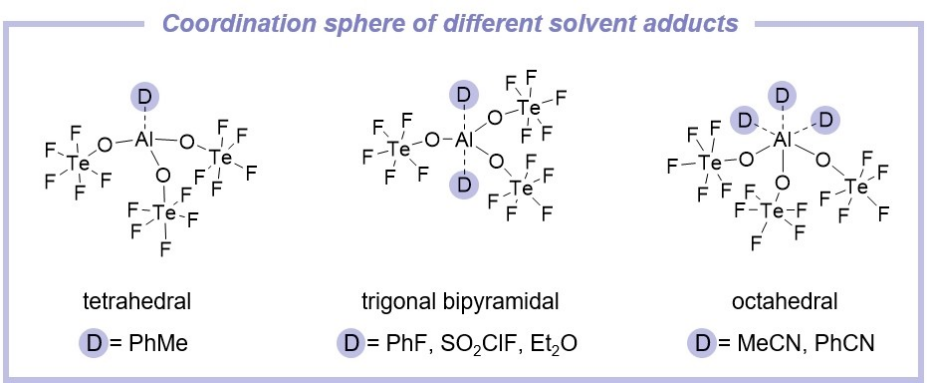
Summary of structurally characterized solvent adducts of the Lewis acid Al(OTeF_5_)_3_.

The reactivity and acidity of the Lewis acid [Al(OTeF_5_)_3_]_2_ in further reactions is clearly dependent on the solvent that is used. In the following section the interaction of the dimer [Al(OTeF_5_)_3_]_2_ with different solvents is described. While either no solubility at low temperatures or decomposition was observed with non‐polar solvents such as alkanes and methylene chloride, a number of different solvent adducts are obtained with stronger donors (cf. Figure 3).

**Scheme 2:**
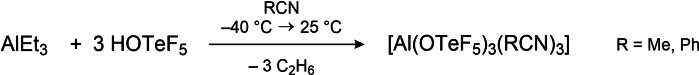
Synthesis of nitrile solvent‐adducts [Al(OTeF_5_)_3_(RCN)_3_].

In our previous work, we briefly discussed the synthesis of the solvent‐adduct [Al(OTeF_5_)_3_(MeCN)] by the reaction of HOTeF_5_ with AlEt_3_ in n‐pentane and the subsequent addition of equimolar amounts of MeCN.^[14]^ For this work we extended the range of nitriles and used acetonitrile as well as benzonitrile (PhCN) as solvent (cf. Scheme 2) and further analyzed the formed compounds by NMR and, in the case of the benzonitrile adduct, also by SC‐XRD. In both cases the removal of all volatiles yields a room‐temperature stable colorless powder in almost quantitative yields, which can be stored under an argon‐atmosphere at room temperature for several months without any sign of decomposition.

**Scheme 3:**
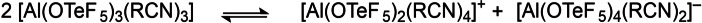
Equilibrium between the neutral nitrile adduct of Al(OTeF_5_)_3_ and its autoionized ion‐pair. (R=Me, Ph).

NMR‐spectroscopic investigation of the nitrile adducts reveal an octahedral coordination sphere at the Al center in solution, by showing very broad signals in the typical range of octahedrally coordinated aluminum centers between −10 and −25 ppm.^[18]^ This indicates the formation of [Al(OTeF_5_)_3_(MeCN)_3_] and [Al(OTeF_5_)_3_(PhCN)_3_], which is further supported by crystallographic evidence in the case of PhCN (see below). The ^19^F NMR spectra intriguingly show three sets of AB_4_ spin systems, indicating three magnetically inequivalent −OTeF_5_ groups. This is explained by an equilibrium of the neutral solvent adduct [Al(OTeF_5_)_3_(RCN)_3_] (R=MeCN or PhCN) and an autoionized ion‐pair of the type [Al(OTeF_5_)_2_(RCN)_4_][Al(OTeF_5_)_4_(RCN)_2_] (cf. Scheme 3). Analogous autoionization products are known for other aluminum halides, such as AlBr_3_ and AlCl_3_, in tetrahydrofurane.^[19]^

Dissolution of [Al(OTeF_5_)_3_(RCN)_3_] in less nucleophilic solvents like CH_2_Cl_2_ or *o*‐DFB leads to a complete autoionization and the formation of [AI(OTeF_5_)_4_]^−^ next to [Al(OTeF_5_)_2_(RCN)_4_]^+^ and [Al(OTeF_5_)_4_(RCN)_2_]^−^. In addition, the exchange of all −OTeF_5_ groups is observed in the corresponding ^19^F,^19^F EXSY NMR measurement (see Supporting Information for more details).

**Figure 4:**
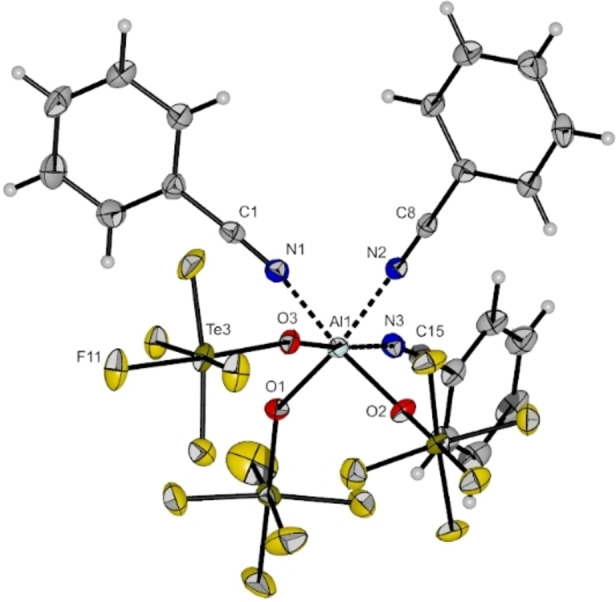
Molecular structure of [Al(OTeF_5_)_3_(PhCN)_3_] in the solid state. Thermal ellipsoids set up to 50 % probability. Selected bond lengths [pm] and angles [°]: Al1‐O1 182.5(3), Al1‐O2 183.4(3), Al1‐O3 183.2(3), Al1‐N1 203.7(3), Al1‐N2 200.3(3), Al1‐N3 203.6(3), O1‐Al1‐O2 98.34(13), O1‐Al1‐O3 93.94(13), O2‐Al1‐O3 96.04(12), N1‐Al1‐N2 84.62(13), N1‐Al1‐N3 87.38(13), N2‐Al1‐N3 88.95(13).

In the case of the benzonitrile adduct colorless single crystals suitable for SC‐XRD could be obtained. The compound [Al(OTeF_5_)_3_(PhCN)_3_] crystallizes in the monoclinic space group *P*2_1_/*c* with three molecules per asymmetric unit (cf. Figure 4). The octahedral complex possesses a facial coordination sphere. The Al−O bond lengths lie between 181.4(3) and 183.4(3) pm and are shorter than the Al−O bond distances of the two other known six‐fold coordinated aluminum pentafluoro‐orthotellurates [Li(thf)_4_][Al(OTeF_5_)_4_(thf)_2_] and [Ag(thf)_6_][Al(OTeF_5_)_4_(thf)_2_].^[20]^ The Al−N bond distances range from 200.3(3) to 203.7(3) pm. All of the O−Al−O bond angles are with an average of 95.8° larger than the N−Al−N bond angles with an average of 86.7°, leading to a slight distortion of the coordination sphere.

**Figure 5:**
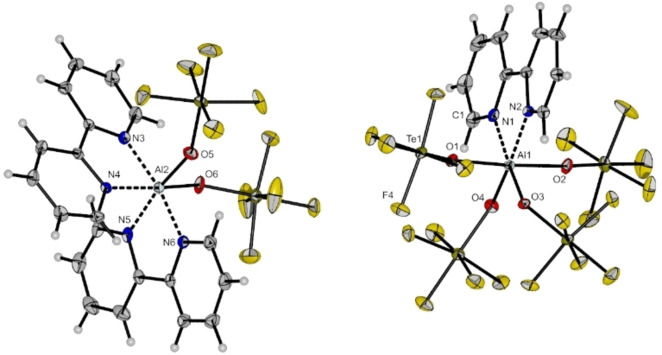
Molecular structure of [Al(OTeF_5_)_2_(bipy)_2_][Al(OTeF_5_)_4_(bipy)] in the solid state. Thermal ellipsoids set up to 50 % probability. Al1‐O1 188.0(2), Al1‐O2 187.6(3), Al1‐O3 183.9(2), Al1‐O4 184.9(2), Al1‐N1 204.6(3), Al1‐N2 205.7(3), Al2‐O5 183.1(3), Al2‐O6 183.6(3), Al2‐N3 200.8(3), Al2‐N4 204.2(3), Al2‐N5 203.5(3), Al2‐N6 200.7(3), O1‐Al1‐O2 176.22(12), N1‐Al1‐O3 171.29(12), N2‐Al1‐O4 168.58(12), N3‐Al2‐N6 170.09(13), N4‐Al2‐O6 169.85(12), N5‐Al2‐O5 170.22(13).

To enable a structural characterization of the autoionized species, bipyridine (bipy) is added to a solution of [Al(OTeF_5_)_3_(PhCN)_3_] in CH_2_Cl_2_. A similar approach was recently reported by Gerken et al. for the autoionization of SbF_5_.^[21]^ The anticipated salt [Al(OTeF_5_)_2_(bipy)_2_][Al(OTeF_5_)_4_(bipy)] crystallizes in the triclinic space group *P* $\bar{1}$ (cf. Figure 5). The molecular structure shows two octahedrally coordinated aluminum complexes. The anionic fragment shows Al−O bond distances between 183.9(2) and 188.0(2) pm which are comparable to the reported Al−O bond distances in [Al(OTeF_5_)_4_(thf)_2_]^−^_._ ^[20]^ The Al−O bond distances of the cation are in a similar range and not shortened as one might expect. The same holds true for the Al−N bond distances in anion and cation.

**Scheme 4:**
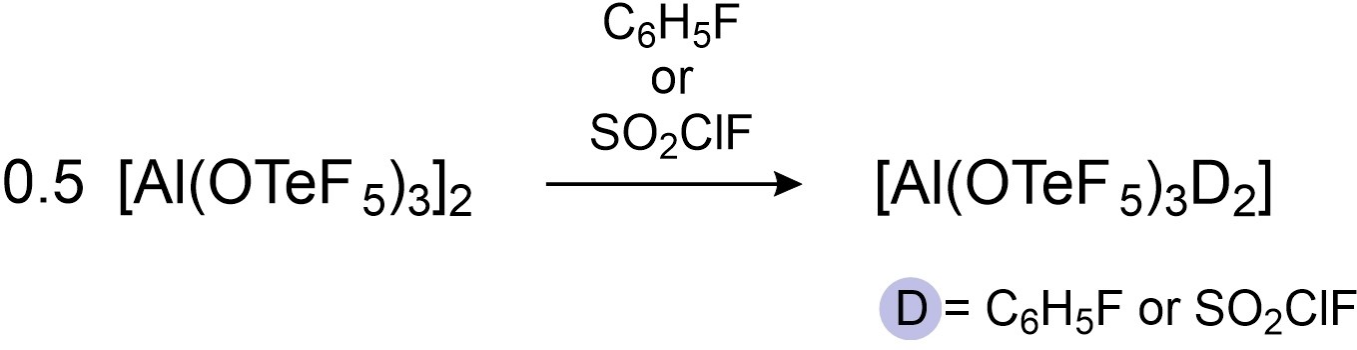
Neutral adduct formation with weakly donating solvents.

Compared to the neat Lewis acid [Al(OTeF_5_)_3_]_2_, the nitrile adducts form room‐temperature stable compounds and can be easily handled. The main drawback is the quenched Lewis acidity due to the coordination of the nitrile molecules. In order to preserve a high reactivity of the underlying Lewis acid we aimed to prepare adducts with weaker donor solvents. Therefore, the solid dimer [Al(OTeF_5_)_3_]_2_ was dissolved in fluorobenzene and SO_2_ClF, respectively (cf. Scheme 4).

Dissolution of [Al(OTeF_5_)_3_]_2_ in fluorobenzene at 0 °C results in a light green, clear solution. Warming the mixture to room‐temperature leads to visible decomposition of the compound. Low‐temperature NMR spectroscopic measurements revealed the presence of a single aluminum species in the ^27^Al NMR spectrum. The broad signal at −46.1 ppm is in the typical range for tetrahedrally coordinated Al centers and in agreement with the chemical shift of the literature‐known [Al{OC(CF_3_)_3_}_3_(PhF)], thus pointing to the formation of [Al(OTeF_5_)_3_(PhF)].^[5]^ The ^19^F NMR spectrum shows signals corresponding to one AB_4_ spin system with F_a_ at −40.7 ppm and F_b_ at −46.1 ppm with a coupling constant of ^2^ *J*_FF_=191 Hz. A signal for the coordinated PhF could not be observed in the ^19^F NMR spectrum since the spectra were recorded in fluorobenzene. Therefore, an exchange of the solvent molecules bound to the Al center can be expected. Attempts to isolate the compound as a neat substance did not yield in any success.

**Figure 6:**
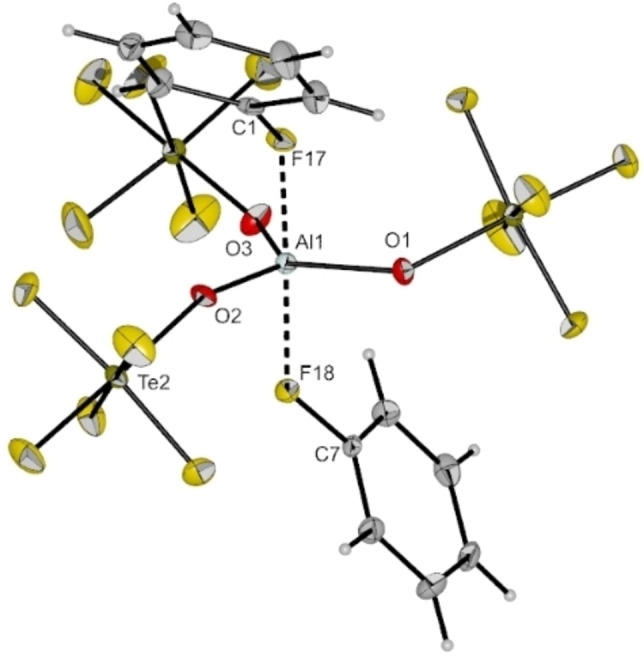
Molecular structure of [Al(OTeF_5_)_3_(PhF)_2_] in the solid state. Thermal ellipsoids set up to 50 % probability. Selected bond lengths [pm] and angles [°]: Al1‐O1 172.7(2), Al1‐O2 172.4(2), Al1‐O3 173.1(2), Al1‐F17 197.9(2), Al1‐F18 197.4(2), C1‐F17 142.4(3), C7‐F18 142.4(3), O1‐Al1‐O2 123.41(11), O1‐Al1‐O3 115.04(11), O2‐Al1‐O3 121.56(11), C7‐F18‐Al1 135.03(16), C1‐F17‐Al1 134.03(16).

Concentration of a solution of [Al(OTeF_5_)_3_]_2_ in PhF and further cooling to −40 °C resulted in yellow‐green single crystals which were examined by single crystal X‐ray diffraction. Instead of a tetrahedral aluminum complex, the five‐fold coordinated complex [Al(OTeF_5_)_3_(PhF)_2_] was found (cf. Figure 6). The compound crystallizes in the monoclinic space group *P*2_1_/*n*. In this structure the aluminum center has a trigonal‐bipyramidal coordination sphere with three −OTeF_5_ groups in the equatorial plane and two fluorobenzene molecules in the axial positions. The Al−O bond lengths range from 172.7(2) to 173.1(2) pm and the bond angles between the Al−O bonds lie between 115.04(11)° and 123.41(11)°. The fluorobenzene molecules coordinate the aluminum center via their fluorine atom. With bond lengths of *d*(Al1‐F17)=197.9(2) pm and *d*(Al1‐F18)=197.4(2) pm, the Al−F bonds are elongated compared to the ones reported for the tetrahedral Lewis superacid adduct [Al{OC(CF_3_)_3_}_3_(PhF)] (*d*(Al−F)=186.4(2) pm).^[5]^ Analogous to the findings of Krossing et al.,^[5]^ the C−F bond lengths of the bound fluorobenzene molecules are elongated by about 7 pm compared to neat fluorobenzene.^[22]^

Changing the solvent and treating solid [Al(OTeF_5_)_3_]_2_ with an excess of SO_2_ClF results in a clear colorless solution. In contrast to the experiments with fluorobenzene, this mixture is sufficiently stable at room temperature. Interestingly, the adduct formation with SO_2_ClF yields the trigonal‐bipyramidal complex [Al(OTeF_5_)_3_(SO_2_ClF)_2_] in solution and in the solid state. The ^27^Al NMR spectrum shows a very broad signal at 34.0 ppm (FWHM=2200 Hz), which lies between the typical regions of four‐fold and six‐fold coordinated Al centers. Similar to the experiments with PhF, the ^19^F NMR spectrum shows only one AB_4_ spin system belonging to the three magnetically equivalent −OTeF_5_ groups.

A colorless powder is isolated by removing all volatiles at reduced pressure. This powder is stable for several hours at room temperature and was analyzed by IR and Raman spectroscopy. Besides the typical bands of Al(OTeF_5_)_3_ in the IR spectrum, additional bands at 1436 (Raman: 1428) and 1188 (Raman: 1182) cm^−1^ for the SO_2_ stretching vibrations of the coordinated SO_2_ClF molecules are observed. Compared to free SO_2_ClF (*ν*_as_(SO_2_)=1455 cm^−1^, *ν*_s_(SO_2_)=1224 cm^−1^) these bands are slightly red‐shifted, which indicates a coordination of the SO_2_ClF molecules via the oxygen atom.^[23]^

**Figure 7:**
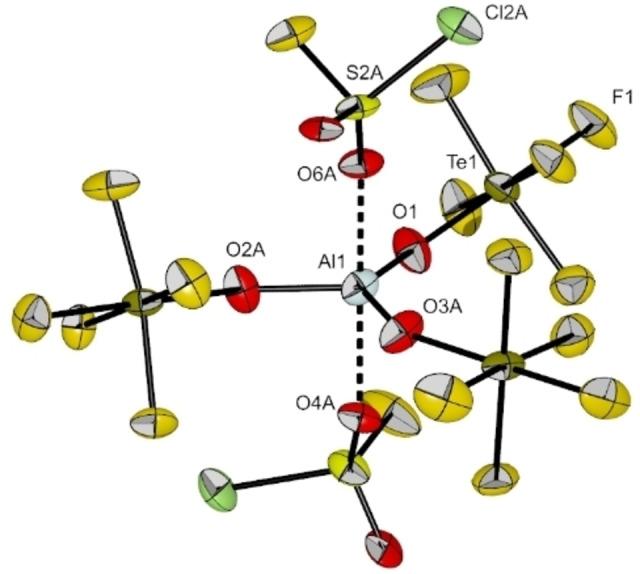
Molecular structure of [Al(OTeF_5_)_3_(SO_2_ClF)_2_] in the solid state. Thermal ellipsoids set up to 50 % probability. Selected bond lengths [pm] and angles [°]: Al1‐O1 172.0(3), Al1‐O2 A 177.9(6), Al1‐O3 A 173.4(4), Al1‐O4 A 210.4(6), Al1‐O6 A 198.9(6), O1‐Al1‐O2 A 114.76(19), O1‐Al1‐O3 A 131.9(2), O2 A‐Al1‐O3 A 113.02(6).

After concentrating the solution of [Al(OTeF_5_)_3_(SO_2_ClF)_2_] and slowly cooling it to −80 °C single crystals suitable for single crystal X‐ray diffraction were obtained. The compound [Al(OTeF_5_)_3_(SO_2_ClF)_2_] crystallizes in the triclinic space group *P* $\bar{1}$ (cf. Figure 7). Analogous to the molecular structure of [Al(OTeF_5_)_3_(PhF)_2_], the −OTeF_5_ groups build the equatorial plane and the SO_2_ClF molecules are bound in axial position to the central aluminum atom, resulting in a trigonal bipyramidal coordination sphere. For the Al−O bonds between aluminum and the teflate groups an average bond distance of 174.3 pm with average bond angles of 119.9° is found, which is comparable to the distances and angles in [Al(OTeF_5_)_3_(PhF)_2_]. As already discovered by vibrational spectroscopy, the SO_2_ClF molecules are bound by their oxygen atoms with Al−O bond lengths of 210.4(6) and 198.9(6) pm. To the best of our knowledge, only one other example of a molecular structure with an oxygen‐bound SO_2_ClF molecule has been reported so far, which is [Xe(OTeF_5_)(SO_2_ClF)][Sb(OTeF_5_)_6_]. Here, the SO_2_ClF molecule is coordinated to the [Xe(OTeF_5_)]^+^ cation.^[24]^

A remarkable example for a weakly coordinated aluminum complex was shown by Cowley, Jones, and coworkers, when they first synthesized the arene complexes [Al(C_6_F_5_)_3_(*η* ^1^‐C_6_H_6_)] and [Al(C_6_F_5_)_3_(*η* ^1^‐C_7_H_8_)] starting from AlMe_3_ and B(C_6_F_5_)_3_ in benzene or toluene.^[25]^ Own attempts to form an arene adduct in an analoguous route with B(OTeF_5_)_3_ as −OTeF_5_ group transfer reagent were unsuccessful. Also dissolving the solid [Al(OTeF_5_)_3_]_2_ in toluene led to the decomposition of the Lewis acid. The failed reaction can be explained by the low binding energy of a toluene complex compared to the dimeric species and will be discussed in a later section.

**Scheme 5:**
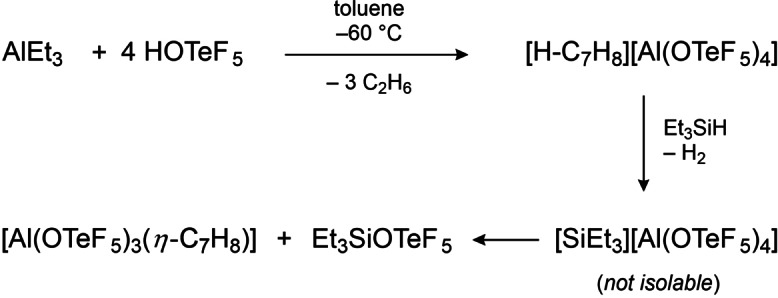
Proposed mechanism for the formation of the toluene adduct [Al(OTeF_5_)_3_(*η* ^1^‐C_7_H_8_)].

Nevertheless, it was possible to obtain an arene adduct by a detour (cf. Scheme 5). In the first step, a solution of AlEt_3_ in toluene is treated with 4 equivalents of HOTeF_5_, which leads to the protonation of toluene, thereby forming the strong Brønsted acid [H‐C_7_H_8_][Al(OTeF_5_)_4_]. Similar procedures are already described in the literature.[^14^, ^26^] In the next step, a slight excess of triethylsilane, Et_3_SiH, is added to the mixture, followed by a gas formation accompanied by the decolorization of the bright orange solution. Upon addition of Et_3_SiH, presumably the cationic silylium species [SiEt_3_]^+^ is formed alongside the evolution of gaseous H_2_, which is a sufficiently strong electrophile to abstract an −OTeF_5_ group from the anion [Al(OTeF_5_)_4_]^−^. Thereby, Et_3_SiOTeF_5_ and the Lewis acid Al(OTeF_5_)_3_ are formed, the latter of which is stabilized by the present toluene. Warming the reaction mixture above −40 °C results in the visible decomposition of the sample. Therefore, the reaction solution was analyzed by low‐temperature NMR spectroscopy. The ^19^F NMR spectrum reveals two magnetically inequivalent −OTeF_5_ groups, assigned to the formed Et_3_SiOTeF_5_ and the solvent‐adduct [Al(OTeF_5_)_3_(*η* ^1^‐C_7_H_8_)]. In the ^27^Al NMR spectrum a broad signal at 48 ppm for [Al(OTeF_5_)_3_(*η* ^1^‐C_7_H_8_)] is observed. The ^1^H and ^29^Si NMR spectra confirm the presence of Et_3_SiOTeF_5_ and residual Et_3_SiH.

**Figure 8:**
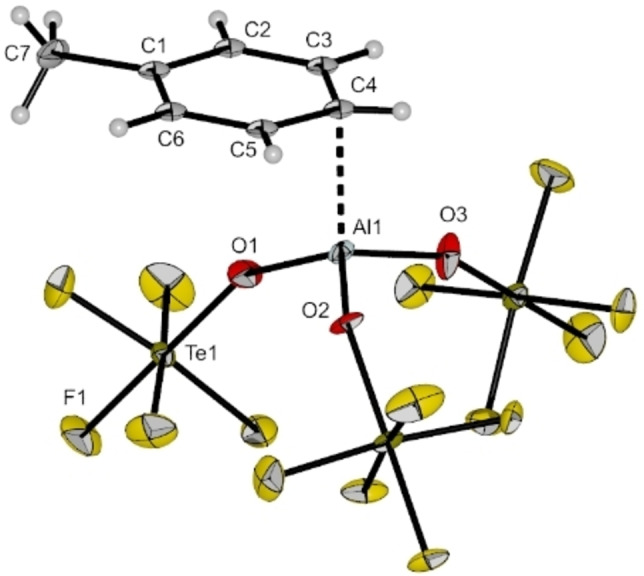
Molecular structure of [Al(OTeF_5_)_3_(*η* ^1^‐C_7_H_8_)] in the solid state. Thermal ellipsoids set up to 50 % probability. Selected bond lengths [pm] and angles [°]: Al1‐O1 171.3(7), Al1‐O2 173.4(7), Al1‐O3 172.6(8), Al1‐C4 214.3(10), O1‐Al1‐O2 114.2(4), O1‐Al1‐O3 112.9(4), O1‐Al1‐C4 111.1(4), O2‐Al1‐O3 110.4(4), O2‐Al1‐C4 105.1(4), O3‐Al1‐C4 102.3(4), Al1‐C4‐C5 91.7(6), Al1‐C4‐C3 95.8(6).

Further cooling of the reaction mixture to −80 °C led to colorless crystals suitable for single crystal X‐ray diffraction. The compound [Al(OTeF_5_)_3_(*η* ^1^‐C_7_H_8_)]⋅C_7_H_8_ crystallizes in the monoclinic space group *P*2_1_/*c* (cf. Figure 8). The aluminum center is distorted tetrahedrally coordinated by three −OTeF_5_ ligands and a toluene molecule via its *para*‐carbon atom. The Al−O bond lengths are in the same range as the aforementioned solvent adducts [Al(OTeF_5_)_3_(SO_2_ClF)_2_] and [Al(OTeF_5_)_3_(PhF)_2_]. The average Al−O bond angle of 112.5° is close to the ideal tetrahedral angle. The Al1‐C4 bond distance of 214.3(10) pm is significantly shorter than in the complexes [Al(C_6_F_5_)_3_(*η* ^1^‐C_6_H_6_)] and [Al(C_6_F_5_)_3_(*η* ^1^‐C_7_H_8_)] (*d*(Al‐C_toluene_)=236.6(2) pm and *d*(Al‐C_benzene_)=234.2(6) pm).^[25]^ An analoguous coordination motif was reported by Lambert et al. in the molecular structure of [Et_3_Si(*η* ^1^‐C_7_H_8_)][B(C_6_F_5_)_4_], whereby the highly Lewis acidic silylium ion is *η* ^1^‐coordinated by toluene (*d*(Si‐C_toluene_)=218 pm).^[27]^ The geometry at the C4 atom indicates a *sp* ^2^ hybridization (∠(C3‐C4‐C5)=119.4(10)°) and the donor‐acceptor bond between Al and C is therefore best described as a π‐arene complex (∠(Al1‐C4‐C3)=95.8(6)° and ∠(Al1‐C4‐C5)=91.7(6)°). This is further supported by the maintained planarity of the toluene molecule (largest torsion angle: 3.9°) and similar aromatic C−C bond lengths (ranging between 137.6(14) and 141.2(14) pm). The contrary case of a Wheland‐type σ‐complex would require a *sp* ^3^ hybridized carbon atom C4 with bond angles close to 109°, alternating C−C bond lengths and a loss of planarity of the aromatic ring.

In order to estimate the ligand affinity of the Lewis acid Al(OTeF_5_)_3_ towards the different solvents and judge the remaining Lewis acidity of the solvent adducts, the fluoride ion affinities and the complexation energies of the adducts have been calculated on the BP86/def‐SV(P) and B3LYP/def2‐TZVPP level of theory. The results are summarized in Table 1. For the discussion of the calculated FIA values, the BP86/def‐SV(P) level of theory and the isodesmic reactions with trimethylsilane as anchor are used to allow a comparison with a previously reported FIA calculation.[^2^, ^28^]

**Table 1:** Calculated fluoride ion affinities (FIA) and complexation energies of various solvent adducts of Al(OTeF_5_)_3_.

compound	FIA [kJ mol^−1^]		reaction			Δ_R_ *H*° [kJ mol^−1^]	
	BP86/def‐SV(P)	B3LYP/def2‐TZVPP				BP86/def‐SV(P)	B3LYP/def2‐TZVPP
Al(OTeF_5_)_3_	591	637		–		–	–
[Al(OTeF_5_)_3_]_2_	569	584	2 Al(OTeF_5_)_3_	→	[Al(OTeF_5_)_3_]_2_	−21.9	−53.6

tetrahedral coordination							
[Al(OTeF_5_)_3_(*η* ^1^‐C_7_H_8_)]	566	590	Al(OTeF_5_)_3_+C_7_H_9_	→	[Al(OTeF_5_)_3_(*η* ^1^‐C_7_H_8_)]	−25.1	−47.1
[Al(OTeF_5_)_3_(SO_2_ClF)]	584	580	Al(OTeF_5_)_3_+SO_2_ClF	→	[Al(OTeF_5_)_3_(SO_2_ClF)]	−7.7^[a]^	−57.0
[Al(OTeF_5_)_3_(PhF)]	581	581	Al(OTeF_5_)_3_+PhF	→	[Al(OTeF_5_)_3_(PhF)]	−9.3^[a]^	−56.0
[Al(OTeF_5_)_3_(MeCN)]	493	499	Al(OTeF_5_)_3_+MeCN	→	[Al(OTeF_5_)_3_(MeCN)]	−97.9	−138.3
[Al(OTeF_5_)_3_(PhCN)]	481	489	Al(OTeF_5_)_3_+PhCN	→	[Al(OTeF_5_)_3_(PhCN)]	−110.1	−148.7
[Al(OTeF_5_)_3_(Et_2_O)]	477	493	Al(OTeF_5_)_3_+Et_2_O	→	[Al(OTeF_5_)_3_(Et_2_O)]	−114.6	−144.7

trigonal bipyramidal coordination							
[Al(OTeF_5_)_3_(SO_2_ClF)_2_]	555	571	Al(OTeF_5_)_3_+2 SO_2_ClF	→	[Al(OTeF_5_)_3_(SO_2_ClF)_2_]	−35.9	−66.6
[Al(OTeF_5_)_3_(PhF)_2_]	540	564	Al(OTeF_5_)_3_+2 PhF	→	[Al(OTeF_5_)_3_(PhF)_2_]	−50.9	−73.2
[Al(OTeF_5_)_3_(OEt_2_)_2_]	408	474	Al(OTeF_5_)_3_+2 Et_2_O	→	[Al(OTeF_5_)_3_(Et_2_O)_2_]	−183.0	−163.4

octahedral coordination							
[Al(OTeF_5_)_3_(MeCN)_3_]	368	441	Al(OTeF_5_)_3_+3 MeCN	→	[Al(OTeF_5_)_3_(MeCN)_3_]	−223.0	−196.5
[Al(OTeF_5_)_3_(PhCN)_3_]	348	435	Al(OTeF_5_)_3_+3 PhCN	→	[Al(OTeF_5_)_3_(PhCN)_3_]	−243.1	−202.6

[a] Those values seem unreliable due to basis set limitation. The calculations were repeated with a larger basis set (BP86/def‐TZVP) and are found to give a more consistent value: Δ_R_ *H*°(Al(OTeF_5_)_3_+SO_2_ClF→[Al(OTeF_5_)_3_(SO_2_ClF)])=−21.3 kJ mol^−1^_;_ Δ_R_ *H*°(Al(OTeF_5_)_3_+PhF→[Al(OTeF_5_)_3_(PhF)])=−26.7 kJ mol^−1^.

In general, by expanding the ligand coordination sphere of the aluminum center starting from a tetrahedral over a trigonal bipyramidal to an octahedral coordination, a decrease of the Lewis acidity is observed. For the tetrahedrally coordinated complexes, the Lewis acidity of the Al center decreases in the order toluene>SO_2_ClF>PhF>MeCN>PhCN>Et_2_O. This is also reflected in the experiment, whereby the complex stability increases in the same order. This trend is also in agreement with experimental donor numbers of the respective solvents.^[29]^ The calculated FIA values of the experimentally observed complexes [Al(OTeF_5_)_3_(PhF)_2_], [Al(OTeF_5_)_3_(SO_2_ClF)_2_] and [Al(OTeF_5_)_3_(*η* ^1^‐C_7_H_8_)] still surpass the fluoride ion affinity of molecular SbF_5_ (FIA: 493 kJ mol^−1^) and therefore a high reactivity can be expected. The calculations also reveal significantly lower fluoride ion affinities of the nitrile adducts [Al(OTeF_5_)_3_(MeCN)_3_] and [Al(OTeF_5_)_3_(PhCN)_3_] than SbF_5_. Regarding the calculated complexation enthalpies Δ_R_ *H*°, the results based on the calculations with B3LYP/def2‐TZVPP level of theory are discussed and the following trend is observed: If the FIA value of a Lewis acid‐solvent adduct is low, the corresponding complexation reaction is highly exothermic. The Δ_R_ *H*° values also allow to estimate whether a complexation of [Al(OTeF_5_)_3_]_2_ with a solvent is possible. Only in the case of [Al(OTeF_5_)_3_(*η* ^1^‐C_7_H_8_)] the dimerization is thermodynamically favored compared to the toluene‐adduct formation (Δ_R_ *H*°([Al(OTeF_5_)_3_]_2_)=−53.6 kJ mol^−1^, Δ_R_ *H*°([ Al(OTeF_5_)_3_(*η* ^1^‐C_7_H_8_)])=−47.1 kJ mol^−1^). This explains why the toluene adduct is not accessible directly by dissolving dimeric [Al(OTeF_5_)_3_]_2_ in toluene.

### Reactivity of solvent adducts

**Scheme 6:**
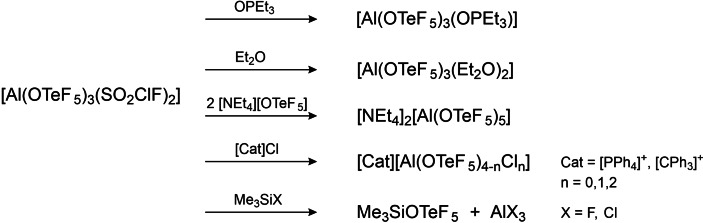
Reactions of solvent adducts of Al(OTeF_5_)_3_ with different substrates.

From the computational study we know that the adducts [Al(OTeF_5_)_3_(SO_2_ClF)_2_] and [Al(OTeF_5_)_3_(PhF)_2_] still remain very strong Lewis acids. Therefore, they were treated with different reactants, including stronger Lewis bases such as the phosphine oxide OPEt_3_ and diethyl ether. Additionally, reactions with chloride sources were conducted, including the salt [PPh_4_]Cl and trityl chloride, CPh_3_Cl (cf. Scheme 6).

**Figure 9:**
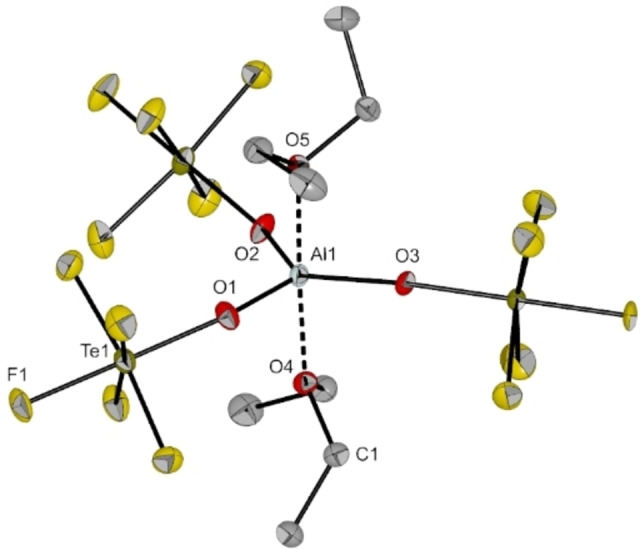
Molecular structure of [Al(OTeF_5_)_3_(Et_2_O)_2_] in the solid state. Thermal ellipsoids set up to 50 % probability. Selected bond lengths [pm] and angles [°]: Al1‐O1 176.0(2), Al1‐O2 176.4(2), Al1‐O3 174.8(2), Al1‐O4 195.7(2), Al1‐O5 197.1(3), O1‐Al1‐O2 119.82(12), O1‐Al1‐O3 116.90(13), O2‐Al1‐O3 123.27(13).

In some cases, it was not possible to successfully form a solvent‐adduct by just adding an excess of solvent to the neat Lewis acid [Al(OTeF_5_)_3_]_2_. This problem could be circumvented by starting from weakly bound adducts. As an example, we added a small excess of diethyl ether to [Al(OTeF_5_)_3_(SO_2_ClF)_2_] in SO_2_ClF, resulting in the formation of the diethyl ether adduct. By slowly cooling the reaction mixture to −80 °C it was possible to obtain colorless crystals of the product. The compound [Al(OTeF_5_)_3_(Et_2_O)_2_] crystallizes in the triclinic space group *P* $\bar{1}$ (cf. Figure 9). Similar to the adducts with PhF and SO_2_ClF, the complex possesses a trigonal bipyramidal coordination sphere at the Al center. The bond distances between the aluminum and the oxygen atom of Et_2_O (d(Al1‐O4)=195.7(2) and d(Al1‐O5)=197.1(3) pm) are comparable to the analogue bond lengths in literature‐known [Al(OC_5_F_4_N)_3_(Et_2_O)_2_].^[11]^ This reflects well on the similar Lewis acidity of the two compounds.

An established method for experimentally gauging the acidity of a Lewis acid is the Gutmann‐Beckett method, in which the ^31^P NMR chemical shift of triethylphosphine oxide, OPEt_3_, in a Lewis acid complex is analyzed in respect to free OPEt_3_.^[30]^ By reacting an equimolar amount of OPEt_3_ with [Al(OTeF_5_)_3_]_2_ in SO_2_ClF it was possible to obtain the tetrahedral complex [Al(OTeF_5_)_3_OPEt_3_]. Note that any excess of the phosphine will lead to multiple coordination to the Al center and therefore result in ambiguous signals in the corresponding NMR spectra. The ^31^P NMR of this compound gave a signal at 83.9 ppm. Compared to free OPEt_3_ (*δ* in CD_2_Cl_2_: 50 ppm) this resonance is shifted by 33.9 ppm and clearly surpasses other aluminum based Lewis superacids such as Al(C_6_F_5_)_3_ (Δ*δ*: 26.0 ppm)^[31]^ and Al[OC(C_6_F_5_)_3_]_3_ (Δ*δ*: 23.9 ppm).^[12]^ This high value is also in line with the calculated FIA of Al(OTeF_5_)_3_. Therefore, this compound combines both, a high global (according to FIA) and effective (according to GB method) Lewis acidity.^[32]^

**Figure 10:**
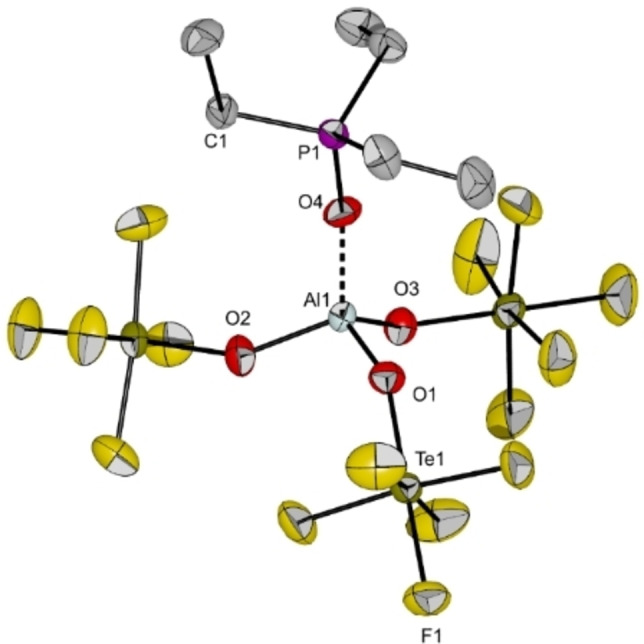
Molecular structure of [Al(OTeF_5_)_3_(OPEt_3_)] in the solid state. Thermal ellipsoids set up to 50 % probability. Selected bond length [pm] and angles [°]: Al1‐O1 174.1(3), Al1‐O2 173.3(3), Al1‐O3 173.6(3), Al1‐O4 171.3(3), P1‐O4 153.2(3), O1‐Al1‐O2 108.16(14), O1‐Al1‐O3 111.56(13), O1‐Al1‐O4 106.78(14), O2‐Al1‐O3 106.97(15), O2‐Al1‐O4 113.13(15), O3‐Al1‐O4 110.28(14), Al1‐O4‐P1 159.9(2).

By cooling a concentrated solution of [Al(OTeF_5_)_3_OPEt_3_] in SO_2_ClF colorless crystals were obtained. The compound [Al(OTeF_5_)_3_OPEt_3_] crystallizes in the orthorhombic space group *Pbca* (cf. Figure 10). The complex has a distorted tetrahedral coordination sphere and the Al−O bond lengths of the pentafluoroorthotellurate groups range between 173.3(3) and 174.1(3) pm and are comparable to the Al−O bonds in the tetrahedral complex [Al(OTeF_5_)_3_(*η* ^1^‐C_7_H_8_)]. Interestingly, the Al−O bond length of the OPEt_3_ moiety is the shortest with 171.3(3) pm, which indicates a strong interaction of the Lewis basic OPEt_3_ with the Al center.

To further validate the high fluoride ion affinity of these solvent adducts, [Al(OTeF_5_)_3_(SO_2_ClF)_2_] was reacted with the salt [PPh_4_][SbF_6_] in SO_2_ClF aiming to abstract one fluoride atom of the anion [SbF_6_]^−^. Upon addition of the phosphonium salt, the precipitation of a brown solid was observed. Analysis by NMR spectroscopy reveals the formation of the salt [PPh_4_][Al(OTeF_5_)_4_] and small amounts of HOTeF_5_. This outcome can be rationalized by the successful initial abstraction of a fluoride from [SbF_6_]^−^, followed by ligand scrambling which leads to the formation of the anion [Al(OTeF_5_)_4_]^−^ and precipitation of presumably insoluble AlF_3_. Additionally, side reactions of SbF_5_ with the cation led to the formation of HF which then further reacts with [Al(OTeF_5_)_4_]^−^ to HOTeF_5_. This explains why no signals of SbF_5_ could be detected in the ^19^F NMR spectrum.

By adding equimolar amounts of [NEt_4_]OTeF_5_ to a solution of [Al(OTeF_5_)_3_(SO_2_ClF)_2_] in SO_2_ClF it is possible to form the already known anion [Al(OTeF_5_)_4_]^−^. Interestingly, increasing the amount of [NEt_4_][OTeF_5_] leads to a further coordination of OTeF_5_ groups to the central Al atom. Therefore, by adding a two‐fold excess of the ammonium salt gave the di‐anion [Al(OTeF_5_)_5_]^2−^, which could be analyzed by NMR and vibrational spectroscopy. The solubility of this compound is very limited. Furthermore, we succeeded in the crystallization of [NEt_4_]_2_[Al(OTeF_5_)_5_] by forming this species on an alternative route (see Supporting Information).

**Figure 11:**
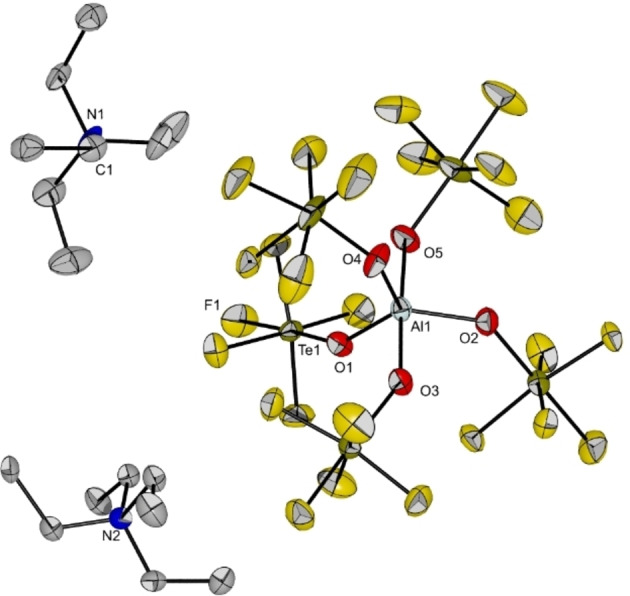
Molecular structure of [NEt_4_]_2_[Al(OTeF_5_)_5_] in the solid state. Thermal ellipsoids set up to 50 % probability. Selected bond length [pm] and angles [°]: Al1‐O1 178.6(4), Al1‐O2 180.4(4), Al1‐O3 186.7(4), Al1‐O4 179.7(4), Al1‐O5 184.6(4), O1‐Al1‐O2 125.3(2), O1‐Al1‐O4 118.6(2), O2‐Al1‐O4 115.9(2), O3‐Al1‐O5 176.7(2).

The compound [NEt_4_]_2_[Al(OTeF_5_)_5_] crystallizes in the monoclinic spacegroup *P*2_1_/*c* (cf. Figure 11). The anion possesses a distorted trigonal bipyramidal coordination sphere at the Al center. The average Al−O bond distances of the axial teflate ligands are with 185.7 ppm slightly elongated when compared to the equatorial Al−O distances (average *d*(Al−O): 179.6 pm).

Addition of the phosphonium salt [PPh_4_]Cl to a solution of [Al(OTeF_5_)_3_(PhF)_2_] in fluorobenzene led to the formation of the mixed aluminate salts [Al(OTeF_5_)_4–*n*_Cl_*n*_]^−^ (*n*=0,1,2,3). The ^27^Al NMR spectrum of this reaction shows three sharp signals at 80.5, 65.1, and 47.6 ppm, corresponding to the [Al(OTeF_5_)_2_Cl_2_]^−^, [Al(OTeF_5_)_3_Cl]^−^, and [Al(OTeF_5_)_4_]^−^ ions.^[14]^ These resonances are flanked by ^125^Te satellites of the corresponding isotopologues. In the ^19^F NMR spectrum three AB_4_ patterns are observed, which are assigned to the chloroaluminates by their integral ratio.

Treating the adduct [Al(OTeF_5_)_3_(SO_2_ClF)_2_] with trityl chloride CPh_3_Cl in SO_2_ClF immediately yields an intense yellow solution, already indicating the formation of the carbocation [CPh_3_]^+^. Analysis by NMR spectroscopy shows beside the formation of the desired cation again a mixture of anions [Al(OTeF_5_)_4‐*n*_Cl_*n*_]^−^ (*n*=0,1,2,3) as mentioned above (more details in Supporting Information). A similar ligand scrambling of the anion was reported by Riddlestone et al. when they treated the Lewis acid Al(OC_5_F_4_N)_3_ with trityl chloride.^[11]^

In an attempt to obtain halogen‐bridged adducts of the form [Al(OTeF_5_)_3_(Me_3_SiF)] and [Al(OTeF_5_)_3_(Me_3_SiCl)]^[33]^ in analogy to the literature‐known [Al{OC(CF_3_)_3_}_3_(Me_3_SiF)] and [Al{OC(CF_3_}_3_)_3_(Me_3_SiCl)],^[15]^ we treated [Al(OTeF_5_)_3_(SO_2_ClF)_2_] with the respective trimethylsilyl halides. Instead of the desired reaction, the formation of the species Me_3_SiOTeF_5_ is observed in both cases by NMR spectroscopy.^[34]^ Moreover, in the reaction of [Al(OTeF_5_)_3_(SO_2_ClF)_2_] with Me_3_SiCl the formation of AlCl_3_ is observed in the ^27^Al NMR spectrum, while in the case of Me_3_SiF a colorless, insoluble solid precipitates, which is likely insoluble AlF_3_. Subsequently, a substitution of the −OTeF_5_ groups by the halogen atom of the trimethylsilyl halides takes place. This is due to two reasons: The high Lewis acidity and the steric accessibility of the Al atom in [Al(OTeF_5_)_3_(SO_2_ClF)_2_] allow a dynamic ligand exchange. Further, the formation of Me_3_SiOTeF_5_ and AlF_3_ are thermodynamically favored and therefore drive the reaction.

## Conclusion

In this work we report the improved synthesis of the Lewis superacid Al(OTeF_5_)_3_ in its neat dimeric form in gram‐scale, as well as the synthesis and characterization of a variety of solvent adducts. These range from octahedral complexes with strong donor molecules to extremely weakly bound tetrahedral complexes. Theoretical calculations on the complexation energies and fluoride ion affinities of these adducts show that, depending on the solvent, the reactivity of the Lewis acid can be controlled, while a very high acidity is retained. Experimental validation of its Lewis acidity by the Gutmann Beckett method and fluoride abstraction from a hexafluoroantimonate salt confirm the Lewis superacidity of Al(OTeF_5_)_3_. As expected, fluoride and chloride abstractions can be easily realized with this species, but the accessibility of the aluminum center can also lead to ligand scrambling. This allows Al(OTeF_5_)_3_ and its solvent‐adducts to be used in the future whenever an extreme high fluoride ion affinity is needed, a recent example being the successful synthesis of the perfluorinated trityl cation.^[35]^

## Experimental Section

All preparative work was carried out using standard Schlenk techniques. Glassware was greased with Triboflon III. The pentafluoroorthotelluric acid HOTeF_5_ was prepared as described elsewhere.^[36]^ All solid materials were handled inside a glove box with an atmosphere of dry argon (O2<0.5 ppm, H2O<0.5 ppm). All solvents were freshly dried with CaH_2_ before use and stored on molecular sieve. HSiEt_3_, FSiMe_3_ and ClSiMe_3_ were degassed prior to use and CPh_3_Cl was dried in dynamic vacuum overnight and stored in a dry argon box. Raman spectra were recorded on a Bruker MultiRAM II equipped with a low‐temperature Ge detector (1064 nm, 50–100 mW, resolution 4 cm^−1^). IR spectra were measured on a Bruker ALPHA FTIR spectrometer equipped with a diamond ATR attachment in a glove box filled with argon (resolution 4 cm^−1^). NMR spectra were recorded on a JEOL 400 MHz ECS or ECZ spectrometer. All reported chemical shifts were referenced to the Ξ values given in IUPAC recommendations of 2008 using the ^2^H signal of the deuterated solvent as internal reference.^[37]^ Chemical shifts and coupling constants of ^19^F NMR spectra are given as simulated by *gNMR*.^[38]^ Crystal data were collected with MoK_α_ radiation on a Bruker D8 Venture diffractometer with a CMOS area detector. Single crystals were picked at −40 °C under nitrogen atmosphere and mounted on a 0.15 mm Micromount using perfluoroether oil. The structures were solved with the *ShelXT* ^[39]^ structure solution program using intrinsic phasing and refined with the *ShelXL* ^[40]^ refinement package using least squares on weighted F2 values for all reflections using OLEX2.^[41]^ Deposition Number(s) 2165632 (for [Al(OTeF_5_)_2_Me]_2_), 2165805 (for [Al(OTeF_5_)_3_(PhCN)_3_]), 2165786 (for [Al(OTeF_5_)_2_(bipy)_2_]/[Al(OTeF_5_)_4_(bipy)]), 2161784 (for [Al(OTeF_5_)_3_(PhF)_2_]), 2161790 (for [Al(OTeF_5_)_3_(SO_2_ClF)_2_]), 2165797 (for [Al(OTeF_5_)_3_(*η* ^1^‐C_7_H_8_)]), 2165785 (for [Al(OTeF_5_)_3_(OEt_2_)_2_]), 2167629 (for [NEt_4_]_2_[Al(OTeF_5_)_5_]), 2170700 (for [Al(OTeF_5_)_3_(OPEt_3_)]) contain(s) the supplementary crystallographic data for this paper. These data are provided free of charge by the joint Cambridge Crystallographic Data Centre and Fachinformationszentrum Karlsruhe Access Structures service. The *Turbomole* program^[42]^ was used to perform calculations at the unrestricted Kohn‐Sham DFT level, using the BP86 or B3LYP hybrid functional^[43]^ (with RI^[44]^) in conjunction with basis sets def‐SV(P) and def2‐TZVPP.^[45]^ Minima on potential energy surfaces were characterized by normal mode analysis. Thermochemical data is provided without counterpoise correction but including zero‐point energy correction as obtained from harmonic vibrational frequencies.

**[AlMe(OTeF_5_)_2_]_2_**: Pentafluoroorthotelluric acid, HOTeF_5_, (726 mg, 3.00 mmol) was condensed onto a frozen solution of AlMe_3_ (72 mg, 1.00 mmol) in *n*‐pentane at −196 °C. After connection of a bubbler, the mixture was slowly warmed to −30 °C resulting in the formation of a colorless precipitate under gas evolution. Evaporation of all volatiles led to the isolation of a colorless powder (503 mg, 96 %). The solid compound can be stored at −30 °C without visible decomposition. Crystals suitable for SC‐XRD were grown by slowly cooling a concentrated solution in *ortho*‐difluorobenzene to −40 °C. ^1^H NMR (400 MHz, SO_2_ClF, ext. [D6]acetone, −40 °C): *δ*=−0.01 (s, Al‐CH_3_) ppm. ^19^F NMR (377 MHz, SO_2_ClF, ext. [D6]acetone, −40 °C): *δ*=−39.3 (m, 1F, F_a_^2^J_FF_=187 Hz, bridging −OTeF_5_), −40.5 (m, 1F, F_a_, ^2^J_FF_ =185 Hz, terminal −OTeF_5_), −45.3 (m, 4F, F_b_, terminal −OTeF_5_), −45.9 (m, 4F, F_b_, bridging −OTeF_5_) ppm. ^27^Al NMR (104 MHz, SO_2_ClF, ext. [D6]acetone, −40 °C): *δ*=48.2 (br, s, FWHM=520 Hz). IR (ATR, 25 °C): $\tilde{v}$ =1452 (vw), 1220 (w), 977 (m), 868 (w), 736 (s), 709 (vs), 692 (vs), 593 (m) cm^−1^. FT‐Raman (25 °C): $\tilde{v}$ =2977 (w), 2920 (s), 1220 (m), 986 (w), 850 (w), 723 (vs), 689 (m), 675 (s), 664 (m), 649 (m), 357 (m), 330 (m), 309 (m), 275 (m), 229 (w), 215 (w), 134 (s) cm^−1^.

**Improved synthesis of [Al(OTeF_5_)_3_]_2_**: An excess of HOTeF_5_ (1234 mg, 5.10 mmol) was condensed onto a frozen solution of AlMe_3_ (105 mg, 1.46 mmol) in n‐pentane at −196 °C. The mixture was warmed to −30 °C and vigorously stirred until the gas evolution ceased. A colorless solid precipitated. Afterwards, the solution was further heated to room temperature and stirred for an additional 20 minutes, which gave rise to a continued formation of gas. Subsequently, all volatiles were pumped off leading to the isolation of a colorless powder (1063 mg, 98 %). IR (ATR, 25 °C): $\tilde{v}$ =1009 (m), 976 (m), 860 (vw), 746 (m), 704 (vs), 670 (s), 656 (s), 571 (w) cm^−1^. FT‐Raman (25 °C): $\tilde{v}$ =1023 (vw), 731 (m), 706 (s), 688 (vs), 664 (s), 613 (w), 379 (w), 327 (m), 311 (m), 136 (m) cm^−1^.

**[Al(OTeF_5_)_3_(MeCN)_3_]**: AlEt_3_ (148 mg, 1.3 mmol) was dissolved in 10 mL of MeCN. Three equivalents HOTeF_5_ (944 mg, 3.9 mmol) were condensed onto the frozen solution at −196 °C. Warming up the mixture to room temperature under constant stirring led to a gas evolution. After 30 minutes, all volatiles were removed under reduced pressure until a colorless powder was obtained (1.12 g, 99 %). ^1^H NMR (400 MHz, CD_3_CN, 22 °C): *δ*=2.0 (s, MeCN) ppm. ^19^F NMR (377 MHz, CD_3_CN, 22 °C): *δ* =−31.7 (m, 1F, F_a_, ^2^ *J*_FF_ =185 Hz, [Al(OTeF_5_)_4_(MeCN)_2_]^−^, 20 %), −32.7 (m, 1F, F_a_, ^2^ *J*_FF_ =185 Hz, [Al(OTeF_5_)_2_(MeCN)_4_]^+^, 10 %), −33.1 (m, 1F, F_a_, ^2^J_FF_ =185 Hz, [Al(OTeF_5_)_3_(MeCN)_3_]), 100 %), −45.5 (m, 4F, F_b_, [Al(OTeF_5_)_4_(MeCN)_2_]^−^), −45.75 (m, 4F, F_b_, [Al(OTeF_5_)_2_(MeCN)_4_]^+^), −45.8 (m, 4F, F_b_, [Al(OTeF_5_)_3_(MeCN)_3_]) ppm. ^27^Al NMR (104 MHz, CD_3_CN, 22 °C): *δ* =−11.7 (br, [Al(OTeF_5_)_4_(MeCN)_2_]^−^), −16.4 (br, [Al(OTeF_5_)_3_(MeCN)_3_]), −21.6 (br, [Al(OTeF_5_)_2_(MeCN)_4_]^+^) ppm. IR (ATR, 25 °C): $\tilde{v}$ =3019 (vw), 2950 (vw), 2339 (w), 2312 (w), 1418 (vw), 1371 (vw), 1035 (vw), 950 (m), 904 (m), 678 (vs), 626 (w), 553 (w), 467 (s), 448 (s) cm^−1^. FT‐Raman (25 °C): $\tilde{v}$ =3019 (vw), 2951 (vs), 2342 (s), 2314 (m), 1422 (vw), 1374 (w), 962 (w), 684 (s), 628 (m), 433 (m), 340 (w), 299 (w) cm^−1^.

**[Al(OTeF_5_)_3_(PhCN)_3_]**: AlEt_3_ (200 mg, 1.75 mmol) was dissolved in 10 mL of PhCN. Three equivalents HOTeF_5_ (1270 mg, 5.3 mmol) were condensed onto the frozen solution at −196 °C. Warming up the mixture to room temperature under constant stirring led to a gas evolution. The obtained colorless solution was concentrated under reduced pressure until a colorless powder was obtained (1.78 g, 95 %). In PhCN: ^1^H NMR (400 MHz, PhCN, ext. [D6]acetone, 22 °C): *δ*=7.38 (m, PhCN) ppm. ^19^F NMR (377 MHz, PhCN, ext. [D6]acetone, 22 °C): *δ*=−29.4 (m, 1F, F_a_, ^2^ *J*_FF_ =188 Hz, [Al(OTeF_5_)_3_(PhCN)_3_], 50 %), −30.2 (m, 1F, F_a_, ^2^ *J*_FF_ =188 Hz, [Al(OTeF_5_)_4_(PhCN)_2_]^−^, 100 %), −30.6 (m, 1F, F_a_, ^2^ *J*_FF_ =188 Hz, [Al(OTeF_5_)_2_(PhCN)_4_]^+^), 50 %), −43.4 (m, 4F, F_b_, [Al(OTeF_5_)_3_(PhCN)_3_]), −43.5 (m, 4F, F_b_, [Al(OTeF_5_)_4_(PhCN)_2_]^−^), −43.6 (m, 4F, F_b_, [Al(OTeF_5_)_2_(PhCN)_4_]^+^) ppm. ^27^Al NMR (104 MHz, PhCN, ext. [D6]acetone, 22 °C): *δ* =−13.0 (br, FWHM=1720 Hz) ppm. In CD_2_Cl_2_: ^1^H NMR (400 MHz, CD_2_Cl_2_, 22 °C): *δ*=7.69 (br, m, PhCN) ppm. ^19^F NMR (377 MHz, CD_2_Cl_2_, 22 °C): *δ* =−33.6 (m, 1F, F_a_, ^2^ *J*_FF_ =189 Hz, [Al(OTeF_5_)_2_(PhCN)_4_]^+^, 34 %), −38.7 (m, 1F, F_a_, ^2^ *J*_FF_ =188 Hz, [Al(OTeF_5_)_4_]^−^, 68 %), −38.8 (m, 1F, F_a_, ^2^ *J*_FF_ =188 Hz, [Al(OTeF_5_)_3_(PhCN)_3_]), 100 %), −44.3 (m, 4F, F_b_, [Al(OTeF_5_)_2_(PhCN)_4_]^+^), −45.0 (m, 4F, F_b_, [Al(OTeF_5_)_4_]^−^), −46.0 (m, 4F, F_b_, [Al(OTeF_5_)_3_(PhCN)_3_]) ppm. ^27^Al NMR (104 MHz, CD_2_Cl_2_, 22 °C): *δ* =46.8 (s, [Al(OTeF_5_)_4_]^−^), −9.3 (br, s, [Al(OTeF_5_)_3_(PhCN)_3_]), −17 (br, s, [Al(OTeF_5_)_2_(PhCN)_4_]^+^) ppm. IR (ATR, 25 °C): $\tilde{v}$ =3069 (vw), 3039 (vw), 2284 (m), 1597 (w), 1489 (w), 1450 (w), 942 (m), 902 (m), 756 (m), 676 (vs), 626 (m), 560 (m), 516 (m), 455 (s) cm^−1^. FT‐Raman (25 °C): $\tilde{v}$ =3078 (m), 2293 (vs), 2231 (m), 1598 (s), 1206 (w), 1183 (w), 988 (s), 774 (vw), 685 (m), 627 (m), 603 (w), 464 (m) cm^−1^. Crystals suitable for SC‐XRD were grown by cooling a concentrated solution in PhCN slowly to −40 °C.

**[Al(OTeF_5_)_3_(SO_2_ClF)_2_]**: In a Schlenktube with a greaseless Teflon valve, an excess of SO_2_ClF (∼2 mL) was condensed onto solid [Al(OTeF_5_)_3_]_2_ (104 mg, 0.07 mmol). By subsequent warming of this mixture to −30 °C a colorless, clear solution was obtained. Removal of all volatiles at 0 °C led to the isolation of a colorless powder (124 mg, 91 %). NMR samples were prepared by directly dissolving [Al(OTeF_5_)_3_]_2_ in SO_2_ClF in a J. Young NMR tube. ^19^F NMR (377 MHz, SO_2_ClF, ext. [D6]acetone, 22 °C): *δ* =−41.5 (m, 1F, F_a_, ^2^ *J*_FF_ =187 Hz), −45.9 (m, 4F, F_b_) ppm. ^27^Al NMR (104 MHz, SO_2_ClF, ext. [D6]acetone, 22 °C): *δ* =34 (br, s, FWHM=2200 Hz) ppm. IR (ATR, 25 °C): $\tilde{v}$ =1436 (w), 1188 (w), 969 (m), 890 (w), 853 (w), 745 (m), 701 (vs), 663 (s), 572 (m), 476 (w), 447 (w) cm^−1^. FT‐Raman (25 °C): $\tilde{v}$ =1428 (vw), 1182 (vw), 703 (vs), 658 (s), 446 (s), 329 (s), 307 (s), 243 (m), 136 (m) cm^−1^. Crystals suitable for SC‐XRD were grown by cooling a concentrated solution in SO_2_ClF slowly to −80 °C.

**[Al(OTeF_5_)_3_(PhF)_2_]**: Treatment of solid [Al(OTeF_5_)_3_]_2_ (50 mg, 0.03 mmol) at −30 °C with 1 mL of PhF and subsequently stirring the mixture led to the slow dissolution of the solid material and formation of a green solution. Warming the solution to 0 °C facilitates the solution process. Evaporation of the solvents led to a greenish powder, which decomposes at room temperature to a dark, oily substance. NMR samples were prepared by directly dissolving [Al(OTeF_5_)_3_]_2_ in PhF in a J. Young NMR tube. ^1^H NMR (400 MHz, C_6_H_5_F, ext. [D6]acetone, −40 °C): *δ* =6.7 (m, 5 H, C_6_H_5_F) ppm. ^19^F NMR (377 MHz, C_6_H_5_F, ext. [D6]acetone, −40 °C): *δ* =−40.7 (m, 1F, F_a_, ^2^ *J*_FF_ =191 Hz), −46.1 (m, 4F, F_b_) ppm. ^27^Al NMR (104 MHz, C_6_H_5_F, ext. [D6]acetone, −40 °C): *δ* =46.1 (br, s) ppm. Crystals suitable for SC‐XRD were grown by cooling a concentrated solution in PhF slowly to −40 °C.

**[Al(OTeF_5_)_3_(OPEt_3_)]**: To a cooled solution of [Al(OTeF_5_)_3_]_2_ (104 mg, 0.13 mmol) in SO_2_ClF at −30 °C solid OPEt_3_ (17 mg, 0.84 mmol) was added under an argon stream. Stirring of the mixture led to a slightly yellow solution and crystals were grown by concentrating the mixture and subsequently cooling to −80 °C. Removing all volatiles under reduced pressure gave a colorless powder, which was washed with *n*‐pentane and dried again (109 mg, 89 %). ^1^H NMR (400 MHz, CD_2_Cl, 22 °C): *δ* =2.13 (m, 6H, −OPEt_3_), 1.31 (m, 9H, −OPEt_3_) ppm. ^19^F NMR (377 MHz, CD_2_Cl, 22 °C): *δ* =−38.7 (m, 1F, F_a_, ^2^ *J*_FF_=188.2 Hz), −45.3 (m, 4F, F_b_) ppm. ^27^Al NMR (104 MHz, CD_2_Cl, 22 °C): *δ* =46.3 (s, [Al(OTeF_5_)_3_(OPEt_3_)]) ppm. ^31^P{^1^H} NMR (162 MHz, CD_2_Cl, 22 °C): *δ* =83.9 (s, −OPEt_3_) ppm. IR (ATR, 25 °C): $\tilde{v}$ =2994 (vw), 2956 (vw), 2922 (vw), 2894 (vw), 1460 (w), 1409 (w), 1277 (w), 1139 (m), 1048 (w), 928 (s), 793 (m), 783 (m), 691 (vs), 651 (s), 442 (m) cm^−1^. FT‐Raman (25 °C): $\tilde{v}$ =2997 (m), 2957 (s), 2925 (s), 2895 (m), 2765 (vw), 1470 (w), 1410 (w), 1235 (vw), 1043 (w), 985 (w), 794 (vw), 696 (vs), 648 (vs), 551 (w), 433 (m), 332 (s), 302 (m), 235 (m), 140 (m) cm^−1^.

**[Al(OTeF_5_)_3_(Et_2_O)_2_]**: To a cooled solution of [Al(OTeF_5_)_3_]_2_ (120 mg, 0.08 mmol) in SO_2_ClF at −30 °C a slight excess of Et_2_O (0.02 mL, 0.20 mmol) was added under an argon stream. Stirring of the mixture led to a dark colored solution. Concentrating the mixture under reduced pressure and subsequent cooling to −80 °C yielded the formation of colorless crystals suitable for SC‐XRD measurement. Attempts to isolate the crystals by removing all volatiles led to decomposition of the sample.

**[Al(OTeF_5_)_3_(*η* ^1^‐C_7_H_8_)]**: AlEt_3_ (93 mg, 0.82 mmol) was dissolved in 2 mL of toluene, the solution was frozen at −196 °C and HOTeF_5_ (593 mg, 2.45 mmol) was condensed on top. Warming the mixture to −30 °C under stirring gave a yellow/orange colored, biphasic solution. After stirring for 10 minutes the mixture was cooled to −50 °C and a slight excess of HSiEt_3_ (0.15 mL, 1.00 mmol) was added via a syringe. A gas evolution and the decolorization of the solution was observed. Warming the solution above −40 °C led to decomposition. ^1^H NMR (400 MHz, C_7_H_8_, ext. [D6]acetone, −60 °C): *δ* =0.63 (t, 9H, ^2^ *J*_HH_=8 Hz, −C*H_3_*, Et_3_SiOTeF_5_), 0.28 (quart, 6H, ‐CH_2_‐, Et_3_SiOTeF_5_) ppm. ^19^F NMR (377 MHz, C_7_H_8_, ext. [D6]acetone, −60 °C): *δ* =−37.5 (m, 1F, F_a_, ^2^ *J*_FF_ =191 Hz, Et_3_SiOTeF_5_, 33 %), −38.6 (m, 1F, F_a_, ^2^ *J*_FF_=191 Hz, [Al(OTeF_5_)_3_(tol)], 100 %), −42.9 (m, 4F, F_b_, Et_3_SiOTeF_5_), −44.1 (m, 4F, F_b_, [Al(OTeF_5_)_3_(tol)]) ppm. ^27^Al NMR (104 MHz, C_7_H_8_, ext. [D6]acetone, −60 °C): *δ* =48.4 (br, FWHM=237 Hz) ppm. Crystals suitable for SC‐XRD were grown within 2  days by cooling a concentrated solution slowly to −80 °C.

**Formation of [C(C_6_H_5_)_3_][Al(OTeF_5_)_4‐*n*_** **Cl**_***n***_ **] (*n*=0,1,2,3)**: To a cooled solution of [Al(OTeF_5_)_3_]_2_ (66 mg, 0.045 mmol) in SO_2_ClF at −30 °C a stoichiometric amount of C(C_6_H_5_)_3_Cl (24 mg, 0.09 mmol) was added under an argon stream, which resulted in the formation of a clear, bright yellow solution. The mixture was brought to room temperature and stirred for 15 minutes. Subsequently, all volatiles were removed under reduced pressure. The remaining yellow solid was washed with *n*‐pentane and dried again, resulting in a yellow powder (88.3 mg, 96 %). ^1^H NMR (400 MHz, CD_2_Cl_2_, 22 °C): *δ* =8.30 (br, 3H, *para*‐H), 7.92 (br, 6H, *meta*‐H), 7.70 (br, 6H, *ortho*‐H) ppm. ^19^F NMR (377 MHz, CD_2_Cl_2_, 22 °C): *δ*=−36.8 (m, 1F, F_a_, ^2^ *J*_FF_=189 Hz, [AlCl_2_(OTeF_5_)_2_]^−^, 11 %), −37.7 (m, 1F, F_a_, ^2^ *J*_FF_=190 Hz, [AlCl(OTeF_5_)_3_]^−^, 84 %), −38.0 (m, 1F, F_a_, ^2^J_FF_=189 Hz, [AlCl_3_(OTeF_5_)]^−^, 1 %), −38.6 (m, 1F, F_a_, ^2^ *J*_FF_=191 Hz, [Al(OTeF_5_)_4_]^−^, 100 %), −44.7 (m, 4F, F_b_, [AlCl_2_(OTeF_5_)_2_]^−^), −45.4 (m, 4F, F_b_, [AlCl(OTeF_5_)_3_]^−^), −45.6 (m, 4F, F_b_, [AlCl_3_(OTeF_5_)]^−^), −45.9 (m, 4F, F_b_, [Al(OTeF_5_)_4_]^−^) ppm. ^27^Al NMR (104 MHz, CD_2_Cl_2_, 22 °C): *δ* =92.8 (s, [Al(OTeF_5_)Cl_3_]^−^), 79.5 (s, [Al(OTeF_5_)_2_Cl_2_]^−^; d, [Al(OTeF_5_)(O^125^TeF_5_)Cl_2_]^−^, ^2^ *J*(^27^Al,^125^Te)=20 Hz), 64.1 (s, [Al(OTeF_5_)_3_Cl]^−^; d, [Al(OTeF_5_)_2_(O^125^TeF_5_)Cl]^−^, ^2^ *J*(^27^Al,^125^Te)=43 Hz), 47.0 (s, [Al(OTeF_5_)_4_]^−^; d, [Al(OTeF_5_)_3_(O^125^TeF_5_)]^−^, ^2^ *J*(^27^Al,^125^Te)=72 Hz) ppm. IR (ATR, 25 °C): $\tilde{v}$ =1621 (vw), 1579 (s), 1483 (m), 1450 (m), 1355 (s), 1295 (m), 1187 (m), 1171 (w), 995 (m), 980 (m), 926 (s), 843 (w), 809 (w), 770 (w), 686 (vs), 622 (s), 607 (s), 536 (s), 467 (m), 425 (w), 403 (m) cm^−1^. FT‐Raman (25 °C): $\tilde{v}$ =3071 (w), 1597 (s), 1583 (vs), 1485 (m), 1357 (s), 1298 (w), 1186 (m), 1174 (w), 1027 (w), 998 (m), 916 (w), 697 (w), 623 (w), 468 (w), 405 (m), 287 (s), 143 (m) cm^−1^.

**Formation of [PPh_4_][Al(OTeF_5_)_4‐*n*_** **Cl**_***n***_ **] (*n*=0,1,2,3)**: In a J. Young NMR tube, a cooled solution of [Al(OTeF_5_)_3_]_2_ (100 mg, 0.07 mmol, 0.5 equiv.) in PhF at 0 °C was treated with a stoichiometric amount of [PPh_4_]Cl (50 mg, 0.14 mmol, 1 equiv.). A colorless, clear solution formed, which was analyzed by NMR spectroscopy. Signals of the cation in the ^1^H NMR spectrum are partly overlaid by solvent signals. ^1^H NMR (400 MHz, C_6_H_5_F, ext. [D6]acetone, 22 °C): *δ* =7.42 (m, 4H, *para*‐H, [PPh_4_]^+^) 7.26 (m, 8H, *meta*‐H, [PPh_4_]^+^), 7.1–6.7 (PhF) ppm. ^19^F NMR (377 MHz, C_6_H_5_F, ext. [D6]acetone, 22 °C): *δ* =^−^36.1 (m, 1F, F_a_, ^2^ *J*_FF_=189 Hz, [AlCl_2_(OTeF_5_)_2_]^−^, 5 %), −37.2 (m, 1F, F_a_, ^2^ *J*_FF_=190 Hz, [AlCl(OTeF_5_)_3_]^−^, 44 %), −38.2 (m, 1F, F_a_, ^2^J_FF_=191 Hz, [Al(OTeF_5_)_4_]^−^, 100 %), −44.0 (m, 4F, F_b_, [AlCl_2_(OTeF_5_)_2_]^−^), −44.7 (m, 4F, F_b_, [AlCl(OTeF_5_)_3_]^−^), −45.3 (m, 4F, F_b_, [Al(OTeF_5_)_4_]^−^) ppm. ^27^Al NMR (104 MHz, C_6_H_5_F, ext. [D6]acetone, 22 °C): *δ* =80.5 (s, [Al(OTeF_5_)_2_Cl_2_]^−^), 65.1 (s, [Al(OTeF_5_)_3_Cl]^−^), 47.6 (s, [Al(OTeF_5_)_4_]^−^) ppm. ^31^P NMR (162 MHz, C_6_H_5_F, ext. [D6]acetone, 22 °C): *δ* =23.0 (m, [PPh_4_]^+^) ppm.

**[NEt_4_][OTeF_5_]**: [NEt_4_]Cl (445 mg, 2.69 mmol, 1 equiv.) was dissolved in *o*‐DFB (20 mL). The solution was cooled with liquid nitrogen and degassed. HOTeF_5_ (659 mg, 2.75 mmol, 1 equiv.) was condensed onto the solution. A bubbler was connected to the flask and the reaction mixture was stirred at room temperature until gas formation was no longer observed. After removal of the solvent in vacuo a yellow solid was obtained (952 mg, 96 % yield). ^1^H NMR (401 MHz, *o*‐DFB, ext. [D6]acetone, 22 °C): *δ*=3.21 (quart, 8H, ^3^ *J*_HH_=7.3 Hz, [N(C*H_2_* CH_3_)_4_]^+^), 1.32 (tt, 12H, ^3^ *J*_HH_=7.3 Hz, *J*_HN_=1.9 Hz, [N(CH_2_C*H_3_*)_4_]^+^) ppm. ^19^F NMR (377 MHz, *o*‐DFB, ext. [D6]acetone, 22 °C): *δ* =−28.96 (m, 1F_a_), −42.97 (m, 4F_b_) ppm. IR (ATR, 25 °C): $\tilde{v}$ =3000 (w), 1478 (m), 1458 (m), 1397 (m), 1183 (m), 1031 (w), 1002 (m), 864 (m), 762 (m), 674 (s), 632 (vs), 577 (s), 465z (w) cm^−1^. This compound was already reported in literature.^[46]^

**Formation of [NEt_4_]_2_[Al(OTeF_5_)_5_]**: A cooled solution of [Al(OTeF_5_)_3_]_2_ (100 mg, 0.07 mmol, 0.5 equiv.) in SO_2_ClF at 0 °C was treated with two equivalents of [NEt_4_][OTeF_5_] (97 mg, 0.26 mmol, 2 equiv.). The mixture was stirred for 12 h which resulted in a colorless suspension formed. All volatiles were removed under reduced pressure, which gave a colorless powder (180 mg, 94 %). For crystallization, a solution of [NEt_4_][Al(OTeF_5_)_4_] in *o*‐DFB was treated with equimolar amounts of [NEt_4_][OTeF_5_] and then slowly cooled down to −40 °C which resulted in the growth of colorless crystals suitable for SC‐XRD. ^1^H NMR (400 MHz, C_6_H_4_F_2_, ext. [D6]acetone, 22 °C): *δ*=3.02 (quart., 8H, ^3^ *J*_HH_=7.3 Hz, [N(C*H_2_* CH_3_)_4_]^+^), 1.14 (m, 12H, [N(CH_2_C*H_3_*)_4_]^+^) ppm. ^19^F NMR (377 MHz CD_2_Cl_2_, 22 °C): *δ* =−45.4 (br, m, 4F, F_b_, FWHM=340 Hz), ppm. ^27^Al NMR (104 MHz, C_6_H_4_F_2_, ext. [D6]acetone, 22 °C): *δ* =48.7 (br, FWHM=550 Hz) ppm. IR (ATR, 25 °C): $\tilde{v}$ =3003 (w), 1486 (m), 1457 (w), 1443 (w), 1396 (m), 1368 (w), 1305 (w), 1238 (w), 1173 (m), 1068 (w), 1052 (w), 999 (m), 898 (s), 783 (m), 678 (vs), 620 (m), 535 (m), 511 (m), 486 (m) cm^−1^.

**Reaction of [Al(OTeF_5_)_3_(SO_2_ClF)_2_] with [PPh_4_][SbF_6_]**: In a J. Young NMR tube, a cooled solution of [Al(OTeF_5_)_3_]_2_ (40 mg, 0.025 mmol, 0.5 equiv.) in SO_2_ClF at −30 °C was treated with a stoichiometric amount of [PPh_4_][SbF_6_] (30 mg, 0.05 mmol, 1 equiv.). The mixture was brought to room temperature and a yellowish solution together with a brown precipitate formed. The sample was analyzed by NMR spectroscopy. ^1^H MR (400 MHz, SO_2_ClF, ext. [D6]acetone, 22 °C): *δ*=8.34 (m, 4H, *para*‐H, [PPh_4_]^+^), 8.18 (m, 8H, *meta*‐H, [PPh_4_]^+^), 8.07 (m, 8H, *ortho*‐H, [PPh_4_]^+^), 6.87 (HOTeF_5_) ppm. ^19^F NMR (377 MHz, SO_2_ClF, ext. [D6]acetone, 22 °C): *δ*=−39.5 (m, 1F, F_a_, ^2^J_FF_=191 Hz, [Al(OTeF_5_)_4_]^−^, 100 %), −44.0 (m, 1F, F_a_, ^2^ *J*_FF_=191 Hz, HOTeF_5_, 10 %), −46.4 (m, 4F, F_b_, [Al(OTeF_5_)_4_]^−^), −47.8 (m, 4F, F_b_, HOTeF_5_) ppm. ^27^Al NMR (104 MHz, SO_2_ClF, ext. [D6]acetone, 22 °C): *δ* =47.4 (s, [Al(OTeF_5_)_4_]^−^) ppm. ^31^P NMR (162 MHz, SO_2_ClF ext. [D6]acetone, 22 °C): *δ* =23.9 (m, [PPh_4_]^+^) ppm.

**Reaction of [Al(OTeF_5_)_3_(SO_2_ClF)_2_] with Me_3_SiCl**: In a J. Young NMR tube, a cooled solution of [Al(OTeF_5_)_3_]_2_ (63 mg, 0.04 mmol) in SO_2_ClF at −30 °C was treated with a slight excess of trimethylsilyl chloride (13 mg, 0.12 mmol) by condensing it onto the solution. Shaking the mixture resulted in a clear, slightly yellow solution. The mixture was brought to room temperature and analyzed by NMR spectroscopy. ^1^H NMR (400 MHz, SO_2_ClF, ext. [D6]acetone, 22 °C): *δ* =0.88 (s, 9H, −C*H_3_*, Me_3_SiCl), 0.86 (m, 9H, −C*H_3_*, Me_3_SiOTeF_5_) ^19^F NMR (377 MHz, SO_2_ClF, ext. [D6]acetone, 22 °C): *δ* =−40.5 (m, 1F, F_a_, ^2^ *J*_FF_=185 Hz, Me_3_SiOTeF_5_), −44.3 (m, 4F, F_b,_ Me_3_SiOTeF_5_) ppm. ^29^Si NMR (80  MHz, SO_2_ClF, ext. [D6]acetone, 22 °C): *δ* =39.1 (Me_3_SiOTeF_5_), 31.0 (s, Me_3_SiCl) ppm. ^27^Al NMR (104 MHz, SO_2_ClF, ext. [D6]acetone, 22 °C): *δ* =91.7 (br, s, AlCl_3_, FWHM=910 Hz) ppm.

**Reaction of [Al(OTeF_5_)_3_(SO_2_ClF)_2_] with Me_3_SiF**: In a J. Young NMR tube, a cooled solution of [Al(OTeF_5_)_3_]_2_ (50 mg, 0.03 mmol) in SO_2_ClF at −30 °C was treated with an excess of trimethylsilyl fluoride (11 mg, 0.12 mmol) by condensing it onto the solution. Shaking the mixture resulted in a clear colorless solution. After the mixture was brought to room temperature a colorless precipitate formed. The solution was analyzed by NMR spectroscopy. ^1^H NMR (400 MHz, SO_2_ClF, ext. [D6]acetone, 22 °C): *δ*=0.88 (m, 9H, −C*H_3_*, Me_3_SiOTeF_5_), 0.71 (br, m, 9H, −C*H_3_*, Me_3_SiF) ppm. ^19^F NMR (377 MHz, SO_2_ClF, ext. [D6]acetone, 22 °C): *δ* =−40.5 (m, 1F, F_a_, ^2^ *J*_FF_=185 Hz, Me_3_SiOTeF_5_), −44.3 (m, 4F, F_b,_ Me_3_SiOTeF_5_), −158.0 (br, m, 1F, Me_3_SiF) ppm. ^29^Si NMR (80 MHz, SO_2_ClF, ext. [D6]acetone, 22 °C): *δ*=39.4 (m, Me_3_SiOTeF_5_), 32.6 (br, Me_3_SiF) ppm.

## Conflict of interest

The authors declare no conflict of interest.

1

## Supporting information

As a service to our authors and readers, this journal provides supporting information supplied by the authors. Such materials are peer reviewed and may be re‐organized for online delivery, but are not copy‐edited or typeset. Technical support issues arising from supporting information (other than missing files) should be addressed to the authors.

Supporting Information

## Data Availability

The data that support the findings of this study are available from the corresponding author upon reasonable request.

## References

[1] G. A. Olah , G. K. Surya Prakash , R. Molnr , J. Sommer , Superacid Chemistry, John Wiley & Sons, Inc, Hoboken, NJ, USA, 2009.

[2] H. Böhrer , N. Trapp , D. Himmel , M. Schleep , I. Krossing , Dalton Trans. 2015, 44, 7489. 25803574 10.1039/c4dt02822h

[3] H. D. B. Jenkins , H. K. Roobottom , J. Passmore , Inorg. Chem. 2003, 42, 2886. 12716180 10.1021/ic0206544

[4] K. O. Christe , D. A. Dixon , D. McLemore , W. W. Wilson , J. A. Sheehy , J. A. Boatz , J. Fluorine Chem. 2000, 101, 151.

[5] L. O. Müller , D. Himmel , J. Stauffer , G. Steinfeld , J. Slattery , G. Santiso-Quiñones , V. Brecht , I. Krossing , Angew. Chem. Int. Ed. Engl. 2008, 47, 7659; 18767085 10.1002/anie.200800783

[6] L. A. Körte , J. Schwabedissen , M. Soffner , S. Blomeyer , C. G. Reuter , Y. V. Vishnevskiy , B. Neumann , H.-G. Stammler , N. W. Mitzel , Angew. Chem. Int. Ed. Engl. 2017, 56, 8578; 28524451 10.1002/anie.201704097

[7a] J. Chen , E. Y.-X. Chen , Dalton Trans. 2016, 45, 6105; 26567780 10.1039/c5dt03895b

[7b] T. Belgardt , J. Storre , H. W. Roesky , M. Noltemeyer , H.-G. Schmidt , Inorg. Chem. 1995, 34, 3821.

[8] D. M. C. Ould , J. L. Carden , R. Page , R. L. Melen , Inorg. Chem. 2020, 59, 14891. 32869993 10.1021/acs.inorgchem.0c01076 PMC7581293

[9] A. Kraft , N. Trapp , D. Himmel , H. Böhrer , P. Schlüter , H. Scherer , I. Krossing , Chem. Eur. J. 2012, 18, 9371. 22736574 10.1002/chem.201200448

[10] J. F. Kögel , D. A. Sorokin , A. Khvorost , M. Scott , K. Harms , D. Himmel , I. Krossing , J. Sundermeyer , Chem. Sci. 2018, 9, 245. 29629094 10.1039/c7sc03988c PMC5869307

[11] I. M. Riddlestone , S. Keller , F. Kirschenmann , M. Schorpp , I. Krossing , Eur. J. Inorg. Chem. 2019, 2019, 59.

[12] J. F. Kögel , A. Y. Timoshkin , A. Schröder , E. Lork , J. Beckmann , Chem. Sci. 2018, 9, 8178. 30568768 10.1039/c8sc02981d PMC6256356

[13] L. Greb , Chem. Eur. J. 2018, 24, 17881. 29943864 10.1002/chem.201802698

[14] A. Wiesner , T. W. Gries , S. Steinhauer , H. Beckers , S. Riedel , Angew. Chem. Int. Ed. Engl. 2017, 56, 8263; 28558157 10.1002/anie.201702807

[15] A. Martens , O. Petersen , H. Scherer , I. Riddlestone , I. Krossing , Organometallics 2018, 37, 706.

[16] R. G. Vranka , E. L. Amma , J. Am. Chem. Soc. 1967, 89, 3121.

[17] A. Wiesner , L. Fischer , S. Steinhauer , H. Beckers , S. Riedel , Chem. Eur. J. 2019, 25, 10441. 31090983 10.1002/chem.201901651

[18] J. Mason , Multinuclear NMR, Springer US, Boston, MA, 1987.

[19a] K. Knabel , H. Nöth , Z. Naturforsch. B 2005, 60, 1027;

[19b] N. C. Means , C. M. Means , S. G. Bott , J. L. Atwood , Inorg. Chem. 1987, 26, 1466.

[20] K. F. Hoffmann , A. Wiesner , N. Subat , S. Steinhauer , S. Riedel , Z. Anorg. Allg. Chem. 2018, 644, 1344.

[21] F. O′Donnell , D. Turnbull , S. D. Wetmore , M. Gerken , Chem. Eur. J. 2021, 27, 16334. 34559930 10.1002/chem.202103221

[22] G. Portalone , G. Schultz , A. Domenicano , I. Hargittai , J. Mol. Struct. 1984, 118, 53.

[23a] T. Birchall , R. J. Gillespie , Spectrochim. Acta 1966, 22, 681;

[23b] R. J. Gillespie , E. A. Robinson , Spectrochim. Acta 1962, 18, 1473.

[24a] P. Ulferts , K. Seppelt , Z. Anorg. Allg. Chem. 2004, 630, 1589;

[24b] H. P. A. Mercier , M. D. Moran , J. C. P. Sanders , G. J. Schrobilgen , R. J. Suontamo , Inorg. Chem. 2005, 44, 49. 15627360 10.1021/ic0400890

[25] G. S. Hair , A. H. Cowley , R. A. Jones , B. G. McBurnett , A. Voigt , J. Am. Chem. Soc. 1999, 121, 4922.

[26] A. Wiesner , S. Steinhauer , H. Beckers , C. Müller , S. Riedel , Chem. Sci. 2018, 9, 7169. 30288235 10.1039/c8sc03023e PMC6151472

[27] J. B. Lambert , S. Zhang , C. L. Stern , J. C. Huffman , Science 1993, 260, 1917. 17836721 10.1126/science.260.5116.1917

[28] P. Erdmann , J. Leitner , J. Schwarz , L. Greb , ChemPhysChem 2020, 21, 987. 32212357 10.1002/cphc.202000244 PMC7317340

[29a] V. Gutmann , Electrochim. Acta 1976, 21, 661;

[29b] Y. Marcus , J. Solution Chem. 1984, 13, 599.

[30a] U. Mayer , V. Gutmann , W. Gerger , Monatsh. Chem. 1975, 106, 1235;

[30b] M. A. Beckett , G. C. Strickland , J. R. Holland , K. Sukumar Varma , Polymer 1996, 37, 4629.

[31] S. Mummadi , D. Kenefake , R. Diaz , D. K. Unruh , C. Krempner , Inorg. Chem. 2017, 56, 10748. 28820241 10.1021/acs.inorgchem.7b01719

[32] P. Erdmann , L. Greb , Angew. Chem. Int. Ed. Engl. 2022, 61, e202114550; 34757692 10.1002/anie.202114550 PMC9299668

[33a] M. Rohde , L. O. Müller , D. Himmel , H. Scherer , I. Krossing , Chem. Eur. J. 2014, 20, 1218; 24435914 10.1002/chem.201303671

[33b] A. Martens , P. Weis , M. C. Krummer , M. Kreuzer , A. Meierhöfer , S. C. Meier , J. Bohnenberger , H. Scherer , I. Riddlestone , I. Krossing , Chem. Sci. 2018, 9, 7058. 30310626 10.1039/c8sc02591f PMC6137444

[34] M. A. Ellwanger , C. von Randow , S. Steinhauer , Y. Zhou , A. Wiesner , H. Beckers , T. Braun , S. Riedel , Chem. Commun. 2018, 54, 9301. 10.1039/c8cc05233f 30070660

[35] K. F. Hoffmann , D. Battke , P. Golz , S. M. Rupf , M. Malischewski , S. Hasenstab-Riedel , Angew. Chem. Int. Ed. Engl. 2022, e202203777; 35416383 10.1002/anie.202203777 PMC9401592

[36] K. Seppelt , D. Nothe , Inorg. Chem. 1973, 12, 2727.

[37] R. K. Harris , E. D. Becker , S. M. Cabral de Menezes , P. Granger , R. E. Hoffman , K. W. Zilm , Pure Appl. Chem. 2008, 80, 59.

[38] *gNMR V 5.0*, Adept Scientific, **2005**.

[39] G. M. Sheldrick , Acta Crystallogr. Sect. A 2008, 64, 112.18156677

[40] G. M. Sheldrick , Acta Crystallogr. Sect. C 2015, 71, 3. 10.1107/S2053273314026370 PMC4283466 25537383

[41] O. V. Dolomanov , L. J. Bourhis , R. J. Gildea , J. A. K. Howard , H. Puschmann , J. Appl. Crystallogr. 2009, 42, 339. 10.1107/S0021889811041161 PMC3236671 22199401

[42] TURBOMOLE GmbH, *TURBOMOLE V7.3. a development of University of Karlsruhe and Forschungszentrum Karlsruhe GmbH*, **2018**.

[43a] A. D. Becke , Phys. Rev. A 1988, 38, 3098; 10.1103/physreva.38.3098 9900728

[43b] C. Lee , W. Yang , R. G. Parr , Phys. Rev. B 1988, 37, 785; 10.1103/physrevb.37.785 9944570

[43c] S. H. Vosko , L. Wilk , M. Nusair , Can. J. Phys. 1980, 58, 1200.

[44] M. Sierka , A. Hogekamp , R. Ahlrichs , J. Chem. Phys. 2003, 118, 9136.

[45] F. Weigend , R. Ahlrichs , Phys. Chem. Chem. Phys. 2005, 7, 3297. 16240044 10.1039/b508541a

[46] K. Moock , K. Seppelt , Z. Anorg. Allg. Chem. 1988, 561, 132.

