# Supplementary material for: Insights on the Lewis Superacid Al(OTeF5)3: Solvent Adducts, Characterization and Properties
Source: Chemistry. 2022 Aug 18;28(57):e202201958. doi: 10.1002/chem.202201958 (PMC9804164; doi:10.1002/chem.202201958)
Supplement: Supplementary file 1 — Supporting Information [file CHEM-28-0-s001.pdf]

Chemistry–A European Journal

Supporting Information

Insights on the Lewis Superacid $Al(OTeF_5)_3$: Solvent Adducts, Characterization and Properties

Kurt F. Hoffmann, Anja Wiesner, Simon Steinhauer, and Sebastian Riedel*

Table of Content

NMR Spectra 2
Vibrational Spectra 5
Crystal data 6
Quantum-chemical calculations 7

NMR Spectra

NMR spectra of $[C(C_6H_5)_3][Al(OTeF_5)_{4-n}Cl_n]$ (n = 0,1,2,3)

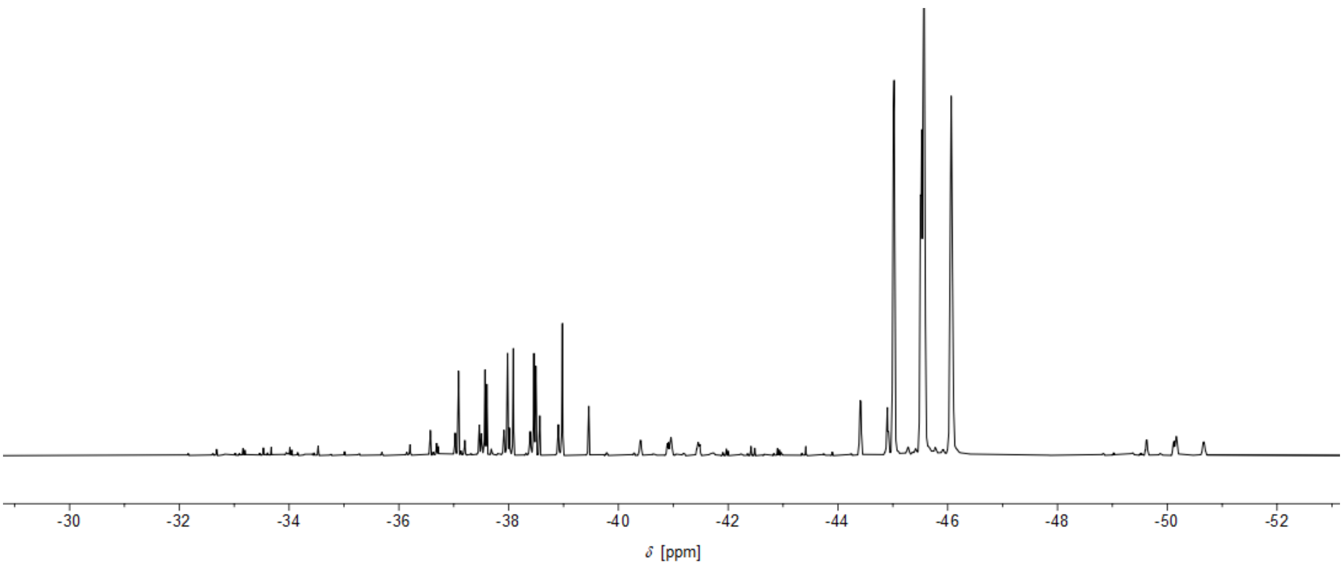

Figure S1. ^{19}F NMR spectrum (377 MHz, CD_2Cl_2, 22 °C) of $[C(C_6H_5)_3][Al(OTeF_5)_{4-n}Cl_n]$ (n = 0,1,2,3).

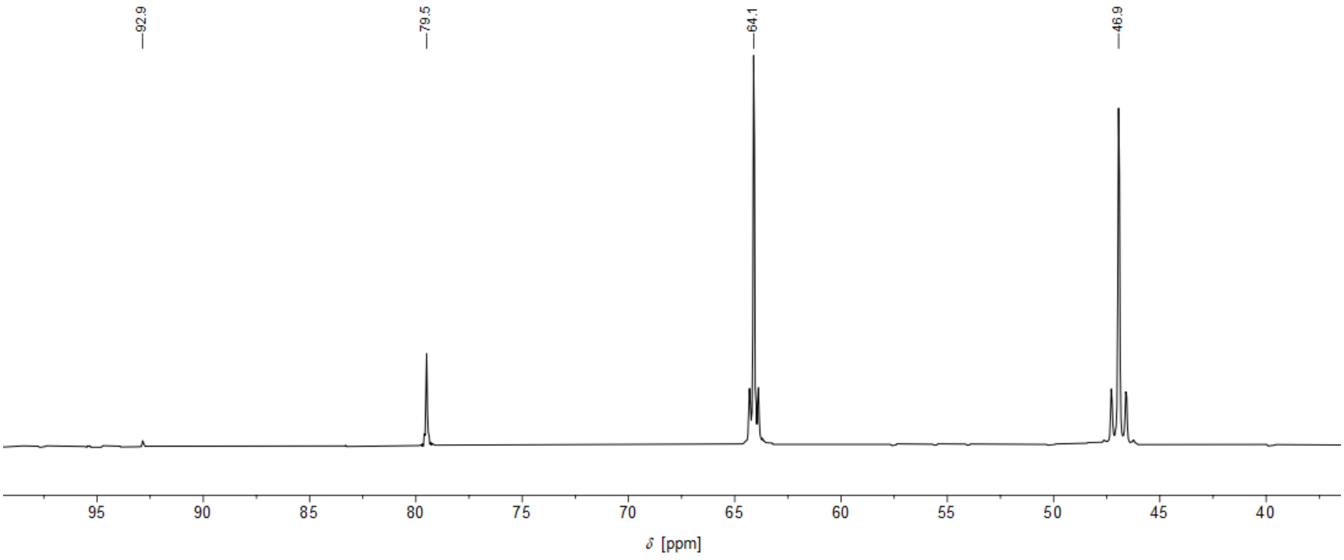

Figure S2. ^{27}Al NMR spectrum (104 MHz, CD_2Cl_2, 22 °C) of $[C(C_6H_5)_3][Al(OTeF_5)_{4-n}Cl_n]$ (n = 0,1,2,3).

NMR spectra of autoionized [Al(OTeF$_5$)$_3$(PhCN)$_3$] in CD$_2$Cl$_2$

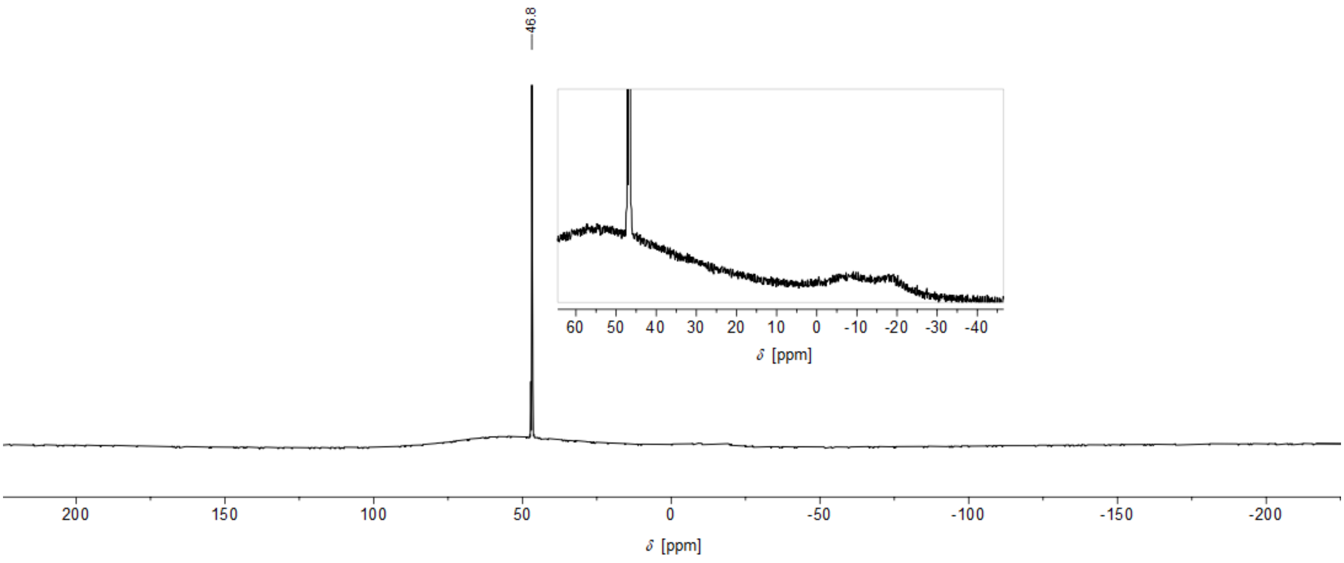

Figure S3. ^{27}Al NMR spectrum (104 MHz, CD$_2$Cl$_2$, 22 °C) of [Al(OTeF$_5$)$_3$(PhCN)$_3$] and its autoionization products..

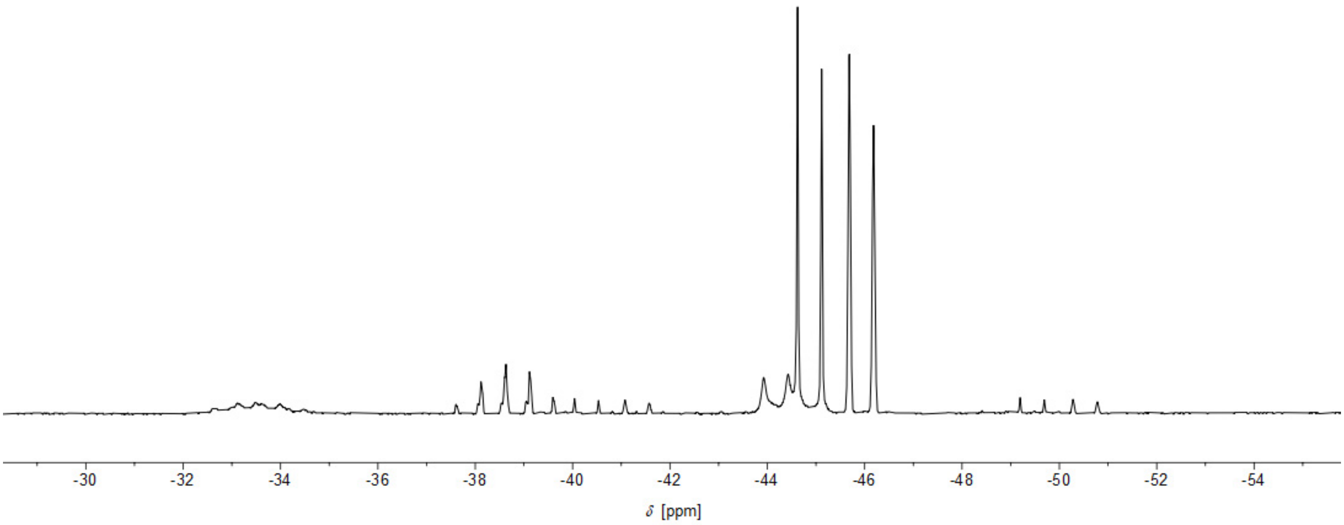

Figure S4. ^{19}F NMR spectrum (377 MHz, CD$_2$Cl$_2$, 22 °C) of [Al(OTeF$_5$)$_3$(PhCN)$_3$] and its autoionization products.

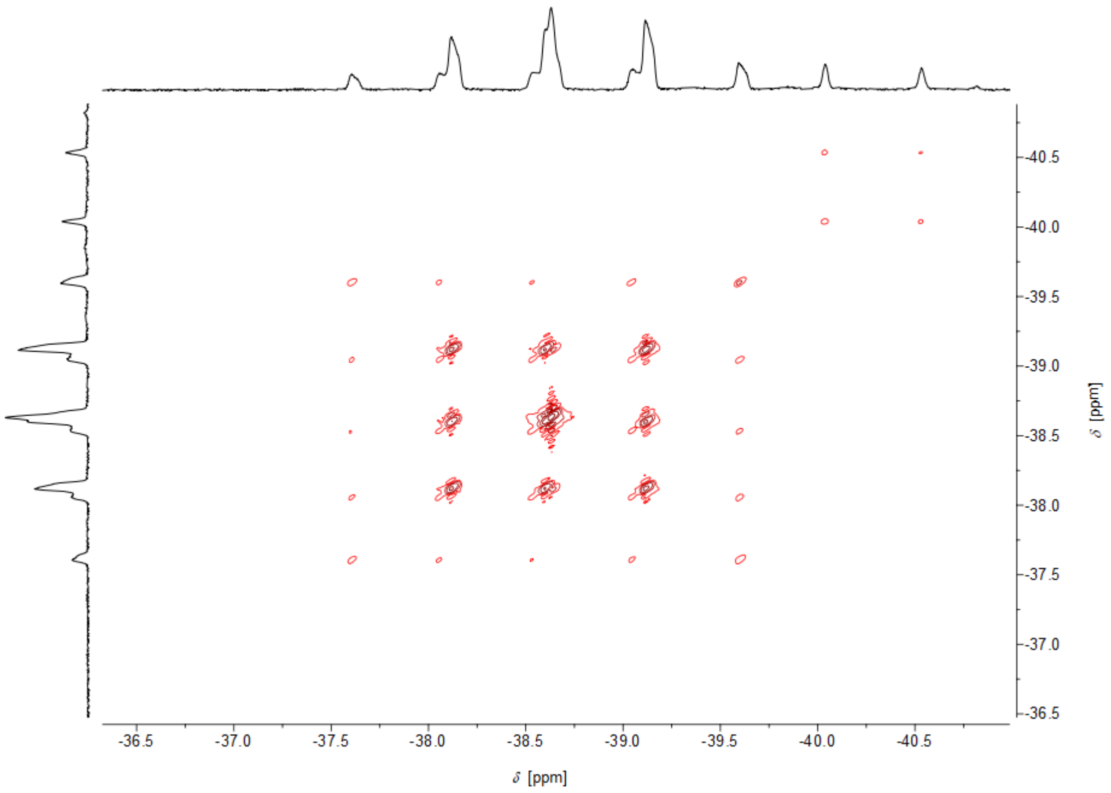

Figure S5. ^{19}F,^{19}F EXSY NMR (377 MHz, CD_2Cl_2, 22 °C, mixing time 1.0 s) of [Al(OTeF$_5$)$_3$(PhCN)$_3$].

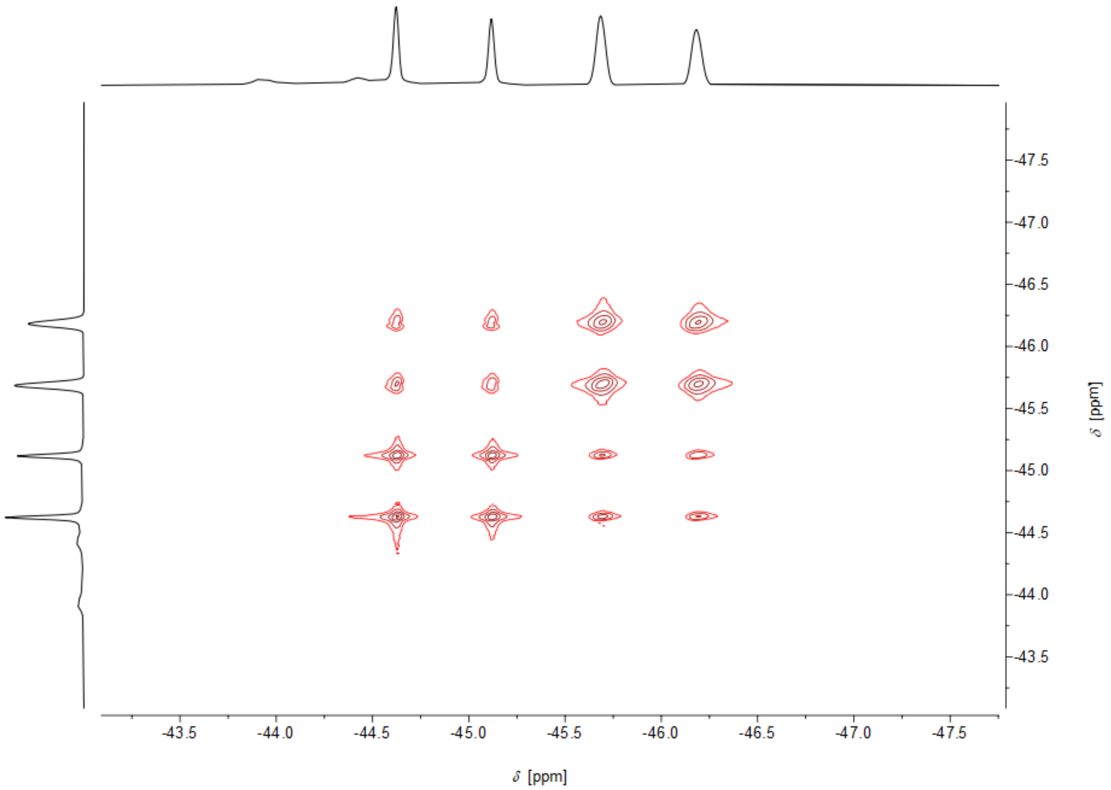

Figure S6. ^{19}F,^{19}F EXSY NMR (377 MHz, CD_2Cl_2, 22 °C, mixing time 1.0 s) of [Al(OTeF$_5$)$_3$(PhCN)$_3$].

Vibrational Spectra

Infrared spectrum of $[Al(OTeF_5)_2Me]_2$ and $[Al(OTeF_5)_3]_2$

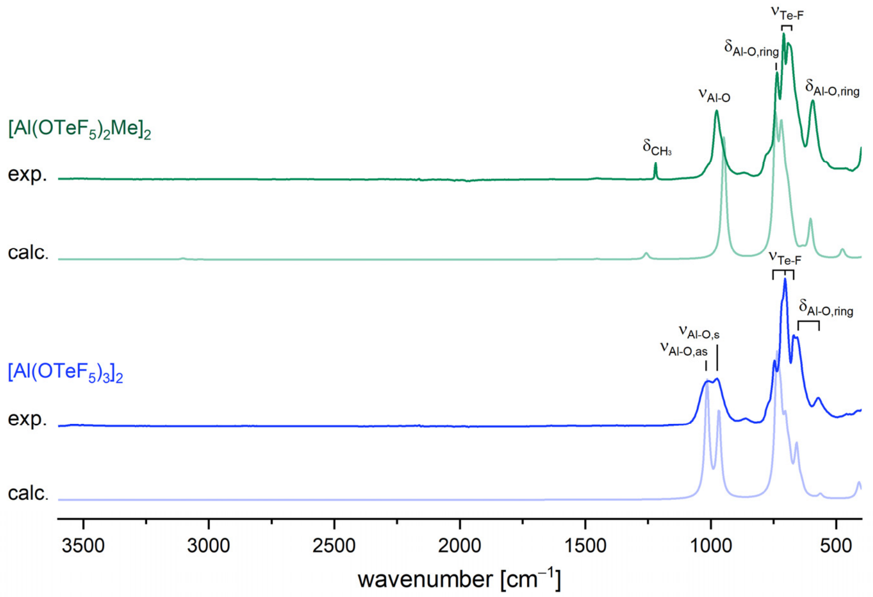

Figure S7. Experimental and calculated IR spectra of $[Al(OTeF_5)_2Me]_2$ (top, green) and $[Al(OTeF_5)_3]_2$ (bottom, blue). Calculations were performed on the B3LYP/def2-TZVPP level of theory.

Raman spectrum of $[Al(OTeF_5)_2Me]_2$ and $[Al(OTeF_5)_3]_2$

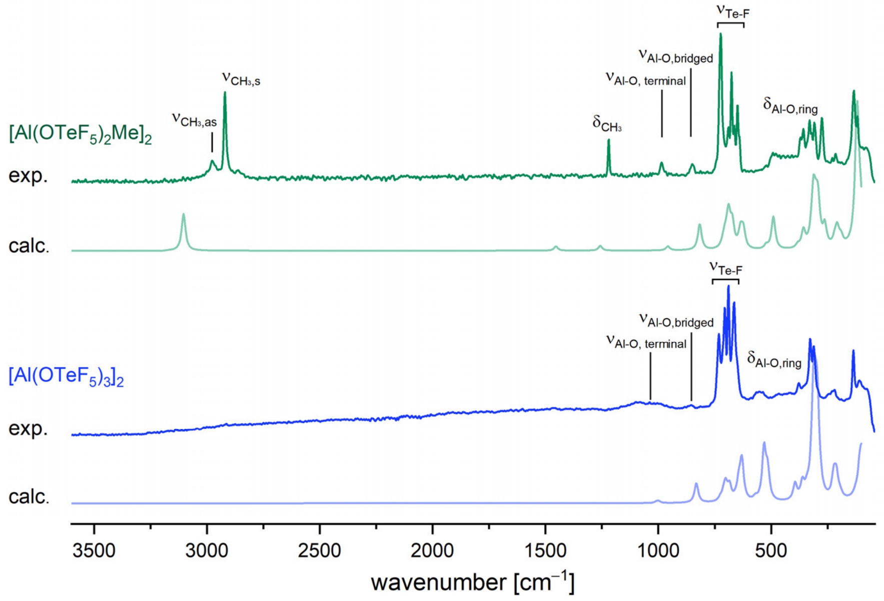

Figure S8. Experimental and calculated Raman spectra of $[Al(OTeF_5)_2Me]_2$ (top, green) and $[Al(OTeF_5)_3]_2$ (bottom, blue). Calculations were performed on B3LYP/def2-TZVPP level of theory.

Crystal data

	$[Al(OTeF_5)_2Me]_2$	$[Al(OTeF_5)_3(PhCN)_3]$	$[Al(OTeF_5)_2(bipy)_2]$ $[Al(OTeF_5)_4(bipy)]$	$[Al(OTeF_5)_3(PhF)_2]$	$[Al(OTeF_5)_3(SO_2ClF)_2]$
CCDC number	2165632	2165805	2165786	2161784	2161790
empirical formula	$C_4H_{12}Al_4F_{40}O_8Te_8$	$C_{21}H_{15}AlF_{15}N_3O_3Te_3$	$C_{30}H_{24}Al_2F_{30}N_6O_6Te_6$	$C_{12}H_{10}AlF_{17}O_3Te_3$	$AlCl_2F_{17}O_7S_2Te_3$
formula weight	2076.86	1052.14	1954.11	934.98	979.80
temperature [K]	100.0	100.0	100.0	100.0	100.0
crystal system	monoclinic	monoclinic	triclinic	monoclinic	triclinic
space group	$P2_1/n$	$P2_1/c$	$P\bar{1}$	$P2_1/n$	$P\bar{1}$
a [pm]	797.34(4)	1423.20(10)	929.15(6)	1422.04(8)	950.95(9)
b [pm]	956.42(5)	427.30(3)	1763.64(12)	867.36(5)	1034.61(9)
c [pm]	1394.58(8)	1610.83(11)	1787.83(10)	1957.19(13)	1282.11(11)
α [°]	90	90	62.239(2)	90	68.344(3)
β [°]	90.669(2)	98.909(2)	81.870(2)	103.301(2)	70.816(3)
γ [°]	90	90	89.249(2)	90	63.920(3)
volume [Å^3]	1063.42(10)	9677.8(11)	2561.8(3)	2349.3(2)	1031.89(16)
Z	1	12	2	4	2
ρ_{calcd} [g · cm^{-3}]	3.243	2.166	2.533	2.643	3.153
μ [mm^{-1}]	5.696	2.838	3.563	3.887	4.890
F(000)	928.0	5880.0	1804.0	1712.0	888.0
dimension [mm]	0.236 × 0.169 × 0.152	0.25 × 0.2 × 0.15	0.24 × 0.1 × 0.04	0.18 × 0.17 × 0.12	0.365 × 0.361 × 0.256
radiation	MoKα (λ = 0.71073)	MoKα (λ = 0.71073)	MoKα (λ = 0.71073)	MoKα (λ = 0.71073)	MoKα (λ = 0.71073)
2Θ range for data collection/°	4.258 to 59.95	4.564 to 55.664	4.436 to 56.798	5.16 to 52.822	4.546 to 55.026
index ranges	-11 ≤ h ≤ 9, -13 ≤ k ≤ 13, -19 ≤ l ≤ 19	-18 ≤ h ≤ 18, -56 ≤ k ≤ 56, -21 ≤ l ≤ 21	-12 ≤ h ≤ 12, -23 ≤ k ≤ 23, -21 ≤ l ≤ 23	-17 ≤ h ≤ 17, -10 ≤ k ≤ 10, -24 ≤ l ≤ 24	-12 ≤ h ≤ 12, -13 ≤ k ≤ 13, -16 ≤ l ≤ 16
reflections collected	24018	253100	247447	33084	12875
independent reflections	3080 [R_{int} = 0.0381, R_{sigma} = 0.0234]	22876 [R_{int} = 0.0433, R_{sigma} = 0.0200]	12835 [R_{int} = 0.0430, R_{sigma} = 0.0140]	4814 [R_{int} = 0.0528, R_{sigma} = 0.0314]	4656 [R_{int} = 0.0432, R_{sigma} = 0.0494]
data/restraints/parameters	3080/0/147	22876/0/1302	12835/282/831	4814/0/325	4656/638/496
goodness-of-fit on F^2	1.099	1.112	1.095	1.051	1.061
final *R* indexes [$I > 2\sigma(I)$]	R_1 = 0.0393, wR_2 = 0.0940	R_1 = 0.0350, wR_2 = 0.0676	R_1 = 0.0266, wR_2 = 0.0623	R_1 = 0.0242, wR_2 = 0.0510	R_1 = 0.0287, wR_2 = 0.0634
final *R* indexes [all data]	R_1 = 0.0457, wR_2 = 0.1012	R_1 = 0.0432, wR_2 = 0.0702	R_1 = 0.0312, wR_2 = 0.0652	R_1 = 0.0352, wR_2 = 0.0549	R_1 = 0.0326, wR_2 = 0.0652
largest diff. peak/hole [e Å^{-3}]	2.64/-1.83	1.41/-1.39	1.59/-2.19	0.52/-1.27	0.72/-1.44

	$[Al(OTeF_5)_3(\eta^1\text{-}C_7H_8)]$	$[Al(OTeF_5)_3(OEt_2)_2]$	$[NEt_4]_2[Al(OTeF_5)_5]$	$[Al(OTeF_5)_3(OPEt_3)]$
CCDC number	2165797	2165785	2167629	2170700
empirical formula	$C_{14}H_{16}AlF_{15}O_3Te_3$	$C_8H_{20}AlF_{15}O_5Te_3$	$C_{16}H_{40}AlF_{25}N_2O_5Te_5$	$C_6H_{15}AlF_{15}O_4PTe_3$
formula weight	927.05	891.02	1480.48	876.93
temperature [K]	100.0	100.0	100.0	150.0
crystal system	monoclinic	triclinic	monoclinic	orthorhombic
space group	$P2_1/c$	$P\bar{1}$	$P2_1/c$	*Pbca*
a [pm]	879.92(8)	948.83(5)	1863.99(18)	1590.90(7)
b [pm]	1912.07(18)	953.82(5)	1252.02(12)	1630.61(7)
c [pm]	1548.37(12)	1351.99(6)	1772.81(16)	1714.91(7)
α [°]	90	79.239(2)	90	90
β [°]	99.002(3)	72.354(2)	94.300(4)	90
γ [°]	90	87.553(2)	90	90
volume [Å^3]	2573.0(4)	1145.37(10)	4125.7(7)	4448.7(3)
Z	4	2	4	8
ρ_{calcd} [g · cm^{-3}]	2.393	2.584	2.384	2.619
μ [mm^{-1}]	3.537	3.972	3.658	4.154
F(000)	1712.0	824.0	2752.0	3216.0
dimension [mm]	0.14 × 0.13 × 0.04	0.16 × 0.094 × 0.046	0.19 × 0.14 × 0.04	0.18 × 0.15 × 0.104
radiation	MoKα (λ = 0.71073)	MoKα (λ = 0.71073)	MoKα (λ = 0.71073)	MoKα (λ = 0.71073)
2Θ range for data collection/°	4.686 to 52.126	4.348 to 61.098	4.466 to 56.74	4.294 to 52.838
index ranges	-10 ≤ h ≤ 10, -23 ≤ k ≤ 23, -19 ≤ l ≤ 18	-13 ≤ h ≤ 13, -13 ≤ k ≤ 13, -16 ≤ l ≤ 19	-24 ≤ h ≤ 24, -16 ≤ k ≤ 16, -23 ≤ l ≤ 23	-19 ≤ h ≤ 19, -20 ≤ k ≤ 20, -21 ≤ l ≤ 21
reflections collected	22774	56863	156748	70037
independent reflections	5077 [R_{int} = 0.0754, R_{sigma} = 0.0541]	7003 [R_{int} = 0.0558, R_{sigma} = 0.0311]	10271 [R_{int} = 0.0600, R_{sigma} = 0.0235]	4569 [R_{int} = 0.0559, R_{sigma} = 0.0187]
data/restraints/parameters	5077/13/271	7003/0/293	10271/991/763	4569/413/311
goodness-of-fit on F^2	1.323	1.180	1.129	1.072
final *R* indexes [$I > 2\sigma(I)$]	R_1 = 0.0678, wR_2 = 0.1188	R_1 = 0.0341, wR_2 = 0.0527	R_1 = 0.0442, wR_2 = 0.0909	R_1 = 0.0242, wR_2 = 0.0431
final *R* indexes [all data]	R_1 = 0.0825, wR_2 = 0.1223	R_1 = 0.0440, wR_2 = 0.0548	R_1 = 0.0547, wR_2 = 0.0982	R_1 = 0.0330, wR_2 = 0.0459
largest diff. peak/hole [e Å^{-3}]	1.19/-1.35	0.89/-1.60	1.37/-1.77	1.07/-1.01

Quantum-chemical calculations

$Al(OTeF_5)_3$

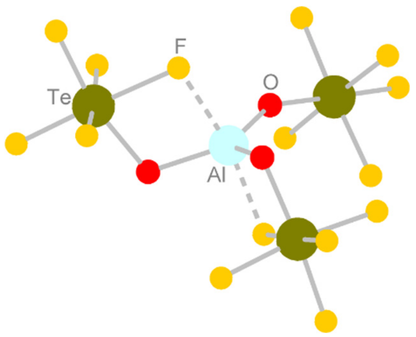

Figure S9. Representation of the B3LYP/def2-TZVPP structure of $Al(OTeF_5)_3$.

Al	-0.0154814	-0.2726284	0.2380079
O	-1.2150192	-1.6099189	0.2188792
Te	-1.858025	-1.7990648	1.9410794
F	-2.228418	-1.676358	3.7448521
F	-3.2075098	-0.5482842	1.707018
F	-0.5271402	-2.9989231	2.4273624
F	-0.5568303	-0.3105929	2.1825416
F	-3.0407199	-3.1811759	1.6577531
O	-0.5413155	1.325125	-0.0481248
Te	-0.6539851	2.8788385	-1.0391523
F	-0.7800246	4.4498125	-2.0174793
F	-2.0818445	2.2194246	-2.0275142
F	0.7737349	3.6341692	-0.1229922
F	0.5225159	2.2408741	-2.3338798
F	-1.8344846	3.6138593	0.1851539
O	1.6931933	-0.6379979	0.6599469
Te	2.5425389	-1.1453158	-0.9005797
F	2.9955182	-1.582618	-2.634162
F	3.0295077	0.5879675	-1.3401595
F	2.0716012	-2.930996	-0.7098563
F	0.6905554	-0.7726229	-1.5611246
F	4.2216328	-1.4835738	-0.2275697

$[Al(OTeF_5)_3]_2$

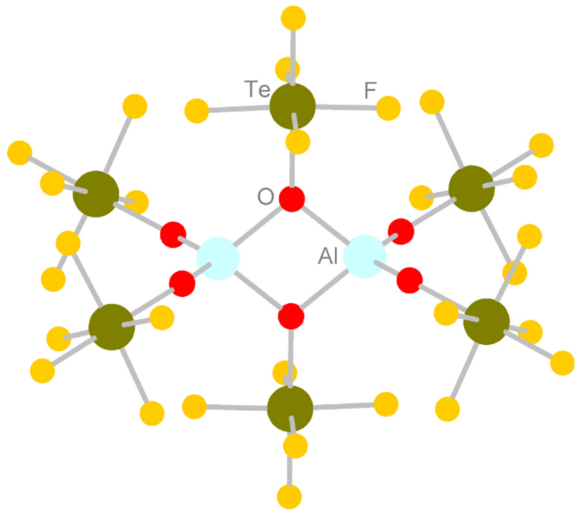

Figure S10. Representation of the B3LYP/def2-TZVPP structure of $[Al(OTeF_5)_3]_2$.

O	-1.4930305	2.1896381	-0.2767368
Te	-2.6470583	3.6140652	0.0228695
F	-3.8113447	5.0216138	0.3245977
F	-3.9988501	2.7359959	-0.8867214
F	-1.3432751	4.5522506	0.9597441
F	-2.1248447	4.4420252	-1.5503481
F	-3.2294561	2.8578109	1.6172466
O	1.47706	2.2066219	0.2369161
Te	2.6274369	3.6314827	-0.074022
F	3.7892503	5.0386468	-0.3863367
F	2.1086607	4.4664612	1.4965332
F	3.2056546	2.8673107	-1.6661743
F	1.3197935	4.5631386	-1.0116021
F	3.9833327	2.7597541	0.8358979
O	-1.4789384	-2.202007	-0.2427287
Te	-2.6340976	-3.6213529	0.0767392
F	-3.8004543	-5.0229981	0.3985197
F	-3.2089307	-2.845011	1.664112
F	-2.1183256	-4.4677639	-1.4885854
F	-1.3285911	-4.5514535	1.0188458
F	-3.9870594	-2.7508552	-0.8384706
O	1.4921224	-2.1935953	0.269627
Te	2.6436121	-3.6225345	-0.021176
F	3.8063834	-5.03311	-0.3159772
F	1.3400142	-4.5600328	-0.9584854
F	3.9937989	-2.7442902	0.8905543
F	2.1142487	-4.4446341	1.5527079
F	3.2321289	-2.8714023	-1.615457
Al	-0.0043695	1.4170581	-0.0135024
O	-0.1535843	0.0086776	1.1968077
Te	-0.3382248	0.0208286	3.116416
F	-0.5120992	0.0316488	4.9465403
F	1.4960966	0.0279101	3.3072707
F	-2.1732851	0.0141106	2.9537881
F	-0.3249469	-1.8299382	3.0611031
F	-0.3367753	1.8705495	3.0372695
Al	0.0052831	-1.4170809	0.0087401
O	0.1560708	-0.0086024	-1.2013979
Te	0.3480949	-0.0230182	-3.1202101
F	0.5300923	-0.0367044	-4.9495997
F	2.182635	-0.0131357	-2.9500178
F	-1.4852181	-0.0329699	-3.3189239
F	0.3318195	1.8276573	-3.0680115
F	0.3491701	-1.872766	-3.0383616

$[Al(OTeF_5)_3F]^-$

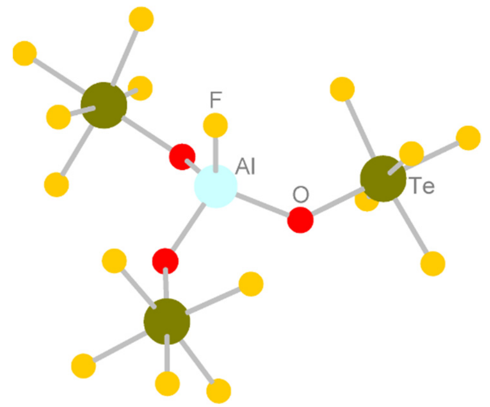

Figure S11. Representation of the B3LYP/def2-TZVPP structure of $[Al(OTeF_5)_3F]^-$.

O	-1.5463191	-0.2801372	0.5217827
Te	-2.5250278	-1.1545196	1.8577326
F	-2.7914381	-2.7104752	0.7929917
Al	0.1925518	0.0807057	0.2943045
F	2.3753416	-1.7044053	-2.6296602
Te	2.6820221	-1.9093634	-0.7611651
F	3.2215533	-2.1999151	1.0425133
F	-2.4040392	0.2908222	3.0922809
O	0.2616829	1.1881487	-1.1116047
Te	-0.3187476	2.9476271	-1.3864487
F	-1.1874354	3.0619573	0.3117506
O	0.9270971	-1.4442571	-0.2971982
F	-3.5510063	-2.0478683	3.1947791
F	-4.1702226	-0.4518669	1.2124829
F	-0.9844525	-1.9635291	2.6482528
F	-0.9019009	4.7377081	-1.6916792
F	-1.959732	2.4122816	-2.1908225
F	1.2512886	3.7254705	-0.6381403
F	0.4827542	3.0154881	-3.1098445
F	4.4599697	-2.409526	-1.2377858
F	3.3277562	-0.1133291	-0.6688633
F	2.2296214	-3.750751	-0.9126338
F	0.9286825	0.679734	1.6669752

$[Al(OTeF_5)_3(\eta^1\text{-}C_7H_8)]$

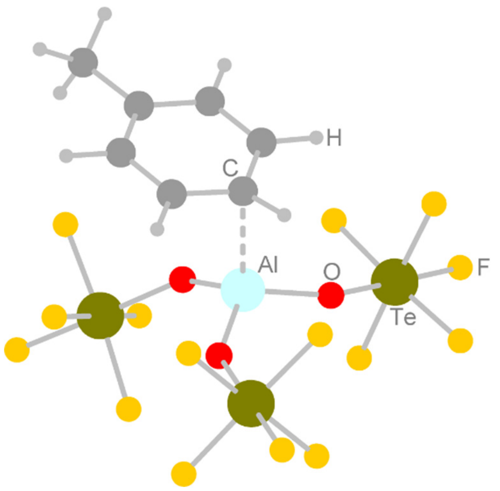

Figure S12. Representation of the B3LYP/def2-TZVPP structure of $[Al(OTeF_5)_3(\eta^1\text{-}C_7H_8)]$.

O	-1.3645142	0.0472616	0.8383873
Te	-2.0247602	-0.9220591	2.2642332
F	-0.8693236	-2.3472871	1.9657889
Al	0.0842052	0.5030815	0.0144527
F	3.2735168	-1.4518198	-0.6971698
Te	3.355718	-0.1378949	0.6109738
F	3.5812761	1.2018469	1.8908453
F	-3.2266079	0.4432291	2.6764494
O	0.1000744	0.2054927	-1.6897126
Te	-0.9183405	-0.2130364	-3.1710909
F	-2.4976445	0.3596441	-2.3556755
O	1.5215121	-0.0616924	0.8037575
F	-2.7207759	-1.8649171	3.7033038
F	-3.3136934	-1.7214252	1.1988772
F	-0.8004867	-0.1684789	3.4535181
F	-1.9271266	-0.6017863	-4.6792363
F	-1.237547	-1.9454691	-2.5830916
F	-0.6929694	1.5083491	-3.8559252
F	0.579457	-0.7800341	-4.1011201
F	5.2024022	-0.1733742	0.4403084
F	3.3354972	1.1880333	-0.713347
F	3.5018473	-1.4273841	1.9309405
C	-1.951805	3.5472675	1.5957221
C	-0.7335497	3.2601129	2.2252987
C	0.3741602	2.8735289	1.4933308
C	0.2809421	2.7344364	0.0912297
C	-0.9338018	3.0628015	-0.5485858
C	-2.0306255	3.4466468	0.201083
H	-0.6577867	3.3459344	3.3011814
H	1.3128721	2.6709089	1.9907999
H	1.1882941	2.6349594	-0.5012941
H	-1.0008055	3.0134289	-1.6268035
C	-3.1560707	3.9263926	2.4038704
H	-3.8464	4.5420932	1.8288546
H	-3.6912716	3.0230675	2.7082804
H	-2.8758123	4.4618862	3.3103096
H	-2.962405	3.6782877	-0.297653

[Al(OTeF5)3(SO2ClF)]

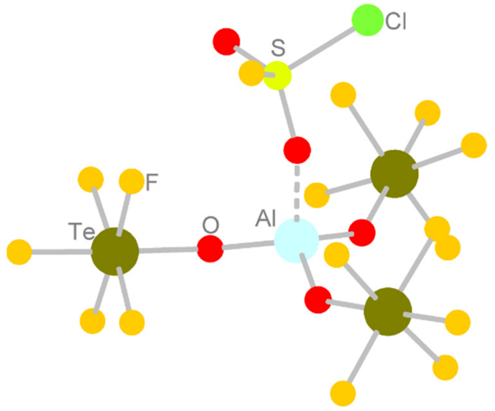

Figure S13. Representation of the B3LYP/def2-TZVPP structure of [Al(OTeF5)3(SO2ClF)].

O	-1.6592433	0.7148916	-0.6009423
Te	-3.364701	0.4311416	0.0548061
F	-3.7917254	-0.7446366	-1.3157064
Al	-0.0721226	0.0673813	-0.4207161
F	0.3395392	-3.9310304	-2.0915278
Te	1.1141499	-3.0967332	-0.634333
F	1.9519698	-2.3477315	0.8736738
F	-3.0555693	1.5639602	1.5024347
O	1.1915196	1.0607705	-1.0391452
Te	1.8705479	2.7787433	-0.958564
F	3.5627879	2.1374761	-0.5521731
O	0.0656577	-1.594959	-0.855081
F	-5.0677968	0.153948	0.7351987
F	-4.0348	1.8488294	-0.9269576
F	-2.7879589	-1.0057863	1.1098063
F	2.5684274	4.4926598	-0.8417563
F	1.4878106	2.8888833	0.876345
F	2.2857932	2.7723444	-2.7621977
F	0.2202367	3.5519195	-1.3246753
F	2.1646332	-4.6026743	-0.3734969
F	2.4720564	-2.4253162	-1.7067669
F	-0.1377897	-3.8735557	0.4995897
O	0.1651496	0.1042847	1.4736431
F	0.3061131	1.2980448	3.4921178
Cl	0.3525177	-1.3905595	3.7991572
S	0.9397938	0.1414216	2.6950853
O	2.3366892	0.2854494	2.6709802

[Al(OTeF5)3(SO2ClF)2]

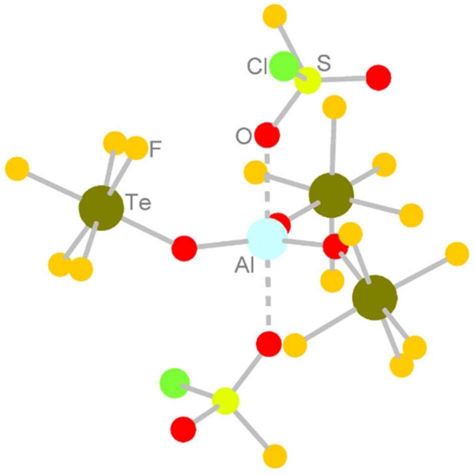

Figure S14. Representation of the B3LYP/def2-TZVPP structure of [Al(OTeF5)3(SO2ClF)2].

Te	10.0161653	6.2782047	10.9361748
Te	10.9493527	11.9846492	9.3144023
Te	8.0640841	8.3097546	5.7191609
S	6.9010151	9.5609002	10.1436922
Cl	6.0578466	11.1129064	9.2276187
Al	9.7932361	8.8235004	8.6290342
Cl	13.8320818	8.1058191	8.4863993
S	12.6225164	8.2284827	6.9077405
F	9.1625566	12.5135547	9.1991334
F	11.2861285	12.7614866	7.6655026
F	8.543996	7.0932321	11.74685
F	11.119615	6.9515188	12.2676554
F	6.919034	9.7658341	5.9196719
F	12.7567993	11.582785	9.4978082
O	6.1327563	8.3928884	9.9769105
F	11.3063798	13.6344905	10.0871165
O	10.6181329	10.3528492	8.5279562
F	6.7859619	10.0431297	11.6095795
F	11.4600028	5.3533879	10.2135727
F	9.7094279	4.8117313	12.0314282
F	8.880489	5.4909523	9.6887203
O	12.9908846	9.2970521	6.0675927
O	8.3148047	9.5908647	9.8775039
F	9.1748303	6.8556662	5.3765248
F	12.9848867	6.8938579	6.2097644
F	10.6325748	11.3380496	11.0342899
F	9.4334968	9.4641547	5.1960047
F	7.5456027	8.2405452	3.9381935
O	11.2676577	8.0349153	7.3454599
F	6.6621127	7.1608498	6.1057485
O	8.5453573	8.391163	7.4972084
O	10.3434378	7.7355094	9.8644008

[Al(OTeF5)3(FSO2Cl)]

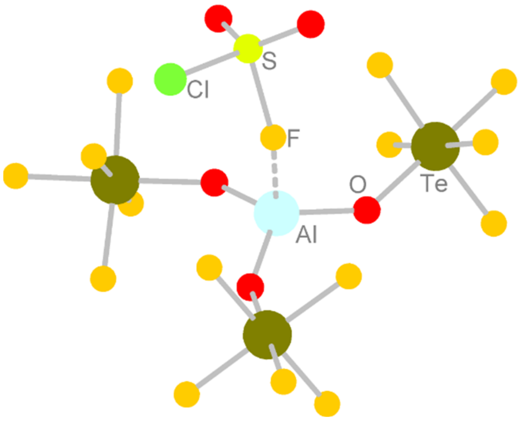

Figure S15. Representation of the B3LYP/def2-TZVPP structure of [Al(OTeF5)3(FSO2Cl)].

O	-1.5193188	0.5808734	-0.5931293
Te	-3.2575665	-0.0605561	-0.6248653
F	-2.8018669	-1.4973683	-1.7124723
Al	0.1068868	0.0816244	-0.3790791
F	2.5651854	-2.630755	-1.9881324
Te	1.6423081	-2.9298912	-0.4088936
F	0.8056013	-3.3095575	1.2164442
F	-3.8302447	1.3162311	0.476215
O	1.3090529	1.2311502	-0.7914391
Te	1.783191	2.9959281	-0.4834915
F	3.2505296	2.4775531	0.5271401
O	0.4504497	-1.5557189	-0.7511884
F	-4.9974184	-0.7004349	-0.6211969
F	-3.669395	0.951174	-2.1189389
F	-2.9372605	-1.1196182	0.8862975
F	2.2580262	4.7556261	-0.1482299
F	0.7490187	3.0423277	1.0789561
F	2.83228	3.0347478	-2.0067491
F	0.3337883	3.6410526	-1.4487293
F	2.8195107	-4.3143306	-0.0435576
F	2.8046623	-1.7857477	0.4985474
F	0.5441017	-4.1509568	-1.2607171
F	0.2828311	0.0142367	1.5595825
O	-1.4516186	1.2542576	2.8427759
Cl	-0.8645408	-1.3812751	3.6533607
S	-0.3299012	0.4538685	3.1345301
O	0.8404477	0.8527882	3.8088774

[Al(OTeF5)3(PhF)]

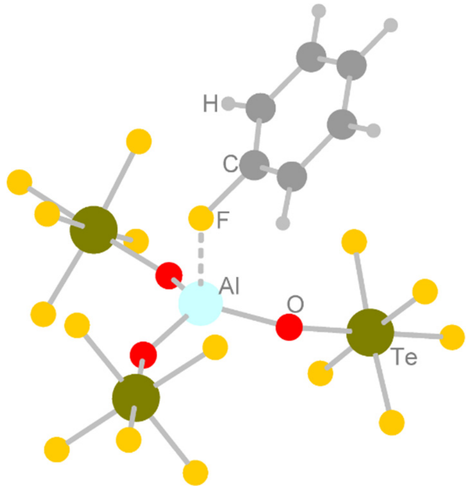

Figure S16. Representation of the B3LYP/def2-TZVPP structure of [Al(OTeF5)3(PhF)].

O	-1.3574532	-0.2727724	0.588358
Te	-2.3136574	-1.5139294	1.5758084
F	-2.3109655	-2.7493432	0.1859088
Al	-0.0032917	-0.1012996	-0.4551293
F	1.6989851	-2.2610785	-3.2578369
Te	2.3829898	-2.2493572	-1.5343431
F	3.188199	-2.267195	0.151038
F	-2.3684339	-0.3625722	3.0322396
O	-0.1615304	1.0160851	-1.7506316
Te	-0.7765345	2.7239013	-2.1093536
F	-0.9373877	3.1128148	-0.2816502
O	0.8217235	-1.5614625	-0.8232104
F	-3.2717263	-2.7396205	2.5851355
F	-3.9257775	-0.8293364	0.9729439
F	-0.7549108	-2.2780055	2.257861
F	-1.3812043	4.4424876	-2.4558129
F	-2.5482459	2.1692529	-2.1412262
F	0.9473537	3.4160796	-2.0747304
F	-0.638824	2.4454042	-3.9330973
F	3.9619877	-2.945266	-2.2124298
F	3.0894456	-0.5453897	-1.8174806
F	1.7915884	-3.9824485	-1.2692607
F	1.2278039	0.8184905	0.6512098
H	3.0240774	-0.4770933	4.6057875
C	2.3989003	0.2197726	4.0651469
C	1.7807609	1.2737181	4.7298545
H	1.9275188	1.3951719	5.7941514
C	0.9743394	2.1722884	4.0387151
H	0.495849	2.9902414	4.5588473
C	0.7733856	2.0287296	2.6677074
H	0.1564559	2.7055868	2.0951526
C	1.4077715	0.9658405	2.0793411
C	2.2178443	0.0469896	2.6941111
H	2.6814633	-0.7589149	2.1439448

[Al(OTeF$_5$)$_3$(PhF)$_2$]

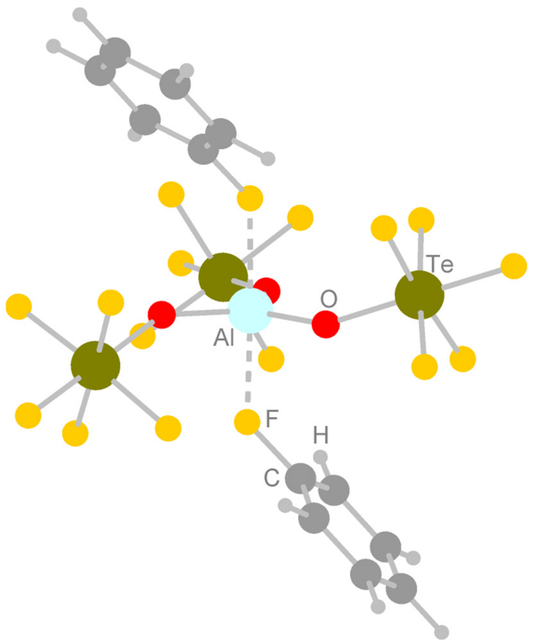

Figure S17. Representation of the B3LYP/def2-TZVPP structure of [Al(OTeF$_5$)$_3$(PhF)$_2$].

Te	-0.9734327	0.9462411	-3.1501031
Te	1.1588097	2.0805517	2.365333
Te	-0.1716671	-3.3255445	0.9736657
Al	-0.0505676	-0.0654089	-0.0117749
F	-1.8074049	1.1457159	-4.7968066
F	0.1381309	2.356877	-3.617127
F	0.1943361	-0.2487888	-3.9639077
F	-2.1577894	-0.4480817	-2.7994032
F	-2.215334	2.1609599	-2.4689639
F	2.1096551	3.3872579	3.2796951
F	-0.1779762	3.3474522	2.10866
F	1.9978518	2.6722144	0.8024259
F	2.5847001	0.9318164	2.6822111
F	0.3909529	1.592675	3.9811246
F	-0.1055044	-4.890403	1.969733
F	-1.3808765	-4.1711971	-0.1477227
F	-1.5734907	-2.7736472	2.0765791
F	1.0482762	-2.5968459	2.1787109
F	1.2390547	-3.9990641	-0.0402009
F	-2.096405	0.1182679	0.2862501
F	1.9942933	-0.2453798	-0.2465606
O	-0.1349723	0.7832676	-1.517932
O	0.195919	0.78849	1.4716042
O	-0.235291	-1.7883062	-0.0358824
C	-2.9250787	0.8317135	1.1797317
C	-3.406513	2.0484468	0.7583954
H	-3.1453827	2.4429387	-0.2122456
C	-4.2408862	2.7323455	1.638961
H	-4.6404916	3.6941831	1.3487624
C	-4.5560334	2.1856429	2.8783852
H	-5.2031727	2.7257043	3.5557318
C	-4.0422329	0.9479449	3.2516025
H	-4.2868432	0.5231659	4.2153401
C	-3.2059738	0.243024	2.389966
H	-2.7919319	-0.720134	2.648328
C	2.8611158	-0.3508289	-1.3570954
C	3.3926236	0.8158742	-1.8533997
H	3.137729	1.7700117	-1.4166834
C	4.2624742	0.6984748	-2.9341551
H	4.7015231	1.5905602	-3.3589274
C	4.5624282	-0.5526039	-3.4637006
H	5.2378994	-0.6328039	-4.3042979
C	3.9977452	-1.7006753	-2.9184216
H	4.2316227	-2.6728485	-3.3298391
C	3.1243426	-1.6118172	-1.8371641
H	2.6716644	-2.4847363	-1.3906959

[Al(OTeF$_5$)$_3$(MeCN)]

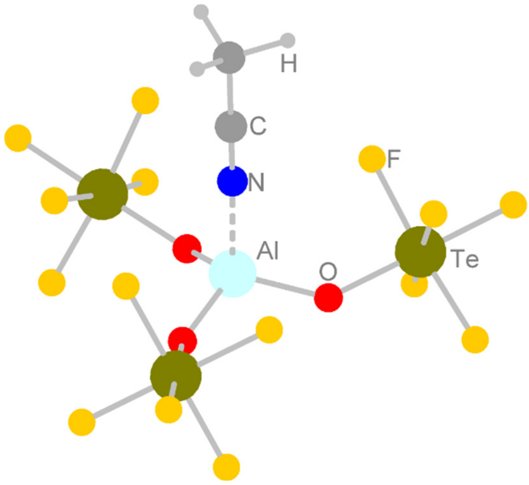

Figure S18. Representation of the B3LYP/def2-TZVPP structure of [Al(OTeF$_5$)$_3$(MeCN)].

O	-1.9648925	-0.819232	-0.1963196
Te	-2.8152015	-1.605493	1.2391308
F	-3.1169665	-3.1846733	0.3148771
Al	-0.3643834	-0.3756579	-0.6925619
F	2.3501423	-2.0246658	-3.1150025
Te	2.4081239	-2.1796014	-1.2673118
F	2.6279762	-2.3389649	0.580413
F	-2.5520314	-0.0922556	2.3078174
O	-0.3451833	0.8473366	-1.919866
Te	-0.6752961	2.6509055	-2.1189549
F	-1.7163815	2.7098639	-0.5654777
O	0.6253848	-1.7421657	-1.0823492
F	-3.6652843	-2.3764799	2.6983678
F	-4.4589891	-0.9483387	0.6971033
F	-1.209306	-2.2923	1.9124664
F	-0.9866048	4.4715375	-2.3057262
F	-2.1922714	2.3520652	-3.1420898
F	0.8105801	3.106401	-1.0765928
F	0.3661446	2.7287768	-3.6476274
F	4.2051797	-2.6172791	-1.429428
F	2.9379729	-0.3876776	-1.1259186
F	2.0120457	-3.9844471	-1.3844527
N	0.4442654	0.4397231	0.8664418
C	0.9049847	0.9357394	1.7870865
C	1.4841156	1.5637457	2.9486961
H	0.7520226	1.5706505	3.7568249
H	2.3674173	1.0050429	3.2594529
H	1.7664362	2.5874441	2.7010009

[Al(OTeF$_5$)$_3$(MeCN)$_3$]

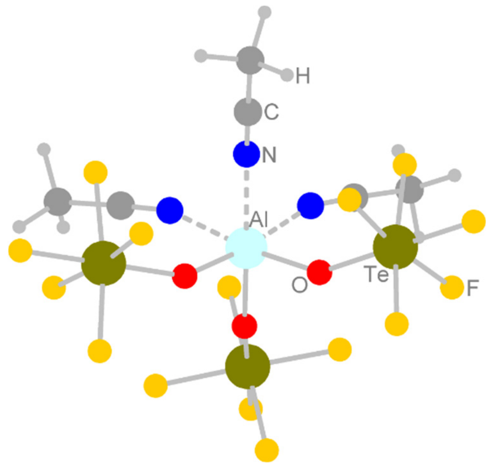

Figure S19. Representation of the B3LYP/def2-TZVPP structure of $[Al(OTeF_5)_3(MeCN)_3]$.

O	-1.0640556	-0.2607397	1.1095797
Te	-0.849383	-0.1452905	2.9076246
F	-1.9861514	-1.5888467	3.2493621
Al	-0.3461082	-0.701894	-0.5143833
F	0.3835034	1.2723515	2.8166471
O	-0.4951934	0.9824172	-1.2128612
Te	0.20802	2.2837552	-2.2635914
F	0.7353726	3.3332967	-0.809124
N	0.624424	-1.3276593	-2.2301677
F	-0.640189	-0.0264408	4.759129
F	-2.2527713	1.0547745	3.1000648
F	0.6145163	-1.3378526	2.9703635
F	0.9215319	3.6310745	-3.3411803
F	-1.40436	3.1951348	-2.3867273
F	1.9450309	1.5420415	-2.3012686
F	-0.151296	1.3769897	-3.8717283
N	1.5389765	-0.2170647	0.1820627
O	-1.9242142	-1.2970743	-1.2225892
N	0.052026	-2.5971743	0.208893
F	-2.9671409	-3.6282797	-0.3059966
Te	-2.8801777	-2.6710945	-1.9229131
F	-3.8706929	-4.0782252	-2.6473168
F	-1.3638834	-3.6988146	-2.3851893
F	-2.866931	-1.9500405	-3.6467899
F	-4.509115	-1.8590809	-1.5573648
C	1.2121331	-1.7050119	-4.7225466
C	0.900926	-1.5074549	-3.3247687
C	3.4771132	1.0706365	1.3152282
C	2.4099636	0.3350213	0.6755461
C	-0.0540543	-3.6140932	0.7203294
C	-0.238567	-4.8888457	1.3763112
H	-1.2419218	-5.2516332	1.1511955
H	-0.1331099	-4.7596786	2.4533704
H	0.4980114	-5.6078694	1.0195791
H	4.2894222	0.3979699	1.588086
H	3.0795809	1.5456865	2.2126409
H	3.8477358	1.8360935	0.6336539
H	2.2688068	-1.9403868	-4.8446011
H	0.9782259	-0.7870354	-5.2625726
H	0.6070459	-2.5215021	-5.1162964

$[Al(OTeF_5)_3(PhCN)]$

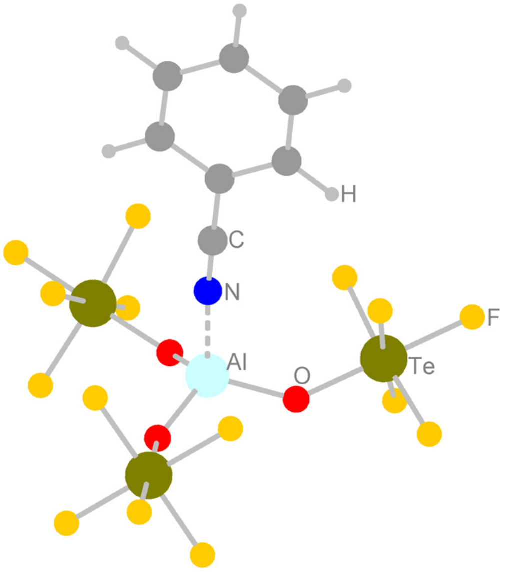

Figure S20. Representation of the B3LYP/def2-TZVPP structure of $[Al(OTeF_5)_3(PhCN)]$.

O	-1.2234647	-0.9538218	-1.0841076
Te	-1.8353611	-2.6696911	-0.8125232
F	-1.4174828	-3.1922031	-2.5416187
Al	0.0446908	0.1114682	-0.556472
F	3.4749067	1.3685022	-2.1804378
Te	3.3782311	-0.0006204	-0.93119
F	3.4347956	-1.3305693	0.3777995
F	-2.2893468	-2.3059391	0.9713101
O	-0.3552764	1.785369	-0.7686536
Te	-1.5265673	3.0445697	-0.1024163
F	-2.4942532	1.7553361	0.8514071
O	1.5850397	-0.2896077	-1.2472996
F	-2.4637605	-4.3925654	-0.5157049
F	-3.5489259	-2.2060794	-1.3417929
F	-0.1676651	-3.2642145	-0.2104056
F	-2.6946368	4.3149725	0.5842378
F	-2.6875839	2.8605159	-1.5390326
F	-0.4751142	3.3202482	1.4154219
F	-0.6378315	4.4156832	-0.975066
F	5.1840082	0.2785932	-0.5966958
F	3.0185059	1.2489983	0.4190214
F	3.86525	-1.2374121	-2.221012
N	0.1897492	-0.1987734	1.3315104
C	0.2157753	-0.4431453	2.453263
H	1.9929206	-0.1185017	6.6478648
C	1.231364	-0.5303752	6.0006919
C	0.2551476	-1.378908	6.5181204
H	0.2637664	-1.6230314	7.5719726
C	-0.7314406	-1.9171296	5.6952411
H	-1.4840053	-2.5750667	6.1066038
C	-0.7525761	-1.6119125	4.3449751
H	-1.5088375	-2.0203712	3.6893288
C	0.2335514	-0.756057	3.8306703
C	1.2302048	-0.2111243	4.6537364
H	1.9803294	0.4447802	4.23519

[Al(OTeF$_5$)$_3$(PhCN)$_3$]

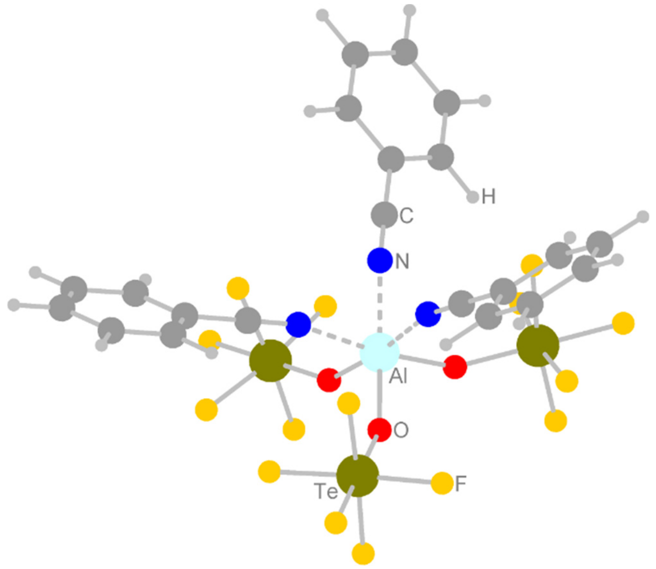

Figure S21. Representation of the B3LYP/def2-TZVPP structure of [Al(OTeF$_5$)$_3$(PhCN)$_3$].

O	-0.9967725	-0.8011577	1.342996
Te	-1.036157	-1.5046955	3.0139367
F	-1.2124912	-3.2601032	2.3969566
Al	-0.0130089	-0.0009824	0.0184608
F	-0.7861424	0.1407463	3.8780051
O	-0.973995	1.5572027	0.0513056
Te	-1.0117088	3.3504808	-0.218925
F	-1.2712596	3.7299673	1.5921067
N	1.2908903	0.7745955	-1.3834211
F	-1.0806748	-2.2417407	4.7316621
F	-2.8801219	-1.3367122	3.1581491
F	0.8358166	-1.7827765	3.121157
F	-1.052236	5.2003803	-0.4813803
F	-2.8458905	3.3777909	-0.5135634
F	0.8477638	3.5874017	0.0466072
F	-0.6922628	3.2345127	-2.0634972
N	1.2747522	0.8096097	1.4047419
O	-0.9823785	-0.7312416	-1.3567627
N	1.2550107	-1.621147	0.0407064
F	-1.0253643	-3.410422	-1.7763035
Te	-1.0380023	-1.8376799	-2.7912942
F	-1.1065865	-2.9558265	-4.2874016
F	0.8396311	-1.9857566	-2.9654539
F	-0.9809657	-0.4224626	-4.0233357
F	-2.8942807	-1.8406685	-2.8471885
H	3.9701945	2.7898058	-2.3572492
C	3.5320876	2.6222479	-3.3309684
C	2.3818816	1.8330095	-3.4538151
C	1.7905778	1.2514792	-2.2994806
C	1.7887671	1.6124921	-4.7037961
H	0.8958353	1.0078282	-4.7760954
C	2.3601678	2.1860606	-5.8289729
H	1.9081398	2.0242446	-6.7977284
C	3.5050234	2.9688963	-5.71218
H	3.9430592	3.4147787	-6.5952831
C	4.0889529	3.1868918	-4.4669612
H	4.9753989	3.8001947	-4.3818658
H	2.4687891	0.3563607	4.6264663
C	2.6540219	1.4180139	4.5454262
C	2.3220139	2.0952546	3.3653378
C	1.7480525	1.3782454	2.2813032
C	2.5260274	3.4757698	3.2421858
C	3.0734918	4.1721661	4.3080328
C	3.4111636	3.5029082	5.4812132
H	3.8342998	4.0541888	6.3104507
C	3.2006202	2.1316705	5.5999418
H	3.4553973	1.6198586	6.5176578
H	2.2417353	-4.0632451	2.2689306
C	2.5270041	-4.5741802	1.3601233
H	3.2443351	-6.330947	2.3459136
C	3.0839311	-5.8426998	1.3946147
C	2.3184455	-3.9569329	0.1200735
C	1.7353821	-2.6620576	0.0749753
C	3.4264448	-6.4883322	0.2095764
C	2.6558653	-4.6038399	-1.0755507
C	3.2117107	-5.8720452	-1.0203282
H	3.8571559	-7.4802348	0.2444008
H	2.4680125	-4.11613	-2.0216262
H	3.4710441	-6.3830187	-1.9372402
H	2.2433488	3.9806138	2.3291431
H	3.2297153	5.2389302	4.2262016

[Al(OTeF$_5$)$_3$(Et$_2$O)]

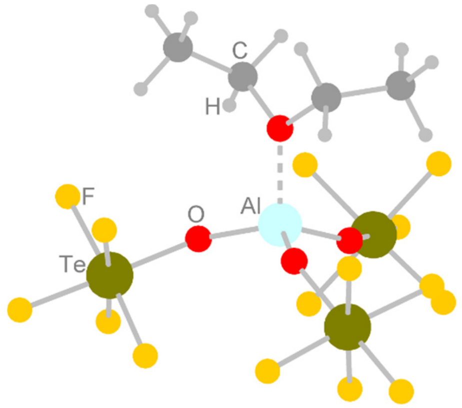

Figure S22. Representation of the B3LYP/def2-TZVPP structure of [Al(OTeF$_5$)$_3$(Et$_2$O)].

Te	0.3475401	0.8101471	-3.1991144
Te	0.7034086	-3.1163599	0.8561381
Te	-0.0651754	2.335533	2.0068034
Al	-0.7125426	-0.2118943	-0.1375418
F	0.0278413	-0.9790218	-3.6218115
F	1.9550408	0.3375451	-2.3922277
F	1.3246995	1.7169103	3.0631108
F	0.2262317	4.0266792	2.711144
F	1.2606477	0.9497186	-4.8076486
F	0.7083898	2.6043281	-2.9087114
F	-1.2070878	1.2740549	-4.1112211
F	1.1320757	2.7605944	0.6495497
F	-0.5192788	-4.2942646	0.087871
F	-1.4618309	3.0745506	1.0085492
F	-0.5650718	-2.7168719	2.1794954
F	1.9776428	-3.6250991	-0.3868861
F	-1.259538	2.0341672	3.4032966
F	1.9477214	-2.0500643	1.7303759
O	-0.3868217	0.6480265	1.3298651
O	-2.5384816	-0.5663934	-0.0499073
O	-0.599507	0.6770221	-1.6178971
F	1.2612295	-4.5238266	1.9294087
O	0.114757	-1.736438	-0.2167504
C	-3.3430785	-0.8403366	-1.259362
H	-2.9863988	-0.1336884	-2.003147
H	-4.3684553	-0.5831692	-1.0047289
C	-3.2246111	-0.812627	1.2363402
H	-2.4305502	-0.9559355	1.9636096
H	-3.7608501	-1.7536769	1.1260334
C	-4.1290954	0.3367015	1.6152982
H	-4.583776	0.1128592	2.5817013
H	-4.9356506	0.4842936	0.8974624
H	-3.5696983	1.2652188	1.712919
C	-3.2035595	-2.2743352	-1.716807
H	-2.1762002	-2.5098727	-1.9905712
H	-3.8282042	-2.4175101	-2.6001547
H	-3.5328911	-2.9814941	-0.955754

[Al(OTeF$_5$)$_3$(Et$_2$O)$_2$]

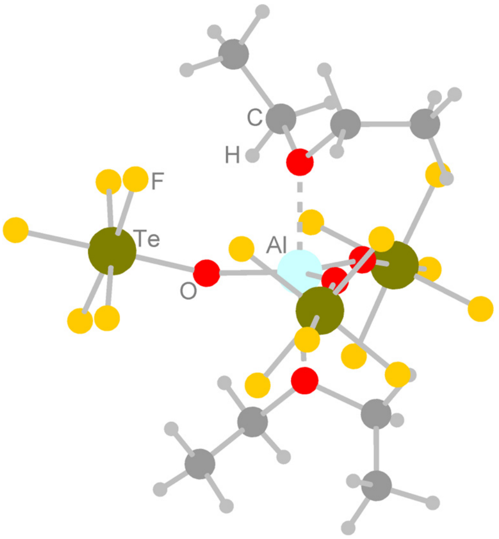

Figure S23. Representation of the B3LYP/def2-TZVPP structure of [Al(OTeF$_5$)$_3$(Et$_2$O)$_2$].

Te	5.5979325	3.7347362	5.253538
Te	10.8903802	0.5946733	2.8222348
Te	7.9634559	5.5953248	1.002465
Al	8.3605305	2.9989786	3.3068593
F	5.095431	2.0146883	5.7681768
F	4.7569865	3.4807015	3.6085595
F	7.7857804	4.7131725	-0.6262003
F	7.6725375	7.1819627	0.0796749
F	3.9578698	4.31717	5.9031002
F	5.9626382	5.5045963	4.8038082
F	6.2827848	4.0338304	6.9597225
F	6.1220484	5.498438	1.2564502
F	12.1253763	1.1047429	4.1203782
F	8.1203077	6.6231005	2.549833
O	7.1468328	1.6203354	2.4383088
F	11.6350504	1.8255199	1.633286
F	10.2600024	-0.7539274	3.9413868
F	9.7813107	5.8393394	0.6665305
F	9.7836728	-0.0362669	1.451969
O	8.2671846	4.0054308	1.8658314
O	9.6162527	4.3151078	4.2370598
O	7.2332993	3.1474271	4.6610647
F	12.1621469	-0.6203452	2.221443
O	9.6537171	1.7864687	3.445241
C	9.5922581	4.5733732	5.6756169
H	8.5578982	4.781246	5.9176035
H	10.1600766	5.4847344	5.8444767
C	6.9915991	1.4768668	0.9861611
H	7.1042586	0.4181291	0.7564679
H	7.8268065	2.0050532	0.537619
C	10.9239052	4.540222	3.6172378
H	10.8096268	4.2095079	2.5900727
H	11.6473835	3.8857463	4.1028207
C	6.8929788	0.3924879	3.1926144
H	7.1482783	0.6335321	4.2202732
H	7.5907549	-0.3635133	2.8325938
C	5.6731123	2.0307899	0.4876799
H	5.6609595	1.9712719	-0.6023825
H	5.550023	3.0741571	0.7685513
H	4.8209702	1.47206	0.8687751
C	11.3701376	5.9896736	3.6488777
H	12.2484587	6.0843747	3.0084717

H	11.6587335	6.3251859	4.6439407
H	10.5983459	6.6518318	3.2619801
C	5.4624757	-0.0996573	3.1084983
H	5.2012954	-0.446345	2.1096241
H	4.7543665	0.6667273	3.416794
H	5.3584941	-0.9483146	3.7869387
C	10.1311182	3.4231487	6.5027592
H	9.5353417	2.5221017	6.3665108
H	10.0833056	3.701333	7.557028
H	11.1692391	3.1915309	6.266232

$[Al(OTeF_5)_2Me]_2$

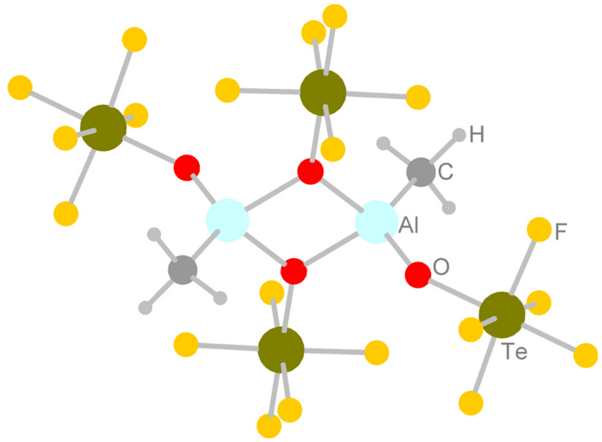

Figure S24. Representation of the BP86/def-SV(P) structure of $[Al(OTeF_5)_2Me]_2$.

C	-1.6594461	2.4951904	-0.2564480
O	1.5633360	2.0826647	0.0429539
Te	2.5913295	3.6162917	-0.0496177
F	3.6359734	5.1463628	-0.1412347
F	1.9175632	4.2554877	1.5631690
F	3.3315847	3.0922308	-1.6712519
F	1.2608646	4.5503122	-0.9646559
F	3.9810759	2.7867924	0.8555633
O	-1.5529453	-2.0755860	-0.0426786
Te	-2.6046279	-3.5938947	0.0244642
F	-3.6748097	-5.1074942	0.0898235
F	-3.3692604	-3.0637335	1.6325739
F	-1.9084284	-4.2391262	-1.5764395
F	-1.3061709	-4.5498237	0.9621607
F	-3.9625129	-2.7399486	-0.9060260
C	1.6658525	-2.5095077	0.2697993
Al	-0.0500286	1.4551178	-0.0700496
O	-0.0956252	0.0566541	1.2154535
Te	-0.2803230	0.1401124	3.1103740
F	-0.4576010	0.2154419	4.9434755
F	1.5557252	0.1266984	3.3271239
F	-2.1232239	0.1582281	2.9687961
F	-0.2949972	-1.7146013	3.1573173
F	-0.2592965	1.9913648	3.0049451
Al	0.0631227	-1.4601494	0.0796996
O	0.1155026	-0.0613456	-1.2042942
Te	0.2921264	-0.1476201	-3.0998833
F	0.4615995	-0.2267897	-4.9335454
F	2.1354030	-0.1777490	-2.9652886
F	-1.5446825	-0.1228424	-3.3091514
F	0.3180921	1.7067210	-3.1493442
F	0.2599767	-1.9986187	-2.9918586
H	-1.6234794	3.1141322	-1.1546550
H	-1.7944831	3.1673777	0.5930315
H	-2.5446756	1.8599131	-0.3221271
H	1.8090677	-3.1684762	-0.5886766
H	1.6163971	-3.1426779	1.1574149
H	2.5528449	-1.8792791	0.3567467

Al(C_6F_5)$_3$

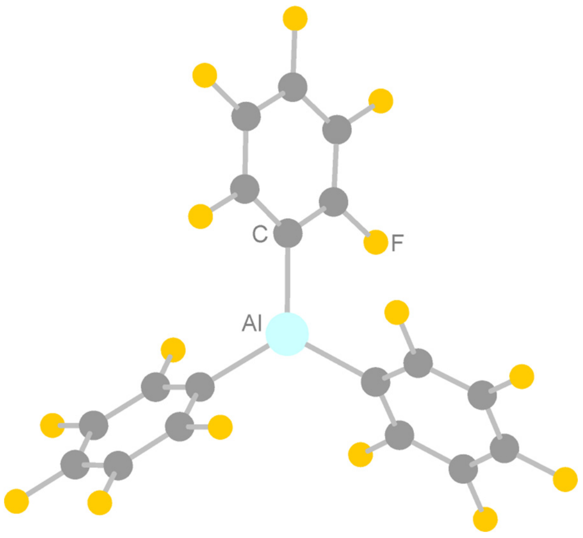

Figure S25. Representation of the BP86/def-SV(P) structure of Al(C_6F_5)$_3$.

C	2,5581884	0,7360921	1,4532835
C	1,3052162	0,5627493	2,0555348
F	1,0934578	1,0149188	3,3138883
C	0,2201274	-0,0736178	1,4285962
F	-0,9705738	-0,2179110	2,0424568
C	0,3907904	-0,5688283	0,1252515
F	-0,6283173	-1,1845399	-0,5007484
C	1,6294353	-0,4276945	-0,5196103
F	1,7877871	-0,9137707	-1,7666358
C	2,6788352	0,2225073	0,1568763
F	3,8482348	0,3179185	-0,5203053
F	4,2866830	3,6618397	0,0221924
F	6,3734296	4,3573390	-1,5699947
C	5,4788522	3,0616003	0,2470019
C	6,5370803	3,4388986	-0,5980450
C	5,6042468	2,1190731	1,2753006
C	7,7921122	2,8335691	-0,4196565
F	8,8252831	3,1728330	-1,2119837
C	6,8698635	1,5365210	1,4048705
C	7,9657864	1,8718262	0,5881643
F	7,1004219	0,5882780	2,3445806
F	9,1701530	1,2891833	0,7497087
F	3,3525059	-1,2836461	3,7446942
F	4,2580965	-2,4410787	6,0185718
C	4,2089303	-0,5666565	4,5113171
C	4,6665648	-1,2011827	5,6823507
C	4,6093570	0,7177855	4,1266768
C	5,5700260	-0,5217177	6,5154829
F	6,0212902	-1,1052578	7,6408805
C	5,5172872	1,3490824	4,9867755
C	6,0078058	0,7673987	6,1680698
F	5,9798135	2,5865771	4,6867840
F	6,8820464	1,4109489	6,9664626
Al	4,0001884	1,7482251	2,4769679
F	3,3409944	3,2415666	2,9911094

[Al(C_6F_5)$_3$F]$^-$

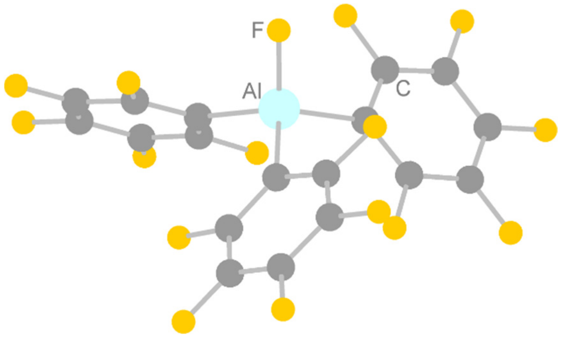

Figure S26. Representation of the BP86/def-SV(P) structure of [Al(C_6F_5)$_3$F]$^-$.

C	2,5581884	0,7360921	1,4532835
C	1,3052162	0,5627493	2,0555348
F	1,0934578	1,0149188	3,3138883
C	0,2201274	-0,0736178	1,4285962
F	-0,9705738	-0,2179110	2,0424568
C	0,3907904	-0,5688283	0,1252515
F	-0,6283173	-1,1845399	-0,5007484
C	1,6294353	-0,4276945	-0,5196103
F	1,7877871	-0,9137707	-1,7666358
C	2,6788352	0,2225073	0,1568763
F	3,8482348	0,3179185	-0,5203053
F	4,2866830	3,6618397	0,0221924
F	6,3734296	4,3573390	-1,5699947
C	5,4788522	3,0616003	0,2470019
C	6,5370803	3,4388986	-0,5980450
C	5,6042468	2,1190731	1,2753006
C	7,7921122	2,8335691	-0,4196565
F	8,8252831	3,1728330	-1,2119837
C	6,8698635	1,5365210	1,4048705
C	7,9657864	1,8718262	0,5881643
F	7,1004219	0,5882780	2,3445806
F	9,1701530	1,2891833	0,7497087
F	3,3525059	-1,2836461	3,7446942
F	4,2580965	-2,4410787	6,0185718
C	4,2089303	-0,5666565	4,5113171
C	4,6665648	-1,2011827	5,6823507
C	4,6093570	0,7177855	4,1266768
C	5,5700260	-0,5217177	6,5154829
F	6,0212902	-1,1052578	7,6408805
C	5,5172872	1,3490824	4,9867755
C	6,0078058	0,7673987	6,1680698
F	5,9798135	2,5865771	4,6867840
F	6,8820464	1,4109489	6,9664626
Al	4,0001884	1,7482251	2,4769679
F	3,3409944	3,2415666	2,9911094

Al[N(C_6F_5)$_2$]$_3$

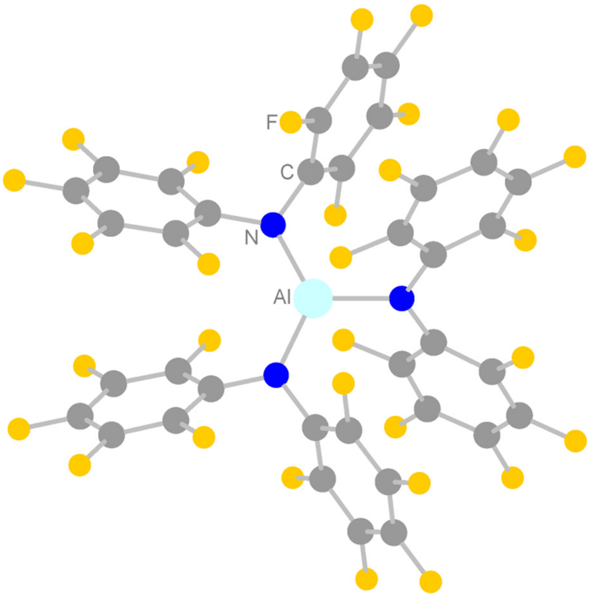

Figure S27. Representation of the BP86/def-SV(P) structure of Al[N(C_6F_5)$_2$]$_3$.

F	0.5581502	2.1025306	-0.8730444
F	1.6608077	3.8824144	-2.6987592
C	1.8122236	1.8965897	-1.3984916
C	2.3367464	2.7959470	-2.3287447
C	2.4703684	0.7322748	-0.9512876
C	3.6012637	2.5113993	-2.8807607
F	4.1467514	3.3421252	-3.7714817
C	3.7134731	0.4502163	-1.5643278
C	4.2889761	1.3502554	-2.4800403
F	4.3712424	-0.6821499	-1.2775831
F	5.4784684	1.0727835	-3.0178087
F	0.4657042	-2.2245495	0.8147397
F	1.5059771	-4.0242284	2.6328932
C	1.7141603	-2.0481382	1.3285438
C	2.2176386	-2.9600652	2.2614027
C	2.4212781	-0.9103603	0.8837976
C	3.4884113	-2.7194765	2.8184152
F	4.0033956	-3.5698372	3.7089690
C	3.6696604	-0.6681244	1.5028912
C	4.2169956	-1.5838284	2.4193063
F	4.3621925	0.4452764	1.2184205
F	5.4133909	-1.3429422	2.9608146
N	1.7764029	-0.0677919	-0.0347734
F	-2.6168003	-2.2557531	-2.9384335
F	1.7957075	-3.5891447	-3.9803670
F	0.2666286	-2.9247468	-1.8368633
C	1.2242575	-2.3830700	-3.9203115
C	0.4344880	-2.0274316	-2.8129267
F	-5.2410518	-1.7645748	-3.4188533
F	2.1293800	-1.8139013	-6.0403609
C	1.3916348	-1.4759932	-4.9817518
C	-3.1471674	-1.0995819	-2.5167412
C	-0.1794804	-0.7588162	-2.7217245
N	-0.9625877	-0.3744558	-1.5933469
C	0.7820641	-0.2091564	-4.9232176
C	-4.5084026	-0.8570439	-2.7676014
C	0.0181863	0.1385653	-3.7975521
F	0.9541248	0.6614575	-5.9225964
C	-2.3388535	-0.1577089	-1.8361421
F	-0.5288903	1.3617835	-3.7423841
C	-5.1123375	0.3260525	-2.3038715
F	-6.4103688	0.5479774	-2.5224857
C	-2.9753818	1.0230773	-1.3945417
C	-4.3403682	1.2704385	-1.6040493
F	-2.2435050	1.9459058	-0.7350158
F	-4.8990601	2.4015255	-1.1635524
F	-0.5739100	-1.3348498	3.7376146
F	-5.0126525	-2.0492314	1.3696762
F	-2.3533113	-1.8087490	0.8325309
C	-4.3540729	-0.9530901	1.7576356
C	-2.9820255	-0.8180741	1.4922703
F	1.0105390	-0.7892410	5.8890574
F	-6.3286073	-0.0567983	2.7299583
C	-5.0271535	0.0628811	2.4588649
C	0.0983421	-0.1758837	3.7786910
C	-2.2475122	0.3239023	1.8806413
N	-0.8652941	0.4421308	1.5928915
C	-4.3223172	1.2099760	2.8675430
C	0.9147821	0.0923433	4.8892645
C	-2.9571138	1.3406295	2.5631538
F	-4.9620352	2.1856893	3.5183666
C	-0.0236359	0.7366565	2.7042251
F	-2.3233355	2.4616905	2.9330040
C	1.6589186	1.2858471	4.9319001
F	2.4493372	1.5436835	5.9750249
C	0.7264382	1.9307878	2.7799029
C	1.5714942	2.2028631	3.8694214
F	0.6422589	2.8358414	1.7965699
F	2.2700136	3.3408720	3.9132065
Al	-0.0970676	0.0642809	-0.0308633

$[Al\{N(C_6F_5)_2\}_3F]^-$

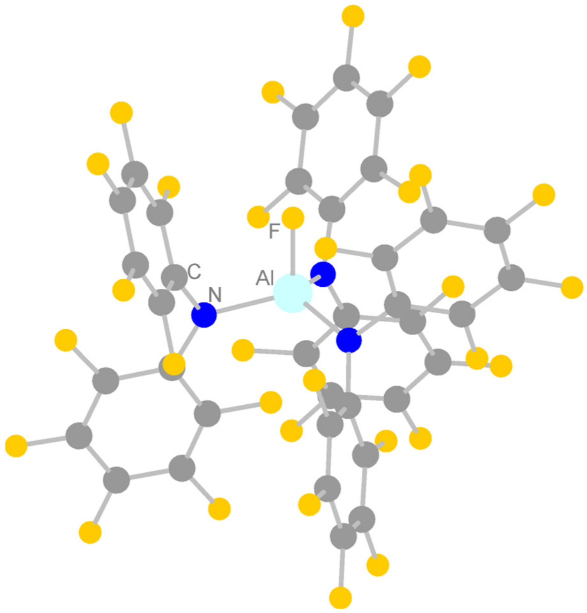

Figure S28. Representation of the BP86/def-SV(P) structure of $[Al\{N(C_6F_5)_2\}_3F]^-$.

F	-0.2077752	5.0627920	2.1555365
F	-0.1951305	7.3279805	0.6719352
C	0.5061456	5.0749856	1.0243455
C	0.5103756	6.2597356	0.2668382
C	1.2194087	3.9218863	0.6116143
C	1.2915579	6.3490118	-0.8969427
F	1.3094498	7.4832071	-1.6135845
C	2.0042070	4.0533911	-0.5625209
C	2.0430521	5.2380736	-1.3137115
F	2.7291920	3.0117917	-0.9902438
F	2.7873028	5.3144811	-2.4289001
F	2.9684365	0.6075918	0.4696129
F	5.1660542	-0.0644076	1.8991061
C	3.2491760	1.3142679	1.5656838
C	4.3994909	0.9652088	2.2943806
C	2.4160906	2.3844866	1.9725271
C	4.7838684	1.7198817	3.4137367
F	5.8922987	1.3974092	4.0987996
C	2.8368864	3.1289079	3.1005935
C	3.9985924	2.8110365	3.8180563
F	2.0950753	4.1640986	3.5213922
F	4.3576603	3.5324300	4.8932992
N	1.1967385	2.7017106	1.3213255
F	-2.5693465	0.3583429	-2.6923254
F	0.0428172	-3.6281856	-0.8843890
F	-1.3231975	-1.6769790	0.4342271
C	0.1479277	-2.3487871	-1.2779168
C	-0.5424097	-1.3352237	-0.5948658
F	-4.4304197	2.0406970	-3.6394696
F	1.5878459	-3.0015874	-3.0565712
C	0.9382996	-2.0313274	-2.3951952
C	-2.7715248	1.5136264	-2.0257104
C	-0.4400807	0.0163018	-0.9902548
N	-1.0563987	1.0496306	-0.2429840
C	1.0373415	-0.6971832	-2.8234955
C	-3.7565346	2.3816766	-2.5283788
C	0.3605480	0.3066126	-2.1157744
F	1.7857815	-0.3913013	-3.8954381
C	-2.0282803	1.8203576	-0.8515809
F	0.4700904	1.5778851	-2.5351188
C	-4.0618688	3.5913375	-1.8832268
F	-5.0104171	4.4097400	-2.3707203
C	-2.3765398	3.0504614	-0.2467582
C	-3.3615472	3.9222384	-0.7105890
F	-1.6889680	3.3731080	0.8822073
F	-3.6272616	5.0615863	-0.0574572
F	-3.4493203	-0.1073429	3.7449741
F	-5.0320696	4.3653191	3.4427317
F	-3.7693071	2.2920786	2.2341808
C	-3.8247203	3.9718361	3.8788498
C	-3.1591212	2.8993656	3.2607667
F	-3.3325763	-2.3409473	5.2921078
F	-3.8877136	5.6204374	5.5959035
C	-3.2468681	4.6103593	4.9883713
C	-2.2565443	-0.4992800	4.2198564
C	-1.8837286	2.4642203	3.6906869
N	-1.1849122	1.4000044	3.0771923
C	-1.9881931	4.1933778	5.4522266
C	-2.2095622	-1.6572819	5.0114151
C	-1.3289145	3.1352778	4.8085951
F	-1.4254451	4.8114925	6.5029582
C	-1.0964411	0.2453889	3.8954436
F	-0.1287188	2.7606988	5.2709915
C	-0.9763973	-2.1105186	5.5049915
F	-0.9190302	-3.2201816	6.2586639
C	0.1267751	-0.2225236	4.4348126
C	0.1951648	-1.3994862	5.1981606
F	1.2617040	0.4547853	4.2342023
F	1.3720785	-1.8259990	5.6839549
Al	-0.1813853	1.3938116	1.4585795
F	0.6505258	-0.0895985	1.6484193

Al[OC(C_6F_5)$_3$]$_3$

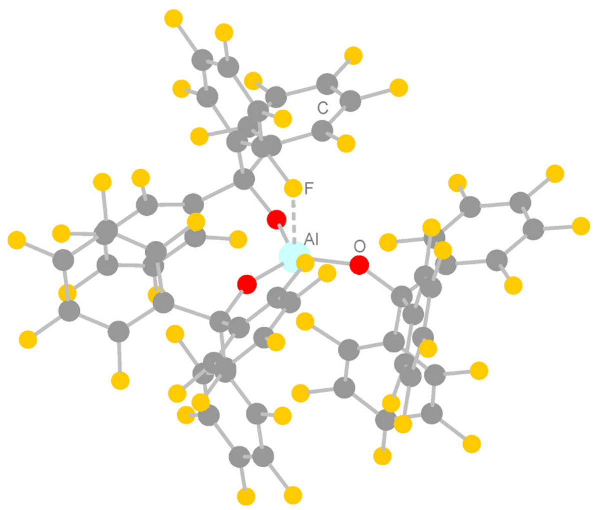

Figure S29. Representation of the BP86/def-SV(P) structure of Al[OC(C_6F_5)$_3$]$_3$.

C	-0.0116658	1.3478711	-3.1711144
C	-1.0458911	0.1741925	-3.0292969
C	-1.8504595	-0.1238281	-4.1524006
F	-1.7432161	0.5902719	-5.2873076
C	-2.8057230	-1.1525909	-4.1531628
F	-3.5261683	-1.3995789	-5.2514325
C	-3.0180891	-1.9047672	-2.9877140
F	-3.9220895	-2.8836968	-2.9714816
C	-2.2574491	-1.6200941	-1.8446660
F	-2.4472686	-2.3242265	-0.7253767
C	-1.3077048	-0.5817597	-1.8675512
F	-0.6499445	-0.3700478	-0.7167480
F	3.5117097	-0.9757417	-0.6457683
C	2.8523161	0.1651904	-0.8580115
C	1.8050076	0.2167875	-1.7859165
F	1.5204959	-0.9238667	-2.4405558
C	1.0528060	1.3854845	-2.0376274
C	1.4783472	2.5459225	-1.3618205
F	0.9005633	3.7429384	-1.5617054
C	2.5310128	2.5264766	-0.4251639
F	2.8867286	3.6509467	0.2017818
C	3.2318998	1.3370087	-0.1816614
F	4.2433574	1.3165202	0.6864375
F	-0.3458994	3.3303412	-5.4412181
C	-1.0598401	3.4757212	-4.3131067
C	-1.9675227	4.5545825	-4.3104969
F	-2.0668916	5.3394852	-5.3900279
C	-2.7587922	4.8172526	-3.1838325
F	-3.6127476	5.8400652	-3.1796908
C	-2.6352353	3.9882833	-2.0552882
F	-3.3681671	4.2259665	-0.9645362
C	-1.7277435	2.9206806	-2.0846224
F	-1.6262013	2.1721348	-0.9727957
C	-0.9157288	2.6230000	-3.2011433
O	0.6851588	1.1512212	-4.3511813
F	6.5292771	1.8369598	-9.0506197
F	4.9643635	1.6932155	-6.8865791
C	5.9418841	2.9480701	-8.5959814
C	5.1058151	2.8950573	-7.4697152
F	5.5148773	0.6692545	-2.3762845
F	7.4781000	2.4803409	-1.7425762
F	6.9920403	4.2532526	-10.2800631
C	5.5346229	1.8712117	-2.9577424
C	6.5393196	2.7942157	-2.6319824
C	6.1938613	4.1832408	-9.2147409
C	4.5492403	2.2259767	-3.8940748
C	6.5389169	4.0558816	-3.2531284
F	3.6026323	1.2458295	-4.1112247
O	2.6559198	2.7895794	-6.0035753
F	7.4792735	4.9496369	-2.9463401
C	4.5180263	3.4636481	-4.5515530
C	5.5424551	4.3651946	-4.1898508
C	4.4601520	4.0360525	-6.9412269
C	3.5264584	3.8229638	-5.6999376
F	5.5730656	5.5852605	-4.7445568
C	5.5964073	5.3425979	-8.6972171
F	1.8426091	5.0146536	-7.6260717
C	4.7647255	5.2633936	-7.5641207
C	2.6208697	5.0398381	-5.3692408
F	5.8358915	6.5268898	-9.2674909
C	1.7574410	5.5105764	-6.3839897
F	4.2697593	6.4274902	-7.1177516
F	3.1324162	5.1708336	-3.0178211
C	2.4214375	5.5823631	-4.0856969
C	0.8057531	6.5145909	-6.1634667
F	0.0319365	6.9432912	-7.1626944
C	1.4713097	6.5908766	-3.8349013
C	0.6474400	7.0474491	-4.8727712
F	1.3300391	7.0843753	-2.6010016
F	-0.2669130	7.9898235	-4.6412936
F	4.1587183	-3.8512380	-3.2404785
F	4.2815834	-2.2714877	-5.4084735
C	3.0332505	-3.5295145	-3.8833284
C	3.0750770	-2.7059202	-5.0169740
F	4.8330519	0.0981014	-10.7078604
F	5.8986158	-2.4141104	-11.0339003
F	1.7378225	-4.7687229	-2.3238254
C	4.4774906	-0.8947508	-9.8873149
C	5.0114418	-2.1813353	-10.0659919
C	1.7958766	-3.9996346	-3.4106535
C	3.5421420	-0.6674124	-8.8654655
C	4.5951668	-3.2197043	-9.2195679
F	3.0248100	0.5706637	-8.7951908

O	2.5441154	-0.1482990	-6.2454402
F	5.0855120	-4.4516781	-9.3848286
C	3.1202258	-1.6828957	-7.9768789
C	3.6497514	-2.9688199	-8.2074039
C	1.9183676	-2.3497929	-5.7481793
C	2.0768770	-1.2933100	-6.8772800
F	3.2869516	-4.0274502	-7.4676260
C	0.6262343	-3.6491732	-4.0970033
F	0.5959549	1.3325496	-7.1332312
C	0.6973528	-2.8244329	-5.2354899
C	0.7648409	-1.0410212	-7.6833146
F	-0.5650541	-4.0671143	-3.6494931
C	0.1435068	0.2096078	-7.8031335
F	-0.4861497	-2.5075281	-5.7985912
F	0.7031107	-3.3112712	-8.3846122
C	0.1834701	-2.0766965	-8.4463182
C	-0.9907592	0.4460329	-8.5958433
F	-1.5350938	1.6602149	-8.6551526
C	-0.9451429	-1.8870628	-9.2562664
C	-1.5342025	-0.6124360	-9.3394467
F	-1.4594682	-2.9043481	-9.9480163
F	-2.6050866	-0.4146978	-10.1052473
Al	1.9351103	1.3025761	-5.5217021

$[Al\{OC(C_6F_5)_3\}_3F]^-$

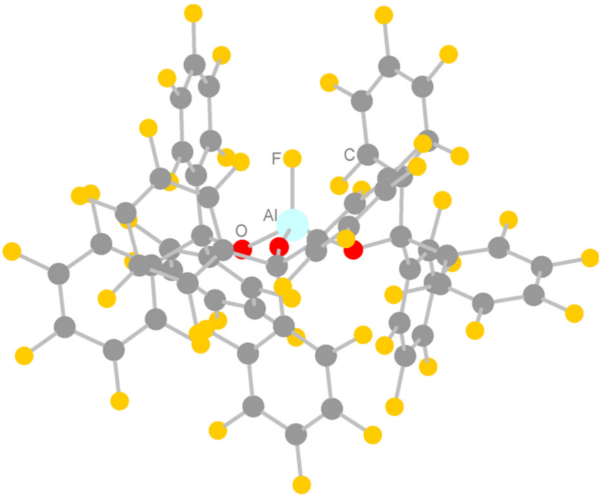

Figure S30. Representation of the BP86/def-SV(P) structure of $[Al\{OC(C_6F_5)_3\}_3F]^-$.

C	0.4168834	1.1364604	-2.4864938
C	-0.8386036	0.1842807	-2.3682325
C	-1.9313840	0.4542103	-3.2220034
F	-1.9224845	1.5191829	-4.0314276
C	-3.0878751	-0.3436070	-3.2458232
F	-4.1023136	-0.0456839	-4.0664245
C	-3.1909866	-1.4424359	-2.3790218
F	-4.2879244	-2.2095566	-2.3859356
C	-2.1387019	-1.7204650	-1.4960555
F	-2.2338209	-2.7563061	-0.6484525
C	-0.9937162	-0.9033026	-1.4873818
F	-0.0582223	-1.2253336	-0.5769918
F	3.8053235	-2.4002987	-2.0894467
C	3.3738790	-1.2075826	-1.6646393
C	2.1739739	-0.6676259	-2.1459076
F	1.4813939	-1.4127854	-3.0211542
C	1.6628309	0.5784863	-1.7191882
C	2.4331669	1.2631506	-0.7615388
F	2.0742807	2.4593473	-0.2652517
C	3.6387235	0.7399091	-0.2538242
F	4.3363915	1.4304050	0.6597199
C	4.1192662	-0.4919019	-0.7128748
F	5.2679236	-0.9894612	-0.2400754
F	0.4015900	3.8353292	-3.8399936
C	-0.1071393	3.7083019	-2.6089184
C	-0.6656020	4.8859235	-2.0704982
F	-0.6675546	6.0127659	-2.7923793
C	-1.2116746	4.8864948	-0.7805547
F	-1.7329211	6.0065687	-0.2651725
C	-1.2042857	3.6975989	-0.0333601
F	-1.7054277	3.6816387	1.2108701
C	-0.6493322	2.5403978	-0.5948854
F	-0.6303436	1.4386455	0.1783450
C	-0.0965816	2.4961877	-1.8915857
O	0.7674148	1.2220324	-3.7923394
F	7.6842861	1.2681096	-5.3240145
F	5.3037292	2.0637440	-4.4111784
C	6.9322279	2.0833668	-6.0754626
C	5.6711280	2.5016851	-5.6217253
F	3.9228598	4.8756257	-0.9025518
F	5.2448067	7.1992436	-1.5258683
F	8.6157248	2.1469121	-7.7545538
C	4.1206351	5.2078319	-2.1847733
C	4.7973323	6.3937882	-2.4975167
C	7.4154752	2.5383136	-7.3104633
C	3.6537311	4.3603898	-3.2120442
C	4.9984875	6.7309833	-3.8457131
F	3.0265034	3.2539250	-2.8013943
O	2.8446098	2.5557150	-5.3106271
F	5.6383543	7.8659019	-4.1652881
C	3.8385923	4.6696256	-4.5724758
C	4.5195083	5.8744201	-4.8456225
C	4.8329485	3.3532163	-6.3771867
C	3.4278548	3.7113343	-5.7485148
F	4.7160723	6.2585225	-6.1210815
C	6.6267456	3.4117023	-8.0701073
F	2.8110103	2.6221576	-8.2953084
C	5.3665310	3.8196344	-7.5943194
C	2.4543864	4.4046528	-6.7468808
F	7.0819573	3.8699850	-9.2459256
C	2.1567966	3.7475076	-7.9583762
F	4.7025914	4.6814334	-8.3841644
F	1.7813900	6.2171539	-5.3032234
C	1.6556812	5.5257133	-6.4510480
C	1.1950401	4.2080528	-8.8631852
F	0.9674478	3.5479689	-10.0072784
C	0.6797577	6.0106366	-7.3412522
C	0.4409978	5.3481748	-8.5507371
F	-0.0371424	7.0979308	-7.0216790
F	-0.4870343	5.8026723	-9.4009501
F	2.8858684	-4.4964551	-4.0460724
F	3.2284928	-2.1175126	-5.2286599
C	1.9856037	-4.0672622	-4.9392617
C	2.1361026	-2.8157585	-5.5604404
F	6.3600138	-0.4581329	-8.3517500
F	6.0941803	-2.2966462	-10.3685026
F	0.7436548	-6.0831163	-4.6973409
C	5.1616616	-1.0096503	-8.5990515
C	5.0338396	-1.9494465	-9.6287982
C	0.8991339	-4.8868470	-5.2785366
C	4.0463583	-0.6461539	-7.8147738
C	3.7756610	-2.5206168	-9.8785046
F	4.2893744	0.2478228	-6.8557032

O	1.8093351	-0.0304811	-6.1264876
F	3.6279689	-3.4186859	-10.8640113
C	2.7703657	-1.1978817	-8.0443466
C	2.6816191	-2.1380154	-9.0918100
C	1.2192679	-2.3236880	-6.5178021
C	1.5201392	-0.9005186	-7.1398413
F	1.4942095	-2.6987475	-9.3900661
C	-0.0156863	-4.4425541	-6.2414271
F	-1.0815077	-0.6796149	-6.0610193
C	0.1619613	-3.1909603	-6.8586299
C	0.3215796	-0.3311217	-7.9591859
F	-1.0527396	-5.2206238	-6.5868830
C	-0.9203047	-0.1915464	-7.3046489
F	-0.7482706	-2.8829596	-7.7982998
F	1.5375539	0.2372296	-9.9641414
C	0.4050220	0.2506647	-9.2380654
C	-2.0312211	0.4264257	-7.8875919
F	-3.1889885	0.5169482	-7.2217284
C	-0.6995673	0.8646587	-9.8575774
C	-1.9182384	0.9676936	-9.1771437
F	-0.5817895	1.3646271	-11.0951543
F	-2.9677987	1.5630328	-9.7572055
Al	1.3845074	1.5436317	-5.4046858
F	0.2088074	2.2984102	-6.3909330

$Al(OC_5NF_4)_3$

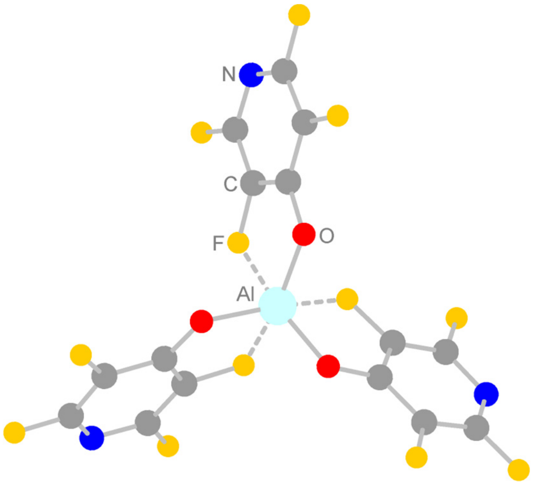

Figure S31. Representation of the BP86/def-SV(P) structure of $Al(OC_5NF_4)_3$.

Al	-0.0000000	-0.0000000	0.6712318
O	-1.5995034	-0.5245112	1.2664779
C	-2.2742252	-1.4658845	0.6214728
C	-3.5217939	-1.9892357	1.0342164
C	-4.1069367	-2.9825676	0.2260212
N	-3.5711724	-3.4518026	-0.8901935
C	-2.4062086	-2.9852894	-1.3008006
C	-1.7486380	-1.9992031	-0.5733516
F	-4.1101865	-1.5537225	2.1483305
F	-0.5187338	-1.4467841	-0.9360203
F	-1.8760628	-3.4762622	-2.4212236
F	-5.2812441	-3.4906175	0.6012680
O	1.2539917	-1.1229550	1.2664779
O	0.3455117	1.6474662	1.2664779
C	-0.1323806	2.7024791	0.6214728
C	2.4066058	-1.2365946	0.6214728
C	0.0381683	4.0445809	1.0342164
C	-0.5295109	5.0479953	0.2260212
N	-1.2037625	4.8186273	-0.8901935
C	-1.3822322	3.5764824	-1.3008006
C	-0.8570417	2.5139665	-0.5733516
C	2.6056797	-0.5147633	-0.5733516
C	3.7884407	-0.5911930	-1.3008006
N	4.7749349	-1.3668248	-0.8901935
C	4.6364476	-2.0654278	0.2260212
C	3.4836256	-2.0553451	1.0342164
F	-2.0725000	3.3628492	-2.4212236
F	-0.9935849	1.1726287	-0.9360203
F	-0.3823414	6.3190003	0.6012680
F	0.7095301	4.3363872	2.1483305
F	3.4006565	-2.7826647	2.1483305
F	5.6635854	-2.8283828	0.6012680
F	3.9485628	0.1134130	-2.4212236
F	1.5123186	0.2741554	-0.9360203

$[Al(OC_5NF_4)_3F]^-$

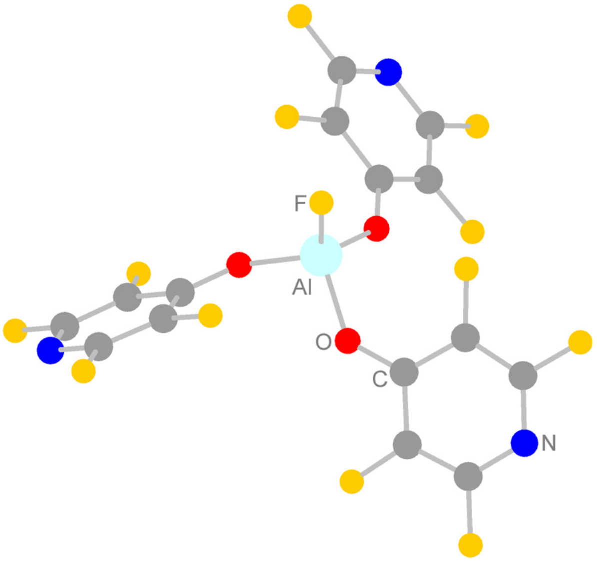

Figure S32. Representation of the BP86/def-SV(P) structure of $[Al(OC_5NF_4)_3F]^-$.

Al	0.0000000	-0.0000000	0.8316552
O	-1.6196791	-0.2338529	0.0970163
C	-2.5014946	-1.1957734	0.0010314
C	-2.4120967	-2.4297915	0.7053752
C	-3.4124653	-3.3939977	0.5434103
N	-4.4682492	-3.2346136	-0.2376634
C	-4.5780345	-2.0957202	-0.9028346
C	-3.6490047	-1.0515814	-0.8316294
F	-1.3599219	-2.6406318	1.5181423
F	-3.8201469	0.0809179	-1.5320303
F	-5.6595236	-1.9481314	-1.6925551
F	-3.3133019	-4.5549691	1.2164779
O	1.0123621	-1.2857568	0.0970163
O	0.6073170	1.5196097	0.0970163
C	0.2151771	2.7642445	0.0010314
C	2.2863175	-1.5684711	0.0010314
C	-0.8982129	3.3038328	0.7053752
C	-1.2330556	4.6522804	0.5434103
N	-0.5671330	5.4869241	-0.2376634
C	0.4740703	5.0125543	-0.9028346
C	0.9138062	3.6859215	-0.8316294
C	2.7351986	-2.6343401	-0.8316294
C	4.1039642	-2.9168341	-0.9028346
N	5.0353822	-2.2523105	-0.2376634
C	4.6455208	-1.2582828	0.5434103
C	3.3103095	-0.8740412	0.7053752
F	1.1426305	5.8753569	-1.6925551
F	1.9801504	3.2678853	-1.5320303
F	-2.2880680	5.1468881	1.2164779
F	-1.6068933	2.4980428	1.5181423
F	2.9668152	0.1425890	1.5181423
F	5.6013699	-0.5919190	1.2164779
F	4.5168931	-3.9272254	-1.6925551
F	1.8399965	-3.3488032	-1.5320303
F	-0.0000000	0.0000000	2.5140458

$[Al(OTeF_5)_5]^{2-}$

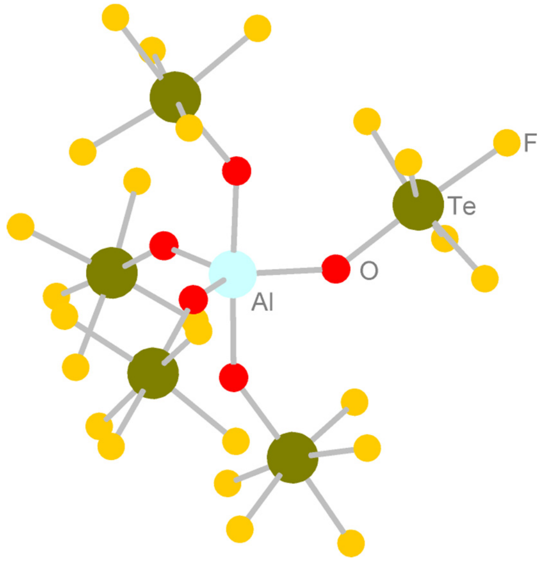

Figure S33. Representation of the B3LYP/def2-TZVPP structure of $[Al(OTeF_5)_5]^{2-}$.

Te	0,5105596	0,2097674	-3,3693125
F	1,2722123	0,1639034	-5,092757
F	-0,9931172	-0,5560967	-4,1795802
F	-0,1260249	1,8980965	-3,8723827
F	2,1191782	1,0047198	-2,8414941
F	1,241698	-1,4977223	-3,1710539
O	-0,2878594	0,2900614	-1,7383464
Al	-0,1104581	0,0674588	0,0589846
O	0,6394392	1,3773747	1,086325
O	-1,7835826	0,9160055	0,2942385
O	-0,8438189	-1,4430569	0,7686635
O	1,585526	-0,7342847	-0,1087974
Te	0,3479381	2,9878033	1,8867397
Te	-3,5377085	0,6952875	-0,1157388
Te	-0,6499978	-3,2007241	1,1888592
Te	3,325461	-0,6933008	0,4095081
F	0,1395619	4,6534154	2,7459717
F	-1,1593332	2,4743515	2,8673436
F	1,8455417	3,773261	1,0847302
F	-0,7199806	3,7260255	0,5406722
F	1,4135136	2,5418539	3,3602724
F	-5,3795054	0,5361168	-0,5017682
F	-4,0508218	2,1959202	0,8853267
F	-3,9346403	-0,3878849	1,3634016
F	-3,3468687	-0,8295662	-1,1890583
F	-3,4890515	1,77453	-1,6492817
F	-1,7064729	-3,0431515	2,7270734
F	-2,1751485	-3,7118501	0,2335631
F	0,3989313	-3,6664886	-0,2856186
F	0,8725196	-2,9797592	2,2529284
F	-0,5253239	-5,0223449	1,6580791
F	5,1438807	-0,706699	0,9173058
F	3,4245367	-2,5482325	0,6511041
F	3,9812262	-0,9370106	-1,3285091
F	2,989578	-0,4806564	2,2409634
F	3,5684119	1,1528771	0,2216447

Me_3SiF

Si	-0.0430541	0.0000935	-0.0022416
C	-0.0396398	-1.6781919	-0.7318168
C	-0.0379295	1.4709838	-1.0909968
C	-0.0530159	0.2074585	1.8159551
H	0.8456071	-1.7981384	-1.3664674
H	-0.9040342	-1.7913157	-1.3954153
H	-0.054228	-2.4690992	0.0161714
H	-0.0180927	1.2184628	-2.1497985
H	0.8254775	2.1017039	-0.852471
H	-0.9240382	2.0811934	-0.8825614
H	0.8170949	-0.3000938	2.2470126
H	-0.9327501	-0.2928952	2.2359021
H	-0.0508851	1.2508097	2.1268189

$[Me_3Si]^+$

Si	2.1627348	1.5902503	-0.000005
F	3.7834859	1.5901311	0.0000253
C	1.6118297	0.8009966	-1.6014181
C	1.6118028	3.3716991	0.1171485
C	1.6117956	0.5981561	1.4842719
H	1.9804593	1.3594345	-2.4640235
H	0.5218225	0.7683298	-1.6675619
H	1.9806113	-0.223221	-1.6840603
H	1.980439	3.9553023	-0.7286194
H	1.980509	3.8395207	1.0320262
H	0.5218018	3.4453311	0.1220027
H	1.9805096	-0.4280842	1.432049
H	0.5217891	0.5571943	1.5455958
H	1.9804996	1.0388194	2.4125588

Et_2O

O	9.7509832	4.4504909	4.1470196
C	9.6653352	4.7833052	5.5269868
H	8.6702542	5.2089301	5.6691391
H	10.3898253	5.5659162	5.7809336
C	11.0659483	4.4279154	3.6058253
H	11.0270135	3.7412781	2.7581965
H	11.7738395	4.0070677	4.3294076
C	11.53192	5.8019206	3.1415388
H	12.5257581	5.7339495	2.6933833
H	11.5884848	6.5096828	3.9702641
H	10.8444546	6.2037712	2.396513
C	9.8480662	3.5729612	6.434031
H	9.1043604	2.8101212	6.2020513
H	9.7311668	3.8628284	7.4807478
H	10.8385598	3.1293315	6.3204223

MeCN

C	-2,2624741	0.2505243	0.0000003
C	-0,8075941	0.2850849	-0.0000054
H	-2,6559594	0.9678295	-0.7200274
H	-2,6448811	0.5018635	0.98914
H	-2,6152791	-0.7449176	-0.2691135
N	0,3417778	0.3124055	0.0000061

PhCN

H	1.231193	-0.1446656	2.1729407
C	0.6976391	-0.0845799	1.2335991
C	-0.6928983	-0.0352508	1.2269253
H	-1.2416318	-0.0569419	2.1588878
C	-1.3824109	0.0418629	0.0257527
H	-2.4626363	0.0804166	0.0110547
C	-0.6737847	0.0699336	-1.1806489
C	-1.3795669	0.1489613	-2.4231125
C	0.7247115	0.0200587	-1.1725804
H	1.2647767	0.0419339	-2.1087689
C	1.4033911	-0.0570275	0.0347465
H	2.4844177	-0.0956815	0.0398024
N	-1.9481801	0.2120104	-3.4239384

PhF

F	0,3588202	0.5953748	1.2101155
H	4,2143961	0.5085417	3.5115361
C	3,1746920	0.8050701	3.4678883
C	2,5977334	1.4859875	4.5350857
H	3,1874662	1.7206633	5.4108424
C	1,2604044	1.8643153	4.4731636
H	0,8072286	2.3939943	5.3007324
C	0,4947210	1.5665536	3.3506504
H	-0,5460632	1.8497665	3.2786951
C	1,0957903	0.8884978	2.3045735
C	2,4235442	0.4995298	2.3376726
H	2,8490170	-0.0281946	1.4956043

PhMe

C	-0.755666	0.5851012	0.0718004
C	-0.9738618	-0.7881163	0.0611556
C	-2.269066	-1.2895158	-0.0070361
C	-3.3411732	-0.4057634	-0.0630452
C	-3.1170585	0.9665212	-0.0522429
C	-1.8220905	1.4843593	0.0128254
C	-1.5808786	2.9718951	-0.0042481
H	0.2575452	0.9649963	0.1300163
H	-0.1315859	-1.4664511	0.1102131
H	-2.4413621	-2.3578377	-0.0119766
H	-4.3542128	-0.7844467	-0.1112206
H	-3.9601134	1.6462393	-0.0916641
H	-1.465245	3.3394682	-1.0276329
H	-0.6727555	3.2343726	0.5393265
H	-2.4132659	3.5154776	0.4437092
